# Supplementary material for: Educational differentials in key domains of physical activity by ethnicity, age and sex: a cross-sectional study of over 40 000 participants in the UK household longitudinal study (2013–2015)
Source: BMJ Open. 2020 Jan 20;10(1):e033318. doi: 10.1136/bmjopen-2019-033318 (PMC7045199; doi:10.1136/bmjopen-2019-033318)
Supplement: Supplementary data [file bmjopen-2019-033318supp001.pdf]

Table S1. Ethnicity, sex and age by highest educational attainment in Understanding Society (2013-2015)

Demographics	Highest educational attainment					
	GCSEs and lower		School diploma & other qualifications		Degree/higher	
	N	*(%)*	N	*(%)*	N	*(%)*
**Ethnicity**						
White	11638	33.7	10424	30.2	12424	35.9
Black	363	25.1	400	27.6	685	47.3
Asian	1106	32.2	877	25.5	1445	42.1
Other ethnicity	201	22.1	235	25.9	472	51.9
Missing	538	36.1	491	33	401	26.9
P value	<0.001					
**Sex**						
Male	5888	30.5	6388	33.1	6927	35.9
Female	7958	35.3	6039	26.8	8500	37.7
P value	<0.001					
**Age**						
20-29 years	1462	23.9	2363	38.6	2271	37.1
30-39 years	1653	24.3	1776	26.1	3345	49.2
40-49 years	2433	29.1	2339	28	3570	42.7
50-59 years	2455	32.6	2221	29.5	2822	37.5
60+	5843	44.8	3728	28.6	3419	26.2
P value	<0.001					

Participants from wave 5 (2013-2015) of Understanding Society with data on educational attainment, demographics, and physical activity
Missing = missing data; not displayed where data was available in complete.
P value = chi^{2},

Table S2. Estimated difference in predicted probability in educational differences in physical activity by age

Physical activity	20-29				30-39				40-49				50-59				60+				education X age
	Coef.	*95% CI*		*p*	*Coef.*	*95% CI*		*p*	*Coef.*	*95% CI*		*p*	*Coef.*	*95% CI*		*p*	*Coef.*	*95% CI*		*p*	
**Active Travel**																					
GCSEs and lower	**ref*																				
School diploma	-0.03	-0.07	0.01	0.206	-0.04	-0.07	0.00	0.064	-0.01	-0.04	0.02	0.506	-0.03	-0.06	0.00	0.091	-0.04	-0.08	0.01	0.155	0.081
Degree/higher	-0.04	-0.08	0.00	0.044	-0.07	-0.10	-0.04	<0.001	-0.08	-0.10	-0.05	<0.001	-0.07	-0.10	-0.04	<0.001	-0.06	-0.11	-0.01	0.012	
**Occupational**																					
GCSEs and lower	**ref*																				
School diploma	-0.10	-0.14	-0.06	<0.001	-0.14	-0.18	-0.10	<0.001	-0.06	-0.10	-0.03	<0.001	-0.03	-0.07	0.00	0.060	-0.01	-0.06	0.04	0.648	0.001
Degree/higher	-0.25	-0.29	-0.21	<0.001	-0.29	-0.33	-0.26	<0.001	-0.26	-0.29	-0.23	<0.001	-0.24	-0.27	-0.21	<0.001	-0.20	-0.25	-0.15	<0.001	
**Moderate-to-vigorous leisure time**																					
GCSEs and lower	**ref*																				
School diploma	0.09	0.06	0.13	<0.001	0.08	0.04	0.11	<0.001	0.04	0.02	0.07	0.001	0.06	0.04	0.09	<0.001	0.06	0.04	0.08	<0.001	0.008
Degree/higher	0.17	0.14	0.21	<0.001	0.17	0.14	0.20	<0.001	0.16	0.14	0.19	<0.001	0.18	0.16	0.21	<0.001	0.16	0.14	0.18	<0.001	
**Light leisure time**																					
GCSEs and lower	**ref*																				
School diploma	0.02	-0.01	0.04	0.173	0.03	0.00	0.06	0.030	0.04	0.01	0.06	0.002	0.02	-0.01	0.04	0.180	0.06	0.04	0.08	<0.001	<0.001
Degree/higher	0.02	0.00	0.05	0.102	0.03	0.01	0.05	0.007	0.03	0.01	0.06	0.002	0.08	0.06	0.11	<0.001	0.13	0.11	0.15	<0.001	

Estimates are derived from separate logistic regression models of each binary physical activity outcome including a two-way interaction terms (education x age), and show the education-outcome associations on the absolute scale in each sociodemographic sub group
Participants from wave 5 (2013-2015) of Understanding Society with data on educational attainment, demographics, and physical activity
**reference groups*

Table S3. Estimated difference in predicted probability in educational differences in physical activity by sex

Physical activity	Male				Female				education X sex
	Coef.	*95% CI*		*p*	*Coef.*	*95% CI*		*p*	
**Active Travel**									
GCSEs and lower	**ref*								
School diploma	-0.02	-0.05	0.00	0.050	-0.03	-0.05	0.00	0.027	<0.001
Degree/higher	-0.03	-0.05	0.00	0.018	-0.10	-0.12	-0.08	0.000	
**Occupational**									
GCSEs and lower	**ref*								
School diploma	-0.06	-0.09	-0.04	<0.001	-0.08	-0.11	-0.06	<0.001	<0.001
Degree/higher	-0.35	-0.37	-0.32	<0.001	-0.17	-0.19	-0.15	<0.001	
**Moderate-to-vigorous leisure time**									
GCSEs and lower	**ref*								
School diploma	0.06	0.05	0.08	<0.001	0.07	0.05	0.08	<0.001	0.550
Degree/higher	0.17	0.15	0.18	<0.001	0.17	0.15	0.18	<0.001	
**Light leisure time**									
GCSEs and lower	**ref*								
School diploma	0.03	0.01	0.04	0.001	0.05	0.03	0.06	<0.001	<0.001
Degree/higher	0.02	0.01	0.03	0.005	0.12	0.10	0.13	<0.001	

Estimates are derived from separate logistic regression models of each binary physical activity outcome including a two-way interaction terms (education x sex), and show the education-outcome associations on the absolute scale in each sociodemographic sub group

Participants from wave 5 (2013-2015) of Understanding Society with data on educational attainment, demographics, and physical activity

**reference group*

Table S4. Estimated difference in predicted probability in educational differences in physical activity by ethnicity

Physical activity	White				Black				Asian				Other ethnicity				education X ethnicity
	Coef.	*95% CI*		*p*	*Coef.*	*95% CI*		*p*	*Coef.*	*95% CI*		*p*	*Coef.*	*95% CI*		*p*	P
**Active Travel**																	
GCSEs and lower	**ref*																
School diploma	-0.02	-0.04	0.00	0.013	0.02	-0.08	0.11	0.723	-0.07	-0.13	0.00	0.040	-0.04	-0.17	0.08	0.523	0.038
Degree/higher	-0.06	-0.08	-0.04	<0.001	-0.12	-0.20	-0.05	0.002	-0.11	-0.17	-0.05	<0.001	-0.04	-0.15	0.07	0.465	
**Occupational**																	
GCSEs and lower	**ref*																
School diploma	-0.08	-0.10	-0.06	<0.001	-0.03	-0.12	0.06	0.526	-0.07	-0.13	0.00	0.035	-0.04	-0.17	0.08	0.502	0.155
Degree/higher	-0.27	-0.29	-0.25	<0.001	-0.14	-0.22	-0.06	0.001	-0.18	-0.24	-0.13	<0.001	-0.19	-0.30	-0.08	0.001	
**Moderate-to-vigorous leisure time**																	
GCSEs and lower	**ref*																
School diploma	0.06	0.05	0.08	<0.001	0.02	-0.04	0.08	0.488	0.09	0.06	0.12	<0.001	0.12	0.04	0.20	0.004	0.001
Degree/higher	0.17	0.16	0.19	<0.001	0.06	0.01	0.12	0.030	0.16	0.13	0.19	<0.001	0.13	0.06	0.20	<0.001	
**Light leisure time**																	
GCSEs and lower	**ref*																
School diploma	0.04	0.02	0.05	<0.001	0.05	0.00	0.09	0.046	0.04	0.01	0.07	0.008	0.08	0.01	0.14	0.026	0.057
Degree/higher	0.07	0.06	0.08	<0.001	0.02	-0.02	0.06	0.351	0.07	0.05	0.10	<0.001	0.12	0.06	0.17	<0.001	

Estimates are derived from separate logistic regression models of each binary physical activity outcome including a two-way interaction terms (education x ethnicity), and show the education-outcome associations on the absolute scale in each sociodemographic sub group
Participants from wave 5 (2013-2015) of Understanding Society with data on educational attainment, demographics, and physical activity
**reference group*

Table S5. Unadjusted associations (OR (95% CI)) of education and demographics with domain specific PA (complete case analysis)

	Physical Activity															
	Active travel				Occupational				Leisure							
									Moderate-to-vigorous				Light			
	N=18,404				*N=22,287*				*N= 40,270*				*N= 40,270*			
	OR	*95% CI*		*p*	*OR*	*95% CI*		*p*	*OR*	*95% CI*		*p*	*OR*	*95% CI*		*p*
**Education**																
GCSEs and lower	**ref*															
School diploma	0.86	0.77	0.96	0.006	0.75	0.69	0.81	<0.001	1.59	1.50	1.69	<0.001	1.23	1.16	1.31	<0.001
Degree/higher	0.62	0.55	0.68	<0.001	0.35	0.33	0.38	<0.001	2.55	2.41	2.69	<0.001	1.41	1.33	1.50	<0.001
**Ethnicity**																
White	**ref*															
Black	0.89	0.71	1.13	0.358	1.87	1.59	2.20	<0.001	0.84	0.75	0.95	0.005	0.38	0.31	0.45	<0.001
Asian	1.07	0.92	1.25	0.388	1.38	1.23	1.55	<0.001	0.74	0.68	0.81	<0.001	0.47	0.42	0.52	<0.001
Other ethnicity	1.25	0.97	1.61	0.080	1.20	0.99	1.44	0.061	1.08	0.94	1.25	0.277	0.66	0.55	0.80	<0.001
**Sex**																
Male	**ref*															
Female	1.09	1.01	1.19	0.033	0.99	0.94	1.05	0.822	0.85	0.82	0.89	<0.001	1.17	1.12	1.23	<0.001
**Age**																
20-29 years	**ref*															
30-39 years	0.75	0.65	0.85	<0.001	0.68	0.62	0.75	<0.001	1.01	0.93	1.09	0.875	1.05	0.95	1.16	0.353
40-49 years	0.68	0.60	0.77	<0.001	0.69	0.63	0.76	<0.001	0.84	0.78	0.90	<0.001	1.38	1.25	1.51	<0.001
50-59 years	0.76	0.67	0.87	<0.001	0.78	0.71	0.85	<0.001	0.60	0.56	0.65	<0.001	1.56	1.42	1.71	<0.001
60+ years	0.70	0.58	0.83	<0.001	0.88	0.79	0.99	0.034	0.43	0.39	0.46	<0.001	1.64	1.50	1.80	<0.001

Participants from wave 5 (2013-2015) of Understanding Society with data on educational attainment, demographics, and physical activity
Samples were restricted to those with valid demographic and physical activity data
*Active travel to work: non-active/active; Occupational: non-physical/physical; Leisure-time: <weekly/ ≥ 1x weekly*reference groups*
OR= Odds Ratio, CI= 95% Confidence intervals

Table S6. Unadjusted associations (OR (95% CI)) of education and demographics with domain specific PA (maximum available samples)

	Physical Activity																			
	Active travel					Occupational					Leisure									
											Moderate-to-vigorous					Light				
	N	*OR*	*95% CI*		*p*	N	*OR*	*95% CI*		*p*	N	*OR*	*95% CI*		*p*	N	*OR*	*95% CI*		*p*
**Education**																				
GCSEs and lower	18535	**ref*				22431					41700					41700				
School diploma		0.86	0.77	0.96	0.006		0.75	0.69	0.81	<0.001		1.59	1.50	1.69	<0.001		1.23	1.16	1.31	<0.001
Degree/higher		0.62	0.55	0.69	<0.001		0.35	0.33	0.38	<0.001		2.56	2.43	2.70	<0.001		1.43	1.35	1.52	<0.001
**Ethnicity**																				
White	18430	**ref*				22317					40364					40364				
Black		0.89	0.71	1.13	0.360		1.87	1.59	2.20	<0.001		0.84	0.75	0.95	0.005		0.38	0.31	0.45	<0.001
Asian		1.07	0.92	1.25	0.385		1.38	1.23	1.55	<0.001		0.74	0.68	0.81	<0.001		0.47	0.42	0.52	<0.001
Other ethnicity		1.25	0.97	1.61	0.080		1.20	0.99	1.44	0.062		1.08	0.94	1.25	0.282		0.66	0.55	0.80	<0.001
**Sex**																				
Male	18568	**ref*				22470					41853					41853				
Female		1.09	1.00	1.18	0.039		0.99	0.94	1.04	0.688		0.90	0.86	0.93	<0.001		1.22	1.17	1.28	<0.001
**Age**																				
20-29 years	18568	**ref*				22470					41853					41853				
30-39 years		0.74	0.65	0.85	<0.001		0.68	0.62	0.75	<0.001		1.04	0.96	1.12	0.380		1.07	0.97	1.18	0.192
40-49 years		0.68	0.60	0.77	<0.001		0.69	0.63	0.75	<0.001		0.87	0.81	0.94	<0.001		1.40	1.28	1.54	<0.001
50-59 years		0.76	0.67	0.87	<0.001		0.77	0.71	0.84	<0.001		0.63	0.59	0.68	<0.001		1.58	1.45	1.74	<0.001
60+ years		0.70	0.59	0.84	<0.001		0.88	0.79	0.99	0.031		0.45	0.42	0.48	<0.001		1.69	1.54	1.84	<0.001

Participants from wave 5 (2013-2015) of Understanding Society with data on educational attainment, demographics, and physical activity
Sample size was not restricted and represents total available sample
*Active travel to work: non-active/active; Occupational: non-physical/physical; Leisure-time: <weekly/ ≥ 1x weekly*reference groups*
OR= Odds Ratio, CI= 95% Confidence intervals

Figure S1. Flow diagram of analyses in Understanding Society

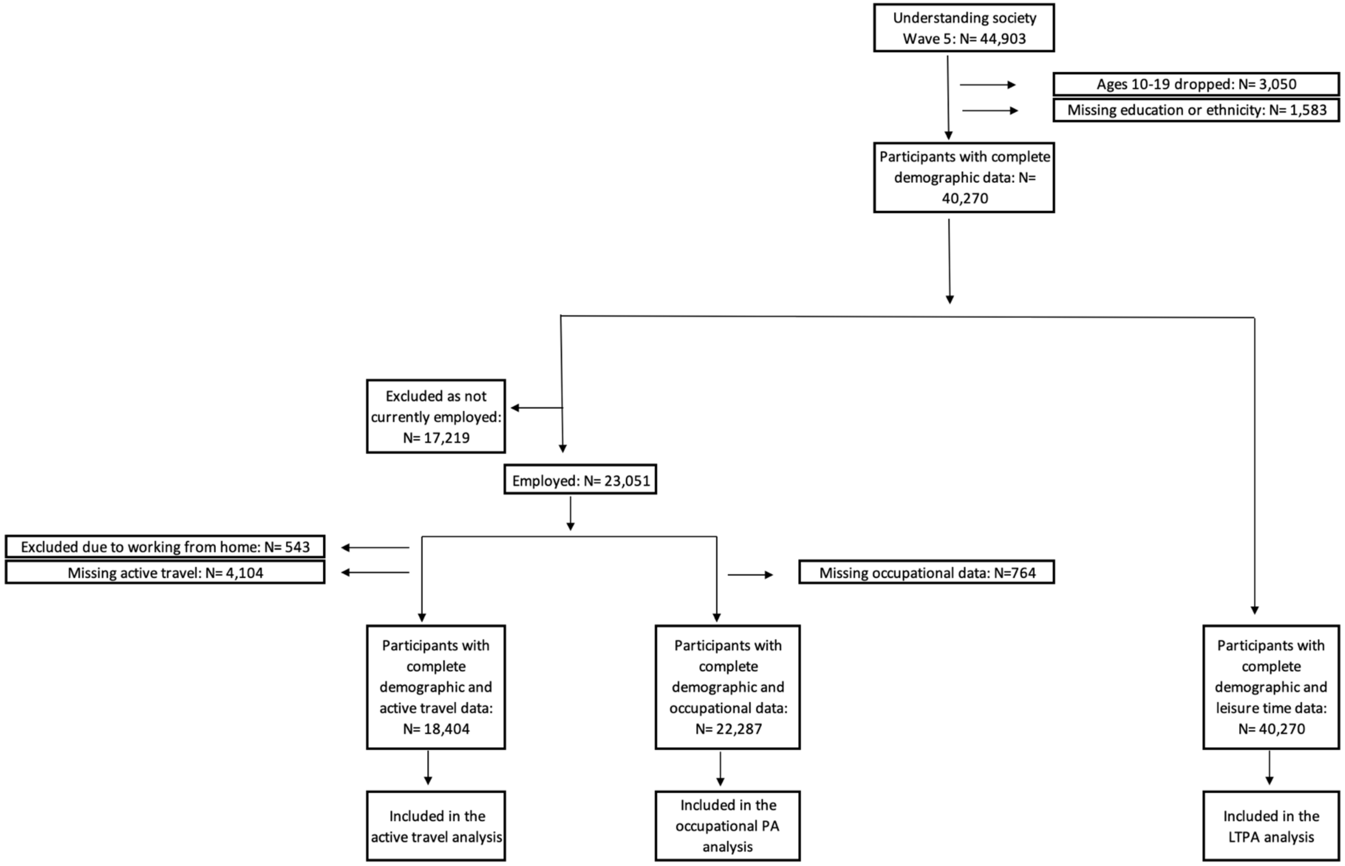

There was no missing data for sex and age demographics.
Active travel and occupational physical activity only include individuals who are currently employed, due to questionnaire routing.
