# Supplementary material for: Educational differentials in key domains of physical activity by ethnicity, age and sex: a cross-sectional study of over 40 000 participants in the UK household longitudinal study (2013–2015)
Source: BMJ Open. 2020 Jan 20;10(1):e033318. doi: 10.1136/bmjopen-2019-033318 (PMC7045199; doi:10.1136/bmjopen-2019-033318)
Supplement: Author's manuscript [file bmjopen-2019-033318.draft_revisions.pdf]

BMJ Open is committed to open peer review. As part of this commitment we make the peer review history of every article we publish publicly available.

When an article is published we post the peer reviewers' comments and the authors' responses online. We also post the versions of the paper that were used during peer review. These are the versions that the peer review comments apply to.

The versions of the paper that follow are the versions that were submitted during the peer review process. They are not the versions of record or the final published versions. They should not be cited or distributed as the published version of this manuscript.

BMJ Open is an open access journal and the full, final, typeset and author-corrected version of record of the manuscript is available on our site with no access controls, subscription charges or pay-per-view fees (http://bmjopen.bmj.com).

If you have any questions on BMJ Open's open peer review process please email info.bmjopen@bmj.com

BMJ Open

Educational differentials in key domains of physical activity by ethnicity, age, and sex: a cross-sectional study of over 44,000 participants in The UK Household Longitudinal Study (2013-2015)

Journal:	*BMJ Open*
Manuscript ID	bmjopen-2019-033318
Article Type:	Research
Date Submitted by the Author:	30-Jul-2019
Complete List of Authors:	Fluharty, Meg; University College London, Centre for Longitudinal Studies<br>Pinto Pereira, Snehal; University College London Institute of Child Health, UCL Research Department of Epidemiology & Public Health<br>Benzeval, Michaela; University of Essex, Institute for Social and Economic Research<br>Hamer, Mark; University of Loughborough, School of Sport, Exercise and Health Science<br>Jefferis, Barbara ; University College London, Department of Primary Care and Population Health<br>Griffiths, Lucy; Swansea University Medical School, Health Data Research UK, Wales and Northern Ireland<br>Cooper, Rachel; Manchester Metropolitan University, Sport and Exercise Sciences<br>Bann, David; University College London, Centre for Longitudinal Studies
Keywords:	health disparities, EPIDEMIOLOGY, physical activity

SCHOLARONE™
Manuscripts

I, the Submitting Author has the right to grant and does grant on behalf of all authors of the Work (as defined in the below author licence), an exclusive licence and/or a non-exclusive licence for contributions from authors who are: i) UK Crown employees; ii) where BMJ has agreed a CC-BY licence shall apply, and/or iii) in accordance with the terms applicable for US Federal Government officers or employees acting as part of their official duties; on a worldwide, perpetual, irrevocable, royalty-free basis to BMJ Publishing Group Ltd ("BMJ") its licensees and where the relevant Journal is co-owned by BMJ to the co-owners of the Journal, to publish the Work in this journal and any other BMJ products and to exploit all rights, as set out in our licence.

The Submitting Author accepts and understands that any supply made under these terms is made by BMJ to the Submitting Author unless you are acting as an employee on behalf of your employer or a postgraduate student of an affiliated institution which is paying any applicable article publishing charge ("APC") for Open Access articles. Where the Submitting Author wishes to make the Work available on an Open Access basis (and intends to pay the relevant APC), the terms of reuse of such Open Access shall be governed by a Creative Commons licence – details of these licences and which Creative Commons licence will apply to this Work are set out in our licence referred to above.

Other than as permitted in any relevant BMJ Author's Self Archiving Policies, I confirm this Work has not been accepted for publication elsewhere, is not being considered for publication elsewhere and does not duplicate material already published. I confirm all authors consent to publication of this Work and authorise the granting of this licence.

For peer review only

Running Head: Educational differentials in physical activity

Educational differentials in key domains of physical activity by ethnicity, age, and sex: a cross-sectional study of over 44,000 participants in The UK Household Longitudinal Study (2013-2015)

Meg E Fluharty[1], Snehal M Pinto Pereira[2], Michaela Benzeval[3], Mark Hamer[4], Barbara Jefferis[5], Lucy Griffiths[6], Rachel Cooper[7] David Bann[1]

1. Centre for Longitudinal Studies, Institute of Education, University College London
2. UCL Research Department of Epidemiology & Public Health, University College London
3. Institute for Social and Economic Research, University of Essex
4. School of Sport, Exercise and Health Sciences, Loughborough University
5. Department of Primary Care and Population Health, University College London
6. Health Data Research UK, Wales and Northern Ireland, Swansea University Medical School
7. Musculoskeletal Science and Sports Medicine Research Centre, Department of Sport and Exercise Sciences, Faculty of Science and Engineering, Manchester Metropolitan University

Corresponding author Meg Fluharty, Centre for Longitudinal Studies, University College London, 55-59 Gordon Square, London WC1H 0NU, United Kingdom. Email: m.fluharty@ucl.ac.uk

For peer review only

Abstract

Objectives: To assess whether educational differentials in three key physical activity (PA) domains vary by age, sex and ethnicity

Design: Nationally representative cross-sectional survey

Setting: UK

Participants: Up to 44,903 participants, aged 20 years and over, from the UK Household Longitudinal Study with information on education, PA and demographics collected in 2013-2015 (N= 44,903).

Primary and secondary outcome measures: Participation in active travel, occupational activity and leisure time physical activity at the time of assessment

Results: Lower educational attainment was associated with higher AT and OA, but lower weekly LTPA activity; associations were modified by sex, ethnicity, and age. For example, education-related differences in AT were larger for females (difference in predicted probability of activity between highest and lowest educational groups: -10% in females, (95% CI: -11.9, 7.9) -3% in males (-4.8, -0.4). The education-related differences in OA were larger among males -35% (-36.9, -32.4) than females -17% (-19.4, -15.0). Finally, Education related differences in moderate-to-vigorous LTPA varied by ethnicity; for example, differences were 17% (16.2, 18.7) for White individuals compared with 6% (0.6, 11.6) for Black individuals.

Conclusions: Educational differences in PA vary by domain, and are modified by age, sex, and ethnicity. A better understanding of physically inactive sub-groups may aid development of tailored interventions to increase activity levels and reduce health inequalities.

For peer review only

Strengths and limitations of this study:

1. This study utilizes include a large nationally representative sample, enabling us to identify the previously seldom-examined role of ethnicity as a predictor of physical activity and modifier of the relationship between educational attainment and physical activity.

2. This study examined activity outcomes across three domains; previous studies investigating associations of physical activity typically use a single physical activity outcome measure, capturing either 'leisure' or 'unspecified' activity

3. All physical activity measures were captured via self-report, which may be subject to recall bias with individuals' either over or under reporting their levels of physical activity

4. Missing data may introduce bias, although missing data due to item missingness (as opposed to specific question gating) was low and therefore bias unlikely

5 This study identified cross-sectional associations of education with physical activity across key domains; however, further longitudinal evidence is required to provide stronger evidence of causality and investigate the mediators of the observed associations, associations of educational-attainment with physical activity may be partially explained by reverse causality.

For peer review only

For peer review only - http://bmjopen.bmj.com/site/about/guidelines.xhtml

Introduction

Physical activity is an important modifiable determinant of health. [1] Leisure time physical activity's (LTPA) benefits are particularly well-documented and include improvements in the musculoskeletal system, maintenance of healthy weight, protection against cardiovascular disease, and reduction in symptoms of depression and anxiety[1]. However, there is a global trend towards high levels of leisure time physical inactivity which is estimated to contribute to ~6-10% of major non-communicable diseases, ~5.3 million deaths annually, [2] and ~$67.5 billion per year in health care expenditure. [3]

Physical activity can be accrued through multiple domains (e.g. active travel, leisure time, occupation, and housework/DIY), which may have differing impacts on health outcomes. [4] For example, LTPA is thought to be beneficial to physical health and wellbeing, while labour-intense occupations may increase risk of musculoskeletal strain. [5 6] Therefore, examining these different domains may provide evidence to help inform where possible interventions could be targeted. Understanding what is driving differences in activity participation overall, as well as in different domains of physical activity, may also help to identify which forms of activity could be intervened on to reduce socioeconomic disparities in health.

Recent systematic reviews find evidence of socioeconomic disparities in LTPA in high-income countries [7] that have persisted across recent decades. [8] Additionally, lower education has been shown to be associated with higher risk of future declines in LTPA. [9 10] Alongside indicators of socioeconomic position, a number of other sociodemographic factors, including ethnicity, sex, and age have been shown to be associated with physical activity. [11] For example, differences in the levels of PA participation have been reported across ethnic groups in the UK, with those of 'mixed' ethnicity having the highest prevalence of activity, [12 13] and South Asians the lowest. [13 14] Numerous factors may explain these differences including personal, socioeconomic, cultural, and environmental factors. [12 13] Alongside ethnic differences, physical activity levels are consistently found to be lower for women than men and for older adults. [15]

Educational disparities in physical activity may arise though a number of routes including due to differences in knowledge of the health impacts of LTPA, material pathways, and potentially due to selection into neighbourhoods which differ in their suitability for outdoor physical activity. [16 17] Educational differences in physical activity participation may also be modified by ethnicity, age, and sex. [4 18] For example, low-level education occupations that men tend to be employed in are usually more physically demanding than equivalent roles women tend to be employed in; [4 19] this contrasts

For peer review only

with the lower engagement in leisure-time activities often observed among men with lower levels of education. [20] Moreover, evidence from the US has indicated education-related disparities across activity domains. [4] However, these associations have not yet been investigated within the UK. Previous studies that have investigated associations of different indicators of socioeconomic position including education with physical activity outcomes are limited by only investigating one specific domain, [21,22] or use population samples from specific regions within the UK. [23,24] Thus, important gaps remain in our understanding of the nature of socioeconomic inequalities in physical activity outcomes. These are important to fill given their purported mediating role in socioeconomic inequalities in many important health outcomes including premature mortality. [25]

We sought to address the above-mentioned gaps in the literature by investigating educational disparities in physical activity across active travel, leisure, and occupational domains. Additionally, we aimed to examine if associations between education and domain specific physical activity were modified by ethnicity, age, and sex. We hypothesised that lower education status would be associated with lower levels of participation in physical activity during leisure time, but higher participation in active travel and occupational activity and that these associations would be modified by ethnicity, age and sex. A large household panel study was used (Understanding Society), which benefits from national representation, oversampling of ethnic minority groups, and detailed measures of domain-specific physical activity.

For peer review only

For peer review only - http://bmjopen.bmj.com/site/about/guidelines.xhtml

Methods

Participants

Understanding Society: the UK Household Longitudinal Study (UKHLS) is a nationally representative study which started in 2009 aiming to recruit individuals in 40,000 households .[26] The study annually samples all individuals in the household over the age of 10. Additionally, sample members are followed when they leave the household, and new individuals join the study as they become part of an existing study member's household. Information is collected from participants on a range of information including wellbeing, health, home, family and employment. Detailed study information and sampling methodology can be found elsewhere. [26] All participants consent for use of their anonymised survey information.

The sample for our analysis includes adult (20 years or over) responders who took part in Wave 5 (2013-2015) and responded to demographic and physical activity questions via interviewer led and self-completed questionnaire. Wave 5 was chosen as this was the most recent wave of data collection including physical activity questions.

Patient and public involvement

This study used publicly available secondary data from the UK Data Service (https://www.ukdataservice.ac.uk/). Patients and the public were not involved.

Measures

Domain specific physical activity

Active travel was measured in currently employed individuals and those not working from home via the question '*how do you usually get to your place of work?'* Responses were collapsed into a binary variable of 'non-active' (car, bus, or train/metro) or 'active' (walking or cycling). Occupational physical activity was measured by asking participants whether their job was mainly physical or not (categorised as 'not physical' and 'physical'). Finally, LTPA variables were created from participant responses to the 'Taking part Survey' [27] which indicated how often they participated in a series of prelisted sports and activities. Sports were then grouped into two categories based on their average metabolic equivalent of task (MET), those with METs of ≥3 were categorised as moderate-to-vigorous and METs 1.5-2.9 as light. [28] Frequency of participation in each MET-group was categorised as weekly or non-weekly.

For peer review only - http://bmjopen.bmj.com/site/about/guidelines.xhtml

Socio demographics

Highest educational attainment was self-reported and categorised into three groups: 'degree or higher, 'school diploma/ other qualification' (e.g. A levels and vocational diplomas) and 'GCSEs and below'. Ethnicity was self-reported and responses were collapsed into 'White', 'Black', 'Asian' and 'other ethnicity.' These broad ethnic groupings include minority groups (e.g. 'White' includes all white minorities such as Irish and Polish, Black includes Black-African and Black-Caribbean, and those of smaller sample sizes such as Arab and mixed-ethnicity were included in 'other'). Age at the time of interview was categorised into ten year age groups (from ages 20-60). Older adults were grouped from >60 years, and those below 20 (N= 3,050) were excluded from the analysis to ensure comparable sample sizes in the higher education groups—alternative groupings did not substantially affect the results (data available upon request).

Statistical analysis

We first cross-tabulated educational attainment by age, sex, and ethnicity. Next, logistic regression analyses were conducted to examine associations of education, sex, age, and ethnicity with physical activity in each domain. Analyses were assessed before and after mutual adjustment for each demographic variable. Those with missing demographic and education data yet valid outcome data were excluded from analysis (N= 1,642); analytical samples for active travel, occupational, and leisure were N= 18,404, N= 22,287 and N= 40,270 respectively. The sample sizes differences by outcome was largely due to routing - only employed individuals were asked about occupational activity or active travel. A flow diagram (Supplementary Figure S1) displays the final sample size for each outcome. Finally, to examine possible effect modification of the associations between education and physical activity in each domain, we included two-way interaction terms (education x ethnicity; education x sex; education x age) in addition to the relevant first order terms in the same model. Analyses were weighted according to sample design and attrition. [26] All analyses were conducted using Stata, version 15.0 (StataCorp LP, College Station, Texas).

For peer review only - http://bmjopen.bmj.com/site/about/guidelines.xhtml

Results

Ethnicity, sex, and age were each independently associated with educational attainment and with physical activity in each domain (p< 0.001; see Table 1 and Supplementary Table S1). Lower educational attainment was associated with higher active travel and occupational physical activity, but lower weekly light and moderate LTPA.

Active travel to work

Active travel was lowest amongst individuals who were highly-educated, older, and male; there was little evidence for associations with ethnicity (See Table 2). The magnitude of education-related disparities were largest among females (education x sex P<0.001) and Black individuals (education x ethnicity P= 0.038) (See Figure 1 & Supplementary Tables S2-4). For example, the estimated difference in the probability of using active travel in the highest versus the lowest educational group was -10% (95% CI: -11.9, 7.9) amongst females and -3% (-4.8, -0.4) among males. Results for this domain, and all others, were similar when restricting to those with valid demographic and physical activity data, or when not making this restriction (Supplementary tables S5-6).

Occupational activity

Physically active occupations were less commonly reported amongst individuals who were highly-educated, white, and aged over 20-29; there was no evidence for association with sex (see Table 2). The magnitude of education-related disparities were largest among men (education x sex P <0.001), and those aged 30-39 (education x age P= 0.001) (See Figure 2 and Supplementary Tables S2-4). For example, the estimated difference in the probability of a physical occupation in the highest versus lowest educational group was -35% (-36.9, -32.4) for males and -17% (-19.4, -15.0) for females.

Moderate-to-vigorous and light LTPA

Greater levels of participation in both weekly light and moderate-to-vigorous LTPA were reported amongst individuals who were highly educated and white. Males and younger adults were also more likely to report participation in moderate-to-vigorous LTPA, whereas females and older adult were more likely to report participation in light LTPA. (See Table 2).

The magnitude of education-related disparities in weekly moderate-to-vigorous LTPA was largest among White and Asian individuals (education x ethnicity P= 0.001), and those aged 40-49 and 50-59 (education x age P= 0.008) (See Figures 3-4 and Supplementary Tables S2-4). For example, the estimated probability of weekly moderate-to-vigorous LTPA in the highest versus lowest educational

For peer review only - http://bmjopen.bmj.com/site/about/guidelines.xhtml

group was 17% (16.2, 18.7) for white individuals, compared with 6% (0.6, 11.6) for Black individuals, 16% (12.8, 19.1) for Asian individuals, and 13% (6.0, 19.5) for those of other ethnicity.

The magnitude of education-related disparities in weekly light LTPA was largest among females (education x sex P< 0.001) and individuals aged 60+ (education x age P< 0.001); there was little evidence for associations with ethnicity (See Figures 3-4 and Supplementary Tables S2-4). For example, the estimated probability of weekly light leisure time activity in the highest versus lowest educational group was 13% (11.4, 15.3) for those aged 60+ compared with 8% (5.9, 10.6) for those ages 50-59, 3% (1.3, 5.6) for those 40-49, 3% (0.9, 5.4) for those 30-39, and 2% (-.04, 4.7) for those aged 20-29.

For peer review only

For peer review only - http://bmjopen.bmj.com/site/about/guidelines.xhtml

Discussion

Main findings and interpretations

In a large nationally representative dataset, educational attainment was associated with physical activity across three key domains; individuals with higher education were less likely to engage in active travel and occupational physical activity, but were more likely to engage in LTPA. These associations were modified by ethnicity, age, and sex. For active travel, educational disparities were largest among females and Black individuals. For occupational physical activity disparities were largest among males and those aged 30-39. For moderate-to-vigorous LTPA, educational disparities were largest among White and Asian individuals and those aged 40-49 and 50-59. Finally, for light LTPA, disparities were largest among females and those aged 60+.

Previous evidence suggests a number of possible disparities such as health status [29], environment [30], cultural preferences, [31] financial resources, [25] perceived safety, [32] and domestic requirements [15 33] may exist within each sociodemographic group or a given level of educational attainment. These differences may in turn explain the observed modification of education disparities in physical activity. These potential explanations require future empirical investigation.

Strengths and Limitations

Strengths of this study include a large nationally representative sample, enabling us to identify the previously seldom-examined role of ethnicity as a modifier of the relationship between educational attainment and physical activity across different domains. We also examined activity outcomes across three domains; previous studies investigating associations of physical activity typically use a single physical activity outcome measure, capturing either 'leisure' or 'unspecified' activity. [4 10]

There are also a number of limitations to consider. First, while we obtained information across multiple domains, we lack detailed information on activity duration. Nor did we consider perceptions of the local environment including safety which may affect physical activity. [32 34] However, the LTPA measures used followed expected patterns for these leisure time categories by sex and age. [35] Second, we did not capture physical housework as a domain, which includes domestic and cleaning tasks, gardening, and DIY. Third, all physical-activity measures were captured via self-report; while this is needed to investigate domain-specific activity, it may be subject to recall bias with individuals' either over or under reporting their levels of physical activity. [36] For example, previous evidence has shown that males and those who achieved fewer years in education were more likely to overestimate their physical activity levels than females and those with greater levels

For peer review only - http://bmjopen.bmj.com/site/about/guidelines.xhtml

of education, respectively. [37] Additionally, measurement error in self-reported physical activity may account for differential reporting bias across cultures and social strata. [38] Fourth, only working adults could be included in the analyses of the active travel and occupational domains, therefore, investigation of multiple types of physical activity among retirees, those currently seeking work, or those unable to work warrants consideration in the future. Fifth, bias may be introduced through excluding missing data and non-responders, although missing data due to item missingness (as opposed to specific question gating) was low and therefore bias unlikely. Sixth, due to the cross-sectional design we cannot separate out age from birth cohort, and future cross-cohort studies are therefore required to address this.

Finally, this study identified cross-sectional associations of education with physical activity across key domains, as well as differences by ethnicity, sex, and age in these associations. While we hypothesised that the primary direction of causality was from education attainment to physical activity outcomes, physical activity may affect education attainment at younger ages.[39] Further longitudinal analyses may provide stronger evidence on the causal nature of the observed associations and additionally identify the mediators of the disparities observed.

Implications for practice, and policy

Our findings may have important implications for practice and policy. The inequalities in LTPA observed—across both light and moderate-vigorous activity—suggests that policies are required to reduce these inequalities given the multiple anticipated effects on health. Population-level or targeted interventions may be used to reduce the sizeable modification across demographic sub-groups. For example, there was a 13% difference in probability of engaging in light LTPA in those aged 60+ compared to 2-3% of those aged 20-39, suggesting older adults with lower levels of education would benefit most from interventions regarding this domain of physical activity. Furthermore, in line with previous evidence [19] we also found that those of lower educational attainment were more likely to possess physically demanding occupations. This difference is important, if the health consequences of occupational physical activity are less favourable or detrimental compared with LTPA. [5 6] Efforts to increase leisure time activity and its inequality should consider co-occurring differences in occupational activity. Finally, lower engagement in domain-specific physical activity were also found in those with high levels of educational attainment. For example, there was a 10% difference in active travel among high to low educated women compared with 3% in men. Previous evidence has found similar sex differences in cycling to work; however, similar rates of men and women for leisure-time cycling. [40] Suggested means of increasing active

For peer review only - http://bmjopen.bmj.com/site/about/guidelines.xhtml

travel include the provision of safe walking and cycling travel routes, accessible bike locks, and changing facilities.

Finally, our findings may have implications for future studies which investigate inequalities in physical activity outcomes. Existing studies typically adjust for the sociodemographic factors we investigated as potential modifiers. Given the evidence for modification that we found, such analyses may provide biased estimates of the magnitude of inequalities that operate in particular population sub-groups

Conclusions

In summary, we found sex, age, and ethnicity modified associations between educational attainment and multiple physical activity outcomes. Our findings imply there may be unequal access or additional barriers to physical activity across both education and demographic sub groups. Better understanding the characteristics of physically inactive sub-groups may aid development of tailored interventions to increase activity levels and reduce health inequalities.

For peer review only

Table 1. Physical activity domains by education and demographic characteristics in Understanding Society (2013-2015)

Demographic	Physical activity domain															
	Active travel				Occupational				Leisure-time							
									Moderate-to-vigorous				Light			
	Non-active		Active		Not physical		Physical		< Weekly		≥ 1x weekly		< Weekly		≥ 1x weekly	
	N	*%*	*N*	*%*	*N*	*%*	*N*	*%*	*N*	*%*	*N*	*%*	*N*	*%*	*N*	*%*
**Educational attainment**																
GCSEs and lower	3639	26.3	846	6.11	1471	10.6	3920	28.31	11048	79.79	2798	20.21	11482	82.93	2364	17.07
School diploma	4547	36.6	906	7.29	2200	17.7	4383	35.27	8857	71.27	3570	28.73	9910	79.75	2517	20.25
Degree/higher	7519	48.7	1078	6.99	5394	35	5063	32.82	9358	60.66	6069	39.34	11914	77.23	3513	22.77
Missing	27	17.7	6	3.92	11	7.19	28	18.3	126	82.35	27	17.65	137	89.54	16	10.46
P value				<0.001				<0.001				<0.001				<0.001
**Ethnicity**																
White	13397	38.8	2396	6.93	8001	23.1	11199	32.39	23749	68.7	10822	31.3	26892	77.79	7679	22.21
Black	631	43.6	101	6.97	227	15.7	594	40.99	1047	72.26	402	27.74	1308	90.27	141	9.73
Asian	1223	35.6	234	6.81	598	17.4	1158	33.71	2571	74.85	864	25.15	3030	88.21	405	11.79
Other ethnicity	366	40.3	82	9.02	202	22.2	338	37.18	609	67	300	33	764	84.05	145	15.95
Missing	115	7.72	23	1.54	48	3.22	105	7.05	1413	94.9	76	5.1	1449	97.31	40	2.69
P value				<0.001				<0.001				<0.001				<0.001
**Sex**																
Male	7076	36.7	1215	6.3	4296	22.3	6376	33.06	13305	69	5979	31	15741	81.63	3543	18.37
Female	8656	38.4	1621	7.18	4780	21.2	7018	31.1	16084	71.27	6485	28.73	17702	78.44	4867	21.56
P value				<0.001				<0.001				<0.001				<0.001
**Age**																
20-29 years	2703	44.2	633	10.34	1235	20.2	2381	38.91	3848	62.88	2272	37.12	5188	84.77	932	15.23
30-39 years	3650	53.7	636	9.35	2171	31.9	2841	41.79	4220	62.07	2579	37.93	5704	83.89	1095	16.11
40-49 years	4511	54	718	8.59	2730	32.7	3622	43.32	5526	66.09	2835	33.91	6679	79.88	1682	20.12
50-59 years	3594	47.8	640	8.51	2108	28	3137	41.7	5479	72.84	2043	27.16	5855	77.84	1667	22.16
60+ years	1274	9.76	209	1.6	832	6.37	1413	10.83	10316	79.04	2735	20.96	10017	76.75	3034	23.25
P value				<0.001				<0.001				<0.001				<0.001

Participants from wave 5 (2013-2015) of Understanding Society with data on educational attainment, demographics, and physical activity
P value = chi^{2}, Missing = missing data; where not shown there were no missing data.

Table 2. Mutually adjusted associations of educational attainment and demographics with domain specific physical activity outcomes

	Physical Activity															
	Active travel				Occupational				Leisure time							
									Moderate-to-vigorous				Light			
	N=18,404				*N=22,287*				*N= 40,270*				*N= 40,270*			
	OR	*CI*		*p*	*OR*	*CI*		*p*	*OR*	*CI*		*p*	*OR*	*CI*		*p*
**Education**																
GCSEs and lower	**ref*															
School diploma	0.83	0.74	0.93	0.001	0.73	0.67	0.79	<0.001	1.44	1.35	1.53	<0.001	1.33	1.24	1.42	<0.001
Degree/higher	0.59	0.53	0.66	<0.001	0.34	0.31	0.37	<0.001	2.28	2.16	2.42	<0.001	1.58	1.49	1.69	<0.001
**Ethnicity**																
White	**ref*															
Black	0.94	0.74	1.19	0.620	2.19	1.84	2.59	<0.001	0.69	0.61	0.77	<0.001	0.38	0.31	0.46	<0.001
Asian	1.10	0.95	1.29	0.211	1.56	1.38	1.75	<0.001	0.59	0.54	0.64	<0.001	0.51	0.46	0.57	<0.001
Other ethnicity	1.31	1.01	1.69	0.041	1.40	1.16	1.70	0.001	0.82	0.71	0.95	0.008	0.69	0.57	0.83	<0.001
**Sex**																
Male	**ref*															
Female	1.11	1.02	1.21	0.016	1.04	0.99	1.10	0.150	0.84	0.81	0.88	<0.001	1.20	1.14	1.26	<0.001
**Age**																
20-29 years	**ref*															
30-39 years	0.77	0.67	0.88	<0.001	0.71	0.65	0.79	<0.001	0.96	0.88	1.04	0.287	1.02	0.92	1.13	0.676
40-49 years	0.67	0.59	0.76	<0.001	0.68	0.62	0.75	<0.001	0.81	0.75	0.88	<0.001	1.36	1.24	1.50	<0.001
50-59 years	0.74	0.64	0.84	<0.001	0.74	0.68	0.82	<0.001	0.59	0.55	0.64	<0.001	1.52	1.39	1.67	<0.001
60+ years	0.66	0.55	0.79	<0.001	0.83	0.74	0.94	0.003	0.44	0.41	0.48	<0.001	1.64	1.50	1.80	<0.001

Participants from wave 5 (2013-2015) of Understanding Society with valid data on educational attainment, demographics, and physical activity
Samples were restricted to those with valid demographic and physical activity data
Active travel to work: non-active/active; Occupational: non-physical/physical; Leisure-time: <weekly/ ≥ 1x weekly
Analyses are mutually adjusted for education, age, sex, and ethnicity
**reference group*

References

1. Warburton DE, Nicol CW, Bredin SS. Health benefits of physical activity: the evidence. *CMAJ* 2006;174(6):801-9. doi: 10.1503/cmaj.051351
2. Kohl HW, 3rd, Craig CL, Lambert EV, et al. The pandemic of physical inactivity: global action for public health. *Lancet* 2012;380(9838):294-305. doi: 10.1016/S0140-6736(12)60898-8
3. Ding D, Lawson KD, Kolbe-Alexander TL, et al. The economic burden of physical inactivity: a global analysis of major non-communicable diseases. *Lancet* 2016;388(10051):1311-24. doi: 10.1016/S0140-6736(16)30383-X
4. Scholes S, Bann D. Education-related disparities in reported physical activity during leisure-time, active transportation, and work among US adults: repeated cross-sectional analysis from the National Health and Nutrition Examination Surveys, 2007 to 2016. *Bmc Public Health* 2018;18(1):926. doi: 10.1186/s12889-018-5857-z
5. Holtermann A, Hansen JV, Burr H, et al. The health paradox of occupational and leisure-time physical activity. *Br J Sports Med* 2012;46(4):291-5. doi: 10.1136/bjsm.2010.079582 [published Online First: 2011/04/05]
6. Holtermann A, Krause N, van der Beek AJ, et al. The physical activity paradox: six reasons why occupational physical activity (OPA) does not confer the cardiovascular health benefits that leisure time physical activity does. *Br J Sports Med* 2018;52(3):149-50. doi: 10.1136/bjsports-2017-097965 [published Online First: 2017/08/12]
7. Elhakeem A, Cooper R, Bann D, et al. Childhood socioeconomic position and adult leisure-time physical activity: a systematic review. *Int J Behav Nutr Phys Act* 2015;12:92. doi: 10.1186/s12966-015-0250-0
8. Bleich SN, Jarlenski MP, Bell CN, et al. Health inequalities: trends, progress, and policy. *Annu Rev Public Health* 2012;33:7-40. doi: 10.1146/annurev-publhealth-031811-124658
9. Droomers M, Schrijvers CT, Mackenbach JP. Educational level and decreases in leisure time physical activity: predictors from the longitudinal GLOBE study. *J Epidemiol Community Health* 2001;55(8):562-8. [published Online First: 2001/07/13]
10. Harper S, Lynch J. Trends in socioeconomic inequalities in adult health behaviors among US states, 1990-2004. *Public Health Rep* 2007;122(2):177-89. doi: Doi 10.1177/003335490712200207
11. Bann D, Johnson W, Li L, et al. Socioeconomic inequalities in childhood and adolescent body-mass index, weight, and height from 1953 to 2015: an analysis of four longitudinal, observational, British birth cohort studies. *Lancet Public Health* 2018;3(4):E194-E203. doi: 10.1016/S2468-2667(18)30045-8
12. Hayes L, White M, Unwin N, et al. Patterns of physical activity and relationship with risk markers for cardiovascular disease and diabetes in Indian, Pakistani, Bangladeshi and European adults in a UK population. *J Public Health Med* 2002;24(3):170-8.
13. Obesity and ethnicity. In: Health SfP, ed.: National Obesity Observatory, 2011.
14. Fischbacher CM, Hunt S, Alexander L. How physically active are South Asians in the United Kingdom? A literature review. *J Public Health (Oxf)* 2004;26(3):250-8. doi: 10.1093/pubmed/fdh158
15. Telama R. Tracking of physical activity from childhood to adulthood: a review. *Obes Facts* 2009;2(3):187-95. doi: 10.1159/000222244
16. Bécares L. The Ethnic Density effect on the Health of Ethnic Minority People in the United Kingdom: a Study of Hypothesised Pathways. London: University College London, 2009.
17. Pickett KE, Shaw RJ, Atkin K, et al. Ethnic density effects on maternal and infant health in the Millennium Cohort Study. *Soc Sci Med* 2009;69(10):1476-83. doi: 10.1016/j.socscimed.2009.08.031

For peer review only

18. Saffer H, Dave D, Grossman M, et al. Racial, Ethnic, and Gender Differences in Physical Activity. *J Hum Cap* 2013;7(4):378-410. doi: 10.1086/671200
19. He XZ, Baker DW. Differences in leisure-time, household, and work-related physical activity by race, ethnicity, and education. *J Gen Intern Med* 2005;20(3):259-66. doi: 10.1111/j.1525-1497.2005.40198.x
20. Evandrou M, Falkingham J, Feng ZX, et al. Ethnic inequalities in limiting health and self-reported health in later life revisited. *J Epidemiol Commun H* 2016;70(7):653-62. doi: 10.1136/jech-2015-206074
21. Farrell L, Hollingsworth B, Propper C, et al. The socioeconomic gradient in physical inactivity: Evidence from one million adults in England. *Soc Sci Med* 2014;123:55-63. doi: 10.1016/j.socscimed.2014.10.039
22. Stalsberg R, Pedersen AV. Effects of socioeconomic status on the physical activity in adolescents: a systematic review of the evidence. *Scand J Med Sci Sports* 2010;20(3):368-83. doi: 10.1111/j.1600-0838.2009.01047.x [published Online First: 2010/02/09]
23. Berger U, Der G, Mutrie N, et al. The impact of retirement on physical activity. *Ageing Soc* 2005;25:181-95. doi: 10.1017/S0144686x04002739
24. Mutrie N, Hannah MK. Some work hard while others play hard. *International Journal of Health Promotion and Education* 2013;42(2):109-17. doi: 10.1080/14635240.2004.10708024
25. Stringhini S, Sabia S, Shipley M, et al. Association of socioeconomic position with health behaviors and mortality. *JAMA* 2010;303(12):1159-66. doi: 10.1001/jama.2010.297 [published Online First: 2010/03/25]
26. Knies G. Unverstanding Society: The UK Household Longitundal Study: Waves 1-8 User Guide. Essex: University of Essex, 2018.
27. Department for Digital C, Media & Sport. Taking Part Survey 2016 [Available from: https://www.gov.uk/guidance/taking-part-survey#the-taking-part-survey.
28. Ainsworth BE, Haskell WL, Herrmann SD, et al. 2011 Compendium of Physical Activities: a second update of codes and MET values. *Med Sci Sports Exerc* 2011;43(8):1575-81. doi: 10.1249/MSS.0b013e31821ece12 [published Online First: 2011/06/18]
29. Bauman AE, Reis RS, Sallis JF, et al. Correlates of physical activity: why are some people physically active and others not? *Lancet* 2012;380(9838):258-71. doi: 10.1016/S0140-6736(12)60735-1 [published Online First: 2012/07/24]
30. . In: CABE, ed. Community green: using local spaces to tackle inequality and improve health. London.
31. Rojas A, Storch EA. Psychological complications of obesity. *Pediatr Ann* 2010;39(3):174-80. doi: 10.3928/00904481-20100223-07
32. Panter JR, Jones AP, van Sluijs EM. Environmental determinants of active travel in youth: a review and framework for future research. *Int J Behav Nutr Phys Act* 2008;5:34. doi: 10.1186/1479-5868-5-34 [published Online First: 2008/06/25]
33. Telama R, Yang X, Viikari J, et al. Physical activity from childhood to adulthood: a 21-year tracking study. *Am J Prev Med* 2005;28(3):267-73. doi: 10.1016/j.amepre.2004.12.003
34. Hooker SP, Wilson DK, Griffin SF, et al. Perceptions of environmental supports for physical activity in African American and white adults in a rural county in South Carolina. *Prev Chronic Dis* 2005;2(4):A11. [published Online First: 2005/09/17]
35. Caspersen CJ, Pereira MA, Curran KM. Changes in physical activity patterns in the United States, by sex and cross-sectional age. *Med Sci Sports Exerc* 2000;32(9):1601-9. [published Online First: 2000/09/20]
36. Mann CJ. Observational research methods. Research design II: cohort, cross sectional, and case-control studies. *Emerg Med J* 2003;20(1):54-60. [published Online First: 2003/01/21]
37. Watkinson C, van Sluijs EM, Sutton S, et al. Overestimation of physical activity level is associated with lower BMI: a cross-sectional analysis. *Int J Behav Nutr Phys Act* 2010;7:68. doi: 10.1186/1479-5868-7-68 [published Online First: 2010/09/22]

For peer review only

38. Kapteyn A, Banks J, Hamer M, et al. What they say and what they do: comparing physical activity across the USA, England and the Netherlands. *J Epidemiol Community Health* 2018;72(6):471-76. doi: 10.1136/jech-2017-209703 [published Online First: 2018/04/13]
39. . In: Kohl HW, III, Cook HD, eds. Educating the Student Body: Taking Physical Activity and Physical Education to School. Washington (DC)2013.
40. Heesch KC, Sahlqvist S, Garrard J. Gender differences in recreational and transport cycling: a cross-sectional mixed-methods comparison of cycling patterns, motivators, and constraints. *Int J Behav Nutr Phys Act* 2012;9:106. doi: 10.1186/1479-5868-9-106 [published Online First: 2012/09/11]

For peer review only

Funding

This project is part of a collaborative research programme entitled 'Cohorts and Longitudinal Studies Enhancement Resources' (CLOSER) funded by the ESRC (http://www.esrc.ac.uk) (ES/K000357/1). DB and MF are also supported by the Academy of Medical Sciences/the Wellcome Trust "Springboard Health of the public in 2040" Award (HOP001\1025). SPP is funded by a UK Medical Research Council Career Development Award (MR/P020372/1). MB is funded by ESRC ES/N00812X/1. Understanding Society is funded by the Economic and Social Research Council (ES/N00812X/1).

Competing interests

None to declare.

Author contributions

MF, DB, SPP and RC were involved in the conception and design of the study, MF conducted the analyses and drafted the manuscript, and MF, SPP, MB, MH, BJ, LG, RC and DB have revised the manuscript and approved for publication.

Data availability statement

Data for this study, Understanding Society, was accessed through the UK Data Service (https://www.ukdataservice.ac.uk).

Licence statement

I, the Submitting Author has the right to grant and does grant on behalf of all authors of the Work (as defined in the below author licence), an exclusive licence and/or a non-exclusive licence for contributions from authors who are: i) UK Crown employees; ii) where BMJ has agreed a CC-BY licence shall apply, and/or iii) in accordance with the terms applicable for US Federal Government officers or employees acting as part of their official duties; on a worldwide, perpetual, irrevocable, royalty-free basis to BMJ Publishing Group Ltd ("BMJ") its licensees and where the relevant Journal is co-owned by BMJ to the co-owners of the Journal, to publish the Work in BMJ Open and any other BMJ products and to exploit all rights, as set out in our licence.

The Submitting Author accepts and understands that any supply made under these terms is made by BMJ to the Submitting Author unless you are acting as an employee on behalf of your employer or a postgraduate student of an affiliated institution which is paying any applicable article publishing charge ("APC") for Open Access articles. Where the Submitting Author wishes to make the Work available on an Open Access basis (and intends to pay the relevant APC), the

For peer review only - http://bmjopen.bmj.com/site/about/guidelines.xhtml

terms of reuse of such Open Access shall be governed by a Creative Commons licence – details of these licences and which Creative Commons licence will apply to this Work are set out in our licence referred to above.

For peer review only

For peer review only - http://bmjopen.bmj.com/site/about/guidelines.xhtml

Figure 1. Educational differences in active travel by sex, ethnicity, and age

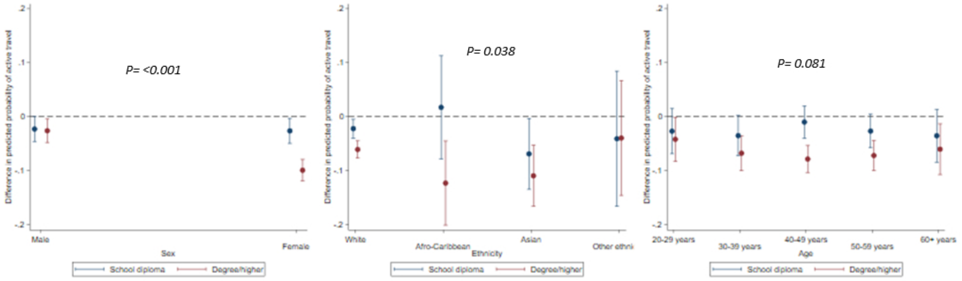

Figure 2. Educational differences in occupational physical activity by sex, ethnicity, and age

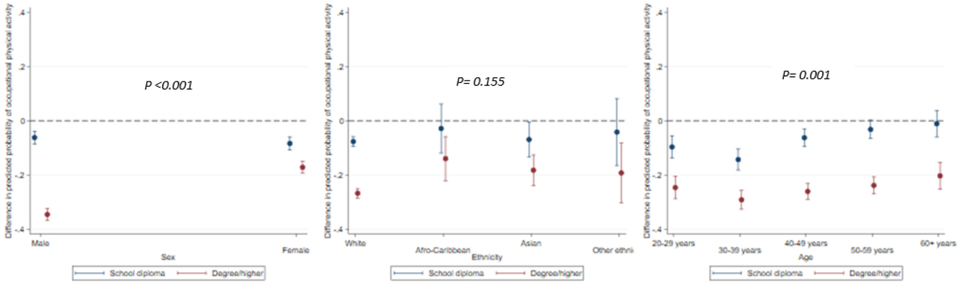

Data: Understanding Society, Wave 5. Dotted line represents reference group (GCSE & lower). P values indicate demographic x education

Figure 3. Educational differences in moderate to vigorous intesity activities by sex, ethnicity, and age

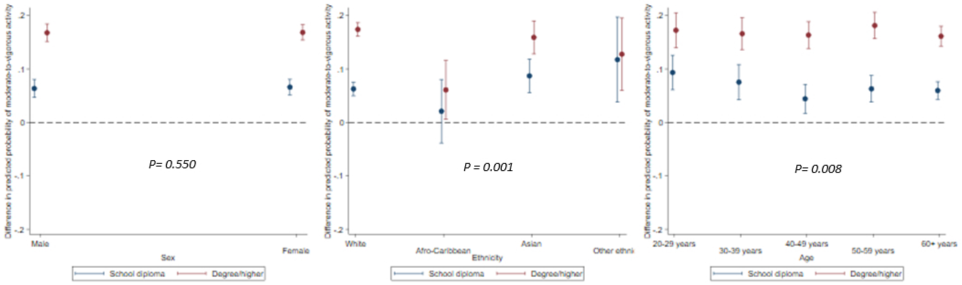

Figure 4. Educational differences in light intesity activities by sex, ethnicity, and age

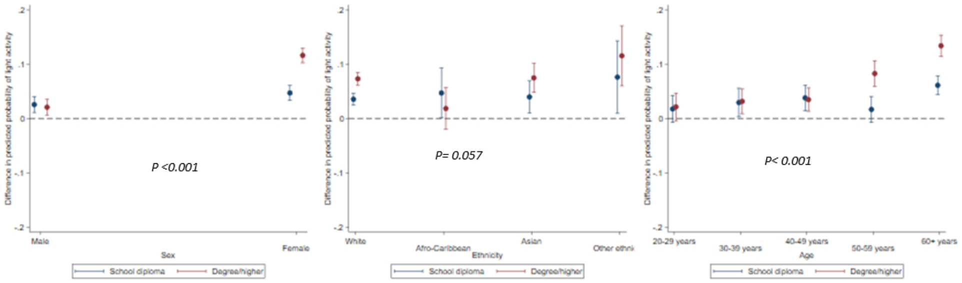

Data: Understanding Society, Wave 5. Dotted line represents reference group (GCSE & lower). P values indicate demographic x education)

Table S1. Ethnicity, sex and age by highest educational attainment in Understanding Society (2013-2015)

**Demographics**	Highest educational attainment					
	GCSEs and lower		School diploma & other qualifications		Degree/higher	
	N	*(%)*	N	*(%)*	N	*(%)*
**Ethnicity**						
White	11638	33.7	10424	30.2	12424	35.9
Black	363	25.1	400	27.6	685	47.3
Asian	1106	32.2	877	25.5	1445	42.1
Other ethnicity	201	22.1	235	25.9	472	51.9
Missing	538	36.1	491	33	401	26.9
P value	<0.001					
**Sex**						
Male	5888	30.5	6388	33.1	6927	35.9
Female	7958	35.3	6039	26.8	8500	37.7
P value	<0.001					
**Age**						
20-29 years	1462	23.9	2363	38.6	2271	37.1
30-39 years	1653	24.3	1776	26.1	3345	49.2
40-49 years	2433	29.1	2339	28	3570	42.7
50-59 years	2455	32.6	2221	29.5	2822	37.5
60+	5843	44.8	3728	28.6	3419	26.2
P value	<0.001					

Participants from wave 5 (2013-2015) of Understanding Society with data on educational attainment, demographics, and physical activity
P value = chi^{2}, Missing = missing data; where not shown there were no missing data

For peer review only

Table S2. Estimated difference in predicted probability in educational differences in physical activity by age

**Physical activity**	**20-29**				**30-39**				**40-49**				**50-59**				**60+**				SEP X age
	Coef.	*91% CI*		*p*	*Coef.*	*91% CI*		*p*	*Coef.*	*91% CI*		*p*	*Coef.*	*91% CI*		*p*	*Coef.*	*91% CI*		*p*	
**Active Travel**																					
GCSEs and lower																					
School diploma	-0.03	-0.07	0.01	0.206	-0.04	-0.07	0.00	0.064	-0.01	-0.04	0.02	0.506	-0.03	-0.06	0.00	0.091	-0.04	-0.08	0.01	0.155	0.081
Degree/higher	-0.04	-0.08	0.00	0.044	-0.07	-0.10	-0.04	<0.001	-0.08	-0.10	-0.05	<0.001	-0.07	-0.10	-0.04	<0.001	-0.06	-0.11	-0.01	0.012	
**Occupational**																					
GCSEs and lower																					
School diploma	-0.10	-0.14	-0.06	<0.001	-0.14	-0.18	-0.10	<0.001	-0.06	-0.10	-0.03	<0.001	-0.03	-0.07	0.00	0.060	-0.01	-0.06	0.04	0.648	0.001
Degree/higher	-0.25	-0.29	-0.21	<0.001	-0.29	-0.33	-0.26	<0.001	-0.26	-0.29	-0.23	<0.001	-0.24	-0.27	-0.21	<0.001	-0.20	-0.25	-0.15	<0.001	
**Moderate-to-vigorous leisure time**																					
GCSEs and lower																					
School diploma	0.09	0.06	0.13	<0.001	0.08	0.04	0.11	<0.001	0.04	0.02	0.07	0.001	0.06	0.04	0.09	<0.001	0.06	0.04	0.08	<0.001	0.008
Degree/higher	0.17	0.14	0.21	<0.001	0.17	0.14	0.20	<0.001	0.16	0.14	0.19	<0.001	0.18	0.16	0.21	<0.001	0.16	0.14	0.18	<0.001	
**Light leisure time**																					
GCSEs and lower																					
School diploma	0.02	-0.01	0.04	0.173	0.03	0.00	0.06	0.030	0.04	0.01	0.06	0.002	0.02	-0.01	0.04	0.180	0.06	0.04	0.08	<0.001	<0.001
Degree/higher	0.02	0.00	0.05	0.102	0.03	0.01	0.05	0.007	0.03	0.01	0.06	0.002	0.08	0.06	0.11	<0.001	0.13	0.11	0.15	<0.001	

Estimates are derived from separate logistic regression models of each binary physical activity outcome, and show the education-outcome associations on the absolute scale in each sociodemographic sub group

Participants from wave 5 (2013-2015) of Understanding Society with data on educational attainment, demographics, and physical activity

**reference groups*

Table S3. Estimated difference in predicted probability in educational differences in physical activity by sex

Physical activity	Male				Female				SEP X sex
	Coef.	*91% CI*		*p*	*Coef.*	*91% CI*		*p*	
**Active Travel**									
GCSEs and lower	**ref*								
School diploma	-0.02	-0.05	0.00	0.050	-0.03	-0.05	0.00	0.027	<0.001
Degree/higher	-0.03	-0.05	0.00	0.018	-0.10	-0.12	-0.08	0.000	
**Occupational**									
GCSEs and lower									
School diploma	-0.06	-0.09	-0.04	<0.001	-0.08	-0.11	-0.06	<0.001	<0.001
Degree/higher	-0.35	-0.37	-0.32	<0.001	-0.17	-0.19	-0.15	<0.001	
**Moderate-to-vigorous leisure time**									
GCSEs and lower									
School diploma	0.06	0.05	0.08	<0.001	0.07	0.05	0.08	<0.001	0.550
Degree/higher	0.17	0.15	0.18	<0.001	0.17	0.15	0.18	<0.001	
**Light leisure time**									
GCSEs and lower									
School diploma	0.03	0.01	0.04	0.001	0.05	0.03	0.06	<0.001	<0.001
Degree/higher	0.02	0.01	0.03	0.005	0.12	0.10	0.13	<0.001	

Estimates are derived from separate logistic regression models of each binary physical activity outcome, and show the education-outcome associations on the absolute scale in each sociodemographic sub group

Participants from wave 5 (2013-2015) of Understanding Society with data on educational attainment, demographics, and physical activity

**reference group*

For peer review only - http://bmjopen.bmj.com/site/about/guidelines.xhtml

Table S4. Estimated difference in predicted probability in educational differences in physical activity by ethnicity

**Physical activity**	**White**				**Black**				**Asian**				**Other ethnicity**				SEP X ethnicity
	Coef.	*91% CI*		*p*	*Coef.*	*91% CI*		*p*	*Coef.*	*91% CI*		*p*	*Coef.*	*91% CI*		*p*	P
**Active Travel**																	
GCSEs and lower																	
School diploma	-0.02	-0.04	0.00	0.013	0.02	-0.08	0.11	0.723	-0.07	-0.13	0.00	0.040	-0.04	-0.17	0.08	0.523	0.038
Degree/higher	-0.06	-0.08	-0.04	<0.001	-0.12	-0.20	-0.05	0.002	-0.11	-0.17	-0.05	<0.001	-0.04	-0.15	0.07	0.465	
**Occupational**																	
GCSEs and lower																	
School diploma	-0.08	-0.10	-0.06	<0.001	-0.03	-0.12	0.06	0.526	-0.07	-0.13	0.00	0.035	-0.04	-0.17	0.08	0.502	0.155
Degree/higher	-0.27	-0.29	-0.25	<0.001	-0.14	-0.22	-0.06	0.001	-0.18	-0.24	-0.13	<0.001	-0.19	-0.30	-0.08	0.001	
**Moderate-to-vigorous leisure time**																	
GCSEs and lower																	
School diploma	0.06	0.05	0.08	<0.001	0.02	-0.04	0.08	0.488	0.09	0.06	0.12	<0.001	0.12	0.04	0.20	0.004	0.001
Degree/higher	0.17	0.16	0.19	<0.001	0.06	0.01	0.12	0.030	0.16	0.13	0.19	<0.001	0.13	0.06	0.20	<0.001	
**Light leisure time**																	
GCSEs and lower																	
School diploma	0.04	0.02	0.05	<0.001	0.05	0.00	0.09	0.046	0.04	0.01	0.07	0.008	0.08	0.01	0.14	0.026	0.057
Degree/higher	0.07	0.06	0.08	<0.001	0.02	-0.02	0.06	0.351	0.07	0.05	0.10	<0.001	0.12	0.06	0.17	<0.001	

Estimates are derived from separate logistic regression models of each binary physical activity outcome, and show the education-outcome associations on the absolute scale in each sociodemographic sub group
Participants from wave 5 (2013-2015) of Understanding Society with data on educational attainment, demographics, and physical activity
**reference group*

Table S5. Unadjusted associations (OR (95% CI)) of education and demographics with domain specific PA (complete case analysis)

	Physical Activity															
	Active travel				Occupational				Leisure							
									Moderate-to-vigorous				Light			
	N=18,404				*N=22,287*				*N= 40,270*				*N= 40,270*			
	OR	*CI*		*p*	*OR*	*CI*		*p*	*OR*	*CI*		*p*	*OR*	*CI*		*p*
**Education**																
GCSEs and lower	**ref*															
School diploma	0.86	0.77	0.96	0.006	0.75	0.69	0.81	<0.001	1.59	1.50	1.69	<0.001	1.23	1.16	1.31	<0.001
Degree/higher	0.62	0.55	0.68	<0.001	0.35	0.33	0.38	<0.001	2.55	2.41	2.69	<0.001	1.41	1.33	1.50	<0.001
**Ethnicity**																
White	**ref*															
Black	0.89	0.71	1.13	0.358	1.87	1.59	2.20	<0.001	0.84	0.75	0.95	0.005	0.38	0.31	0.45	<0.001
Asian	1.07	0.92	1.25	0.388	1.38	1.23	1.55	<0.001	0.74	0.68	0.81	<0.001	0.47	0.42	0.52	<0.001
Other ethnicity	1.25	0.97	1.61	0.080	1.20	0.99	1.44	0.061	1.08	0.94	1.25	0.277	0.66	0.55	0.80	<0.001
**Sex**																
Male	**ref*															
Female	1.09	1.01	1.19	0.033	0.99	0.94	1.05	0.822	0.85	0.82	0.89	<0.001	1.17	1.12	1.23	<0.001
**Age**																
20-29 years	**ref*															
30-39 years	0.75	0.65	0.85	<0.001	0.68	0.62	0.75	<0.001	1.01	0.93	1.09	0.875	1.05	0.95	1.16	0.353
40-49 years	0.68	0.60	0.77	<0.001	0.69	0.63	0.76	<0.001	0.84	0.78	0.90	<0.001	1.38	1.25	1.51	<0.001
50-59 years	0.76	0.67	0.87	<0.001	0.78	0.71	0.85	<0.001	0.60	0.56	0.65	<0.001	1.56	1.42	1.71	<0.001
60+ years	0.70	0.58	0.83	<0.001	0.88	0.79	0.99	0.034	0.43	0.39	0.46	<0.001	1.64	1.50	1.80	<0.001

Participants from wave 5 (2013-2015) of Understanding Society with data on educational attainment, demographics, and physical activity
Samples were restricted to those with valid demographic and physical activity data
*Active travel to work: non-active/active; Occupational: non-physical/physical; Leisure-time: <weekly/ ≥ 1x weekly*reference groups*

Table S6. Unadjusted associations (OR (95% CI)) of education and demographics with domain specific PA (maximum available samples)

	Physical Activity																			
	Active travel					Occupational					Leisure									
											Moderate-to-vigorous					Light				
	N	*OR*	*CI*		*p*	N	*OR*	*CI*		*p*	N	*OR*	*CI*		*p*	N	*OR*	*CI*		*p*
**Education**																				
GCSEs and lower	18535	**ref*				22431					41700					41700				
School diploma		0.86	0.77	0.96	0.006		0.75	0.69	0.81	<0.001		1.59	1.50	1.69	<0.001		1.23	1.16	1.31	<0.001
Degree/higher		0.62	0.55	0.69	<0.001		0.35	0.33	0.38	<0.001		2.56	2.43	2.70	<0.001		1.43	1.35	1.52	<0.001
**Ethnicity**																				
White	18430	**ref*				22317					40364					40364				
Black		0.89	0.71	1.13	0.360		1.87	1.59	2.20	<0.001		0.84	0.75	0.95	0.005		0.38	0.31	0.45	<0.001
Asian		1.07	0.92	1.25	0.385		1.38	1.23	1.55	<0.001		0.74	0.68	0.81	<0.001		0.47	0.42	0.52	<0.001
Other ethnicity		1.25	0.97	1.61	0.080		1.20	0.99	1.44	0.062		1.08	0.94	1.25	0.282		0.66	0.55	0.80	<0.001
**Sex**																				
Male	18568	**ref*				22470					41853					41853				
Female		1.09	1.00	1.18	0.039		0.99	0.94	1.04	0.688		0.90	0.86	0.93	<0.001		1.22	1.17	1.28	<0.001
**Age**																				
20-29 years	18568	**ref*				22470					41853					41853				
30-39 years		0.74	0.65	0.85	<0.001		0.68	0.62	0.75	<0.001		1.04	0.96	1.12	0.380		1.07	0.97	1.18	0.192
40-49 years		0.68	0.60	0.77	<0.001		0.69	0.63	0.75	<0.001		0.87	0.81	0.94	<0.001		1.40	1.28	1.54	<0.001
50-59 years		0.76	0.67	0.87	<0.001		0.77	0.71	0.84	<0.001		0.63	0.59	0.68	<0.001		1.58	1.45	1.74	<0.001
60+ years		0.70	0.59	0.84	<0.001		0.88	0.79	0.99	0.031		0.45	0.42	0.48	<0.001		1.69	1.54	1.84	<0.001

Participants from wave 5 (2013-2015) of Understanding Society with data on educational attainment, demographics, and physical activity
Sample size was not restricted and represents total available sample
*Active travel to work: non-active/active; Occupational: non-physical/physical; Leisure-time: <weekly/ ≥ 1x weekly*reference groups*

Figure S1. Flow diagram of analyses in Understanding Society

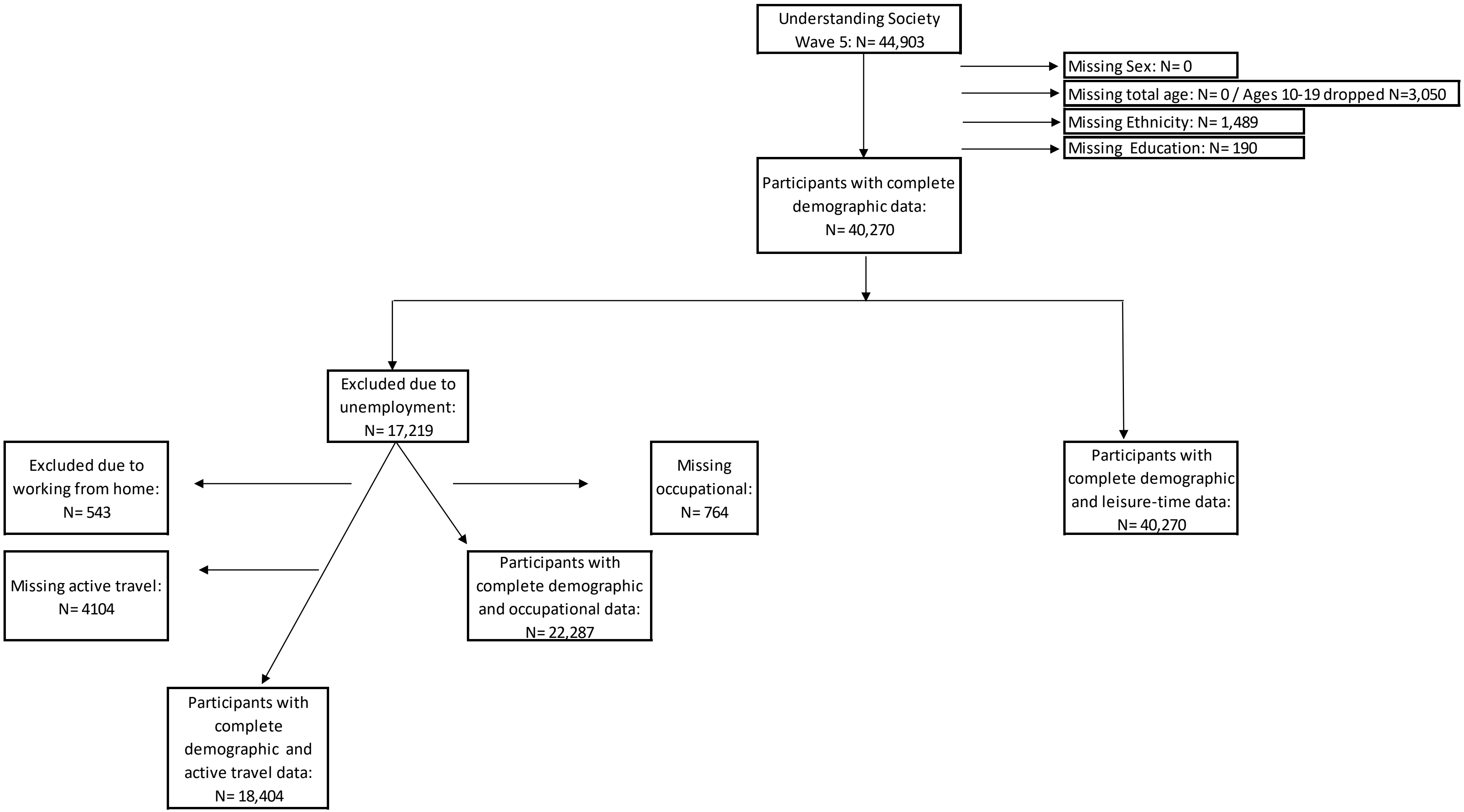

Reporting checklist for cross sectional study.

Based on the STROBE cross sectional guidelines.

Instructions to authors

Complete this checklist by entering the page numbers from your manuscript where readers will find each of the items listed below.

Your article may not currently address all the items on the checklist. Please modify your text to include the missing information. If you are certain that an item does not apply, please write "n/a" and provide a short explanation.

Upload your completed checklist as an extra file when you submit to a journal.

In your methods section, say that you used the STROBE cross sectionalreporting guidelines, and cite them as:

von Elm E, Altman DG, Egger M, Pocock SJ, Gotzsche PC, Vandenbroucke JP. The Strengthening the Reporting of Observational Studies in Epidemiology (STROBE) Statement: guidelines for reporting observational studies.

		Reporting Item	Page Number
**Title and abstract**			
Title	#1a	Indicate the study's design with a commonly used term in the title or the abstract	1
Abstract	#1b	Provide in the abstract an informative and balanced summary of what was done and what was found	2
**Introduction**			
Background / rationale	#2	Explain the scientific background and rationale for the investigation being reported	4
Objectives	#3	State specific objectives, including any prespecified hypotheses	5
**Methods**			

For peer review only

Study design	#4	Present key elements of study design early in the paper	6/7
Setting	#5	Describe the setting, locations, and relevant dates, including periods of recruitment, exposure, follow-up, and data collection	6
Eligibility criteria	#6a	Give the eligibility criteria, and the sources and methods of selection of participants.	6/7
	#7	Clearly define all outcomes, exposures, predictors, potential confounders, and effect modifiers. Give diagnostic criteria, if applicable	6/7
Data sources / measurement	#8	For each variable of interest give sources of data and details of methods of assessment (measurement). Describe comparability of assessment methods if there is more than one group. Give information separately for for exposed and unexposed groups if applicable.	6/7
Bias	#9	Describe any efforts to address potential sources of bias	7
Study size	#10	Explain how the study size was arrived at	7
Quantitative variables	#11	Explain how quantitative variables were handled in the analyses. If applicable, describe which groupings were chosen, and why	6/7
Statistical methods	#12a	Describe all statistical methods, including those used to control for confounding	7
Statistical methods	#12b	Describe any methods used to examine subgroups and interactions	7
Statistical methods	#12c	Explain how missing data were addressed	7
Statistical methods	#12d	If applicable, describe analytical methods taking account of sampling strategy	6/7
Statistical methods	#12e	Describe any sensitivity analyses	n/a

Results

For peer review only

Participants	#13a	Report numbers of individuals at each stage of study—eg numbers potentially eligible, examined for eligibility, confirmed eligible, included in the study, completing follow-up, and analysed. Give information separately for for exposed and unexposed groups if applicable.	8, 13, 21, 27
Participants	#13b	Give reasons for non-participation at each stage	27
Participants	#13c	Consider use of a flow diagram	27
Descriptive data	#14a	Give characteristics of study participants (eg demographic, clinical, social) and information on exposures and potential confounders. Give information separately for exposed and unexposed groups if applicable.	8, 21
Descriptive data	#14b	Indicate number of participants with missing data for each variable of interest	8, 13, 21
Outcome data	#15	Report numbers of outcome events or summary measures. Give information separately for exposed and unexposed groups if applicable.	8, 13, 21
Main results	#16a	Give unadjusted estimates and, if applicable, confounder-adjusted estimates and their precision (eg, 95% confidence interval). Make clear which confounders were adjusted for and why they were included	14, 22, 23
Main results	#16b	Report category boundaries when continuous variables were categorized	n/a
Main results	#16c	If relevant, consider translating estimates of relative risk into absolute risk for a meaningful time period	n/a
Other analyses	#17	Report other analyses done—e.g., analyses of subgroups and interactions, and sensitivity analyses	n/a
**Discussion**			
Key results	#18	Summarise key results with reference to study objectives	10
Limitations	#19	Discuss limitations of the study, taking into account sources of potential bias or imprecision. Discuss both direction and magnitude of any potential bias.	10/11

For peer review only

Interpretation	#20	Give a cautious overall interpretation considering objectives, limitations, multiplicity of analyses, results from similar studies, and other relevant evidence.	11/12
Generalisability	#21	Discuss the generalisability (external validity) of the study results	10
**Other Information**			
Funding	#22	Give the source of funding and the role of the funders for the present study and, if applicable, for the original study on which the present article is based	20

None The STROBE checklist is distributed under the terms of the Creative Commons Attribution License CC-BY. This checklist can be completed online using https://www.goodreports.org/, a tool made by the EQUATOR Network in collaboration with Penelope.ai

For peer review only

BMJ Open

Educational differentials in key domains of physical activity by ethnicity, age, and sex: a cross-sectional study of over 44,000 participants in The UK Household Longitudinal Study (2013-2015)

Journal:	*BMJ Open*
Manuscript ID	bmjopen-2019-033318.R1
Article Type:	Original research
Date Submitted by the Author:	23-Oct-2019
Complete List of Authors:	Fluharty, Meg; University College London, Centre for Longitudinal Studies<br>Pinto Pereira, Snehal; University College London Institute of Child Health, UCL Research Department of Epidemiology & Public Health<br>Benzeval, Michaela; University of Essex, Institute for Social and Economic Research<br>Hamer, Mark; University College London, Division of Surgery & Interventional Science<br>Jefferis, Barbara ; University College London, Department of Primary Care and Population Health<br>Griffiths, Lucy; Swansea University Medical School, Health Data Research UK, Wales and Northern Ireland<br>Cooper, Rachel; Manchester Metropolitan University, Sport and Exercise Sciences<br>Bann, David; University College London, Centre for Longitudinal Studies
**Primary Subject Heading**:	Epidemiology
Secondary Subject Heading:	Public health
Keywords:	health disparities, EPIDEMIOLOGY, physical activity

SCHOLARONE™
Manuscripts

I, the Submitting Author has the right to grant and does grant on behalf of all authors of the Work (as defined in the below author licence), an exclusive licence and/or a non-exclusive licence for contributions from authors who are: i) UK Crown employees; ii) where BMJ has agreed a CC-BY licence shall apply, and/or iii) in accordance with the terms applicable for US Federal Government officers or employees acting as part of their official duties; on a worldwide, perpetual, irrevocable, royalty-free basis to BMJ Publishing Group Ltd ("BMJ") its licensees and where the relevant Journal is co-owned by BMJ to the co-owners of the Journal, to publish the Work in this journal and any other BMJ products and to exploit all rights, as set out in our licence.

The Submitting Author accepts and understands that any supply made under these terms is made by BMJ to the Submitting Author unless you are acting as an employee on behalf of your employer or a postgraduate student of an affiliated institution which is paying any applicable article publishing charge ("APC") for Open Access articles. Where the Submitting Author wishes to make the Work available on an Open Access basis (and intends to pay the relevant APC), the terms of reuse of such Open Access shall be governed by a Creative Commons licence – details of these licences and which Creative Commons licence will apply to this Work are set out in our licence referred to above.

Other than as permitted in any relevant BMJ Author's Self Archiving Policies, I confirm this Work has not been accepted for publication elsewhere, is not being considered for publication elsewhere and does not duplicate material already published. I confirm all authors consent to publication of this Work and authorise the granting of this licence.

For peer review only

Running Head: Educational differentials in physical activity

Educational differentials in key domains of physical activity by ethnicity, age, and sex: a cross-sectional study of over 44,000 participants in The UK Household Longitudinal Study (2013-2015)

Meg E Fluharty[1], Snehal M Pinto Pereira[2], Michaela Benzeval[3], Mark Hamer[4], Barbara Jefferis[5], Lucy Griffiths[6], Rachel Cooper[7] David Bann[1]

1. Centre for Longitudinal Studies, Institute of Education, University College London
2. UCL Research Department of Epidemiology & Public Health, University College London
3. Institute for Social and Economic Research, University of Essex
4. Division of Surgery and Interventional Science, University College London
5. Department of Primary Care and Population Health, University College London
6. Health Data Research UK, Wales and Northern Ireland, Swansea University Medical School
7. Musculoskeletal Science and Sports Medicine Research Centre, Department of Sport and Exercise Sciences, Faculty of Science and Engineering, Manchester Metropolitan University

Corresponding author Meg Fluharty, Centre for Longitudinal Studies, University College London, 55-59 Gordon Square, London WC1H 0NU, United Kingdom. Email: m.fluharty@ucl.ac.uk

For peer review only

Abstract

Objectives: To assess whether educational differentials in three key physical activity (PA) domains vary by age, sex and ethnicity

Design: National cross-sectional survey

Setting: UK

Participants: Up to 44,903 participants, aged 20 years and over, from the UK Household Longitudinal Study with information on education, PA and demographics collected in 2013-2015.

Primary and secondary outcome measures: Participation in active travel (AT), occupational activity (OA) and leisure time physical activity (LTPA) at the time of assessment

Results: Lower educational attainment was associated with higher AT and OA, but lower weekly LTPA activity; these associations were modified by sex, ethnicity, and age. Education-related differences in AT were larger for females—the difference in predicted probability of activity between highest and lowest education groups was -10% in females (95% CI: -11.9, 7.9) and -3% in males (-4.8, -0.4). Education-related differences in OA were larger among males -35% (-36.9, -32.4) than females -17% (-19.4, -15.0). Finally, education-related differences in moderate-to-vigorous LTPA varied by ethnicity; for example, differences were 17% (16.2, 18.7) for White individuals compared with 6% (0.6, 11.6) for Black individuals.

Conclusions: Educational differences in PA vary by domain, and are modified by age, sex, and ethnicity. A better understanding of physically inactive sub-groups may aid development of interventions to both increase activity levels and reduce health inequalities.

For peer review only

Strengths and limitations of this study:

1. This study includes a large nationally representative sample, enabling us to examine the role of ethnicity, sex and age as modifiers of the relationship between educational attainment and physical activity.

2. This study examined activity outcomes across three domains; previous studies investigating associations of physical activity typically use a single physical activity outcome measure, capturing either 'leisure' or 'unspecified' activity

3. All physical activity measures were captured via self-report, which may be subject to recall bias with individuals either over or under reporting their levels of physical activity

4. Missing data may introduce bias, although missing data due to item missingness (as opposed to specific question gating) was low

5 This study identified cross-sectional associations of education with physical activity across key domains; however, further longitudinal evidence is required to provide stronger evidence of causality and investigate the mediators of the observed associations.

For peer review only

Introduction

Physical activity is an important modifiable determinant of health. [1] Leisure time physical activity's (LTPA) benefits are particularly well-documented and include improvements in the musculoskeletal system, maintenance of healthy weight, protection against cardiovascular disease, and reduction in symptoms of depression and anxiety.[1] However, there is a global trend towards high levels of leisure time physical inactivity which is estimated to contribute to ~6-10% of major non-communicable diseases, ~5.3 million deaths annually, [2] and ~$67.5 billion per year in health care expenditure. [3]

Physical activity can be accrued through multiple domains (e.g. active travel, leisure time, occupation, and domestic/housework), which may have differing impacts on health outcomes. [4] For example, LTPA is thought to be beneficial to physical health and wellbeing, while labour-intense occupations may increase risk of musculoskeletal strain. [5 6] Therefore, examining these different domains may provide evidence to help inform where possible interventions could be targeted. Understanding what is driving differences in activity participation overall, as well as in different domains of physical activity, may also help to identify which forms of activity could be intervened on to reduce socioeconomic disparities in health.

Recent reviews find evidence of socioeconomic disparities in LTPA in high-income countries [7] that have persisted across recent decades. [8] Additionally, lower education has been shown to be associated with higher risk of future declines in LTPA. [9 10] Alongside indicators of socioeconomic position, a number of other sociodemographic factors, including ethnicity, sex, and age have been shown to be associated with physical activity. [11] For example, differences in the levels of PA participation have been reported across ethnic groups in the UK, with those of 'mixed' ethnicity having the highest prevalence of LTPA, [12 13] and South Asians the lowest. [13 14] Numerous factors may explain these differences including personal, socioeconomic, cultural, and environmental factors. [12 13] Alongside ethnic differences, physical activity levels have been found to be lower for women than men and for older than younger adults. [15]

Educational disparities in physical activity may arise though a number of routes including due to differences in knowledge of the health impacts of LTPA, material pathways (such as low income affecting the affordability of activity participation), and potentially due to selection into neighbourhoods which differ in their suitability for outdoor physical activity. [16 17] For example, lower education may lead to lower income and wealth, and thus a greater likelihood of residing in more disadvantaged areas. Educational differences in physical activity participation may also be modified

For peer review only - http://bmjopen.bmj.com/site/about/guidelines.xhtml

by ethnicity, age, and sex. [4 18] For example, manual occupations that men are more likely employed in are usually more physically demanding than equivalent roles undertaken by women; [4 19] this contrasts with the lower participation in leisure-time activities often observed among men with lower levels of education. [20] Moreover, evidence from the US has indicated education-related disparities across multiple activity domains. [4] However, these associations have not yet been investigated within the UK. Previous studies that have investigated associations of different indicators of socioeconomic position, including education, with physical activity outcomes are limited by only investigating one specific domain, [21 22] or use population samples from specific regions within the UK. [23 24] Thus, important gaps remain in our understanding of the nature of socioeconomic inequalities in physical activity outcomes. These are important to fill given their purported mediating role in socioeconomic inequalities in many important health outcomes including premature mortality. [25]

We sought to address the above-mentioned gaps in the literature by investigating educational disparities in physical activity across active travel, leisure, and occupational domains. Additionally, we aimed to examine if associations between education and domain specific physical activity were modified by ethnicity, age, and sex. We hypothesised that lower education status would be associated with lower levels of participation in physical activity during leisure time, but higher participation in active travel and occupational activity and that these associations would be modified by ethnicity, age and sex. A large household panel study was used (Understanding Society), which benefits from national representation, oversampling of ethnic minority groups, and detailed measures of domain-specific physical activity.

For peer review only

For peer review only - http://bmjopen.bmj.com/site/about/guidelines.xhtml

Methods

Participants

Understanding Society: the UK Household Longitudinal Study (UKHLS) is a nationally and regionally representative study which started in 2009 aiming to recruit individuals in 40,000 households.[26] Using a stratified, clustered, equal probability design, UKHLS ensures proper representation from a range of geographical areas, taking into account socioeconomic and ethnic compositions of neighbourhoods. [27] The study annually samples all individuals in the household over the age of 10. Additionally, sample members are followed when they leave the household, and new individuals join the study as they become part of an existing study member's household. Information is collected from participants on a range of information including wellbeing, health, home, family and employment. Detailed study information and sampling methodology can be found elsewhere. [26] Ethnical approval was approved for all waves by the University of Essex Ethnics Committee, and all participants gave written consent for use of their anonymised survey information.

The sample for our analysis includes adult (20 years or over) responders who took part in Wave 5 (2013-2015) and responded to demographic and physical activity questions via computer-assisted personal interview. There was a general population response rate for wave 5 of 70%, although there was a lower response rate among men and younger participants; detailed information on wave 5 is included in a technical report. [28] Wave 5 was chosen as this was the most recent wave of data collection including physical activity questions, more recent sweeps have not included physical activity assessments.

Patient and public involvement

This study used publicly available secondary data from the UK Data Service (https://www.ukdataservice.ac.uk/). Patients and the public were not involved.

Measures

Domain specific physical activity

Active travel was measured in currently employed individuals and those not working from home via the question '*how do you usually get to your place of work?*' Responses were collapsed into a binary variable of 'non-active' (car, bus, or train/metro) or 'active' (walking or cycling). Occupational physical activity was measured by asking participants whether their job was mainly physical or not

For peer review only - http://bmjopen.bmj.com/site/about/guidelines.xhtml

(categorised as 'not physical' and 'physical'). Finally, LTPA variables were created from participant responses to the 'Taking part Survey', [29] a Department for Culture, Media and Sport survey on engagement with a range of different leisure time activities including sport. This includes an assessment of how often they participated in a series of prelisted sports and activities. Sports were then grouped into two categories based on their average metabolic equivalent of task (MET), those with METs of ≥3 were categorised as moderate-to-vigorous and METs 1.5-2.9 as light, using cut offs widely used in previous physical activity studies. [30] Frequency of participation in each MET-group was categorised as weekly or non-weekly.

Socio demographics

Highest educational attainment was self-reported and categorised into three groups: 'degree or higher (university level education typically undertaken after age 18), 'school diploma/ other qualification' (e.g. A levels and vocational diplomas, education to age 18) and 'GCSEs and below' (education to age 16—compulsory schooling age).

Ethnicity was self-reported and responses were collapsed into 'White', 'Black', 'Asian' and 'other' . These broad ethnic groupings include minority groups—'White' includes all white minorities such as Irish and Polish, Black includes Black-African and Black-Caribbean, while those of smaller sample sizes such as Arab and mixed-ethnicity were included in 'other'. Age at the time of interview was categorised into ten year age groups (from ages 20-60). Older adults were grouped from >60 years, and those below 20 (N= 3,050) were excluded from the analysis to ensure comparable sample sizes in the higher education groups—alternative groupings did not substantially affect the results (data available upon request).

Statistical analysis

We first cross-tabulated educational attainment by age, sex, and ethnicity. Next, logistic regression analyses were conducted to examine associations of education, sex, age, and ethnicity with physical activity in each domain. Analyses were assessed before and after mutual adjustment for each demographic variable. Those with missing demographic and education data yet valid outcome data were excluded from analysis (N= 1,583); analytical samples for active travel, occupational, and leisure were N= 18,404, N= 22,287 and N= 40,270 respectively. The differences in sample sizes by outcome was largely due to routing - only employed individuals were asked about occupational activity or active travel. A flow diagram (Supplementary Figure S1) displays the final sample size for each outcome. Finally, to examine possible effect modification of the associations between education and physical activity in each domain, we included two-way interaction terms (education x

For peer review only - http://bmjopen.bmj.com/site/about/guidelines.xhtml

ethnicity; education x sex; education x age) in addition to the relevant first order terms in the same model. Analyses were weighted according to sample design and attrition to reduce bias by under-coverage, sampling, or non-response.[26 31] Associations were presented as odds ratios (OR) with 95% confidence intervals (CI), while tests of moderation were presented as absolute differences in the predicated probability of each physical activity outcome comparing the highest and lowest education groups. All analyses were conducted using Stata, version 15.0 (StataCorp LP, College Station, Texas).

Results

Ethnicity, sex, and age were each independently associated with educational attainment (see Supplementary Table S1) and with physical activity in each domain ($p < 0.001$; see Table 1). Lower educational attainment was associated with higher active travel and occupational physical activity, but lower weekly light and moderate LTPA. White ethnicity was associated with higher LTPA, but less active travel and occupational physical activity. Additionally, males participated in more moderate-to-vigorous LTPA and occupational activity, but lower active travel than females.

Active travel to work

Active travel was lowest amongst individuals who were highly-educated, older, and male; there was little evidence for a strong association with ethnicity (See Table 2). The magnitude of education-related disparities were largest among females (education x sex $P<0.001$) and Black individuals (education x ethnicity $P= 0.038$) (See Figure 1 & Supplementary Tables S2-4). For example, the estimated difference in the probability of using active travel in the highest versus the lowest educational group was -10% (95% CI: -11.9, 7.9) amongst females and -3% (-4.8, -0.4) among males (see Supplementary tables S2-S4). Results for this domain, and all others, were similar when restricting to those with valid demographic and physical activity data, or when not making this restriction (Supplementary tables S5-6).

Occupational activity

Physically active occupations were less commonly reported amongst individuals who were highly-educated, white, and aged over 20-29; there was no evidence for association with sex (see Table 2). The magnitude of education-related disparities were largest among males (education x sex $P <0.001$), and those aged 30-39 (education x age $P= 0.001$) (See Figure 2 and Supplementary Tables S2-4). For example, the estimated difference in the probability of a physical occupation in the

For peer review only - http://bmjopen.bmj.com/site/about/guidelines.xhtml

highest versus lowest educational group was -35% (-36.9, -32.4) for males and -17% (-19.4, -15.0) for females (see Supplementary tables S2-S4).

Moderate-to-vigorous and light LTPA

Greater levels of participation in both weekly light and moderate-to-vigorous LTPA were reported amongst individuals who were highly educated and White. Males and younger adults were also more likely to report participation in moderate-to-vigorous LTPA, whereas females and older adult were more likely to report participation in light LTPA. (See Table 2).

The magnitude of education-related disparities in weekly moderate-to-vigorous LTPA was largest among White and Asian individuals (education x ethnicity P= 0.001), and those aged 40-49 and 50-59 (education x age P= 0.008) (See Figure 3 and Supplementary Tables S2-4). For example, the estimated probability of weekly moderate-to-vigorous LTPA in the highest versus lowest educational group was 17% (16.2, 18.7) for white individuals, compared with 6% (0.6, 11.6) for Black individuals, 16% (12.8, 19.1) for Asian individuals, and 13% (6.0, 19.5) for those of other ethnicity (see Supplementary tables S2-S4).

The magnitude of education-related disparities in weekly light LTPA was largest among females (education x sex P< 0.001) and individuals aged 60+ (education x age P< 0.001); there was little evidence for associations with ethnicity (See Figure 4 and Supplementary Tables S2-4). For example, the estimated probability of weekly light leisure time activity in the highest versus lowest educational group was 13% (11.4, 15.3) for those aged 60+ compared with 8% (5.9, 10.6) for those ages 50-59, 3% (1.3, 5.6) for those 40-49, 3% (0.9, 5.4) for those 30-39, and 2% (-.04, 4.7) for those aged 20-29 (see Supplementary tables S2-S4).

For peer review only

Discussion

Main findings and interpretations

In a large nationally representative dataset, educational attainment was associated with physical activity across three key domains; individuals with higher education were less likely to engage in active travel and occupational physical activity, but were more likely to engage in LTPA. These associations were modified by ethnicity, age, and sex. For active travel, educational disparities were largest among females and Black individuals. For occupational physical activity disparities were largest among males and those aged 30-39. For moderate-to-vigorous LTPA, educational disparities were largest among White and Asian individuals and those aged 40-49 and 50-59. Finally, for light LTPA, disparities were largest among females and those aged 60+.

Our findings may be explained by disparities in factors which affect physical activity levels such as health status, [32] environment, [33 34] cultural preferences, [35 36] financial resources, [25 37 38], perceived safety, [39 40] and domestic requirements. [41 42] These factors may differ between sociodemographic groups within education levels, resulting in differing magnitudes of disparities observed. The pathways involved may differ across each activity domain. For example, areas perceived as unsafe may result in reduced use for either travel and/or for leisure-time purposes. [43 44] Similarly, affordability of facilities (e.g. gym memberships) and other assets (e.g. cars) may yield different opportunities for participation in physical activity. English proficiency and work experience may also create unequal occupational opportunities. [43 44] While access times [41 42] and cultural expectations [36] may additionally contribute to differences in leisure-time participation.

Strengths and Limitations

Strengths of this study include a large nationally representative sample, enabling us to identify the previously seldom-examined role of ethnicity as a modifier of the relationship between educational attainment and physical activity across different domains. We also examined activity outcomes across three domains; previous studies investigating associations of physical activity typically use a single physical activity outcome measure, capturing either 'leisure' or 'unspecified' activity. [4 10]

There are also a number of limitations to consider. First, while we obtained information across multiple domains, we lack detailed information on activity duration. However, the LTPA measures used followed expected patterns for these leisure time categories by sex and age. [45] We also did not consider perceptions of the local environment including safety which may affect physical activity. [39 40] Second, we did not capture physical housework as a domain, which includes domestic and

For peer review only - http://bmjopen.bmj.com/site/about/guidelines.xhtml

cleaning tasks, gardening, and do-it-yourself (DIY). Third, all physical-activity measures were captured via self-report; while this is needed to investigate domain-specific activity, it may be subject to recall bias with individuals' either over or under reporting their levels of physical activity. [46] For example, previous evidence has shown that males and those of lower education were more likely to overestimate their physical activity levels than females and those with higher education, respectively. [47] Differential reporting bias across population sub groups[48] could therefore bias our finding of effect modification. Insofar as objective measurements of physical activity are able to capture domain-specific activity, they may be useful to include in future studies to help verify our findings. Fourth, only working adults could be included in the analyses of the active travel and occupational domains; investigation of multiple types of physical activity among retirees, those currently seeking work, or those unable to work warrants consideration in the future. Fifth, bias may be introduced through excluding missing data and non-responders, although missing data due to item missingness (as opposed to specific question gating) was low and therefore bias unlikely We also used weights to reduce bias caused by under-coverage, sampling, or non-response. [31] Sixth, due to the cross-sectional design we cannot separate out age from birth cohort effects, and so future cross-cohort studies are required to address this. Finally, this study identified cross-sectional associations of education with physical activity across key domains, as well as differences by ethnicity, sex, and age in these associations. While we hypothesised that the primary direction of causality was from education attainment to physical activity outcomes, physical activity may affect educational attainment at younger ages.[49] Further longitudinal analyses may provide stronger evidence on the causal nature of the observed associations and additionally identify the mediators of the disparities observed.

Implications for practice, and policy

Our findings may have important implications for practice and policy. The inequalities in LTPA observed—across both light and moderate-vigorous activity—suggests that policies are required to reduce these inequalities given the multiple anticipated effects on health. Population-level or targeted interventions may be used to reduce the sizeable modification across demographic sub-groups. For example, there was a 13% difference across education levels in the probability of participating in light LTPA amongst those aged 60+ compared with 2-3% of those aged 20-39, suggesting older adults with lower levels of education would benefit most from interventions regarding this domain of physical activity. Furthermore, in line with previous evidence [19] we also found that those of lower educational attainment were more likely to possess physically demanding occupations. This difference is important, if the health consequences of occupational physical

For peer review only - http://bmjopen.bmj.com/site/about/guidelines.xhtml

activity are less favourable or detrimental compared with LTPA. [5 6] Efforts to increase LTPA and its inequality should consider co-occurring differences in occupational activity. Finally, lower participation in active travel was also found in those with higher levels of educational attainment. For example, there was a 10% difference in active travel among high to low educated women compared with 3% in men. Previous evidence has found similar sex differences in cycling to work; however, similar proportions of men and women report leisure-time cycling. [50] Suggested means of increasing active travel include the provision of safe walking and cycling travel routes, accessible bike locks, and changing facilities.

Our findings may have implications for future studies which investigate inequalities in physical activity outcomes. Existing studies typically adjust for the sociodemographic factors we investigated as potential modifiers. Given the evidence for modification that we found, such analyses may provide biased estimates of the magnitude of inequalities that operate in particular population sub-groups.

Conclusions

In summary, we found sex, age, and ethnicity modified associations between educational attainment and multiple physical activity outcomes. Our findings imply there may be unequal access or additional barriers to physical activity across both education and demographic sub-groups. Better understanding the characteristics of physically inactive sub-groups may aid development of tailored interventions to increase activity levels and reduce health inequalities.

For peer review only

Table 1. Physical activity domains by education and demographic characteristics in Understanding Society (2013-2015)

**Demographic**	**Physical activity domain**															
	**Active transportation**				**Occupational**				**Leisure-time**							
									**Moderate-to-vigorous**				**Light**			
	Non-active		Active		Not physical		Physical		< Weekly		≥ 1x weekly		< Weekly		≥ 1x weekly	
	N	*%*	*N*	*%*	*N*	*%*	*N*	*%*	*N*	*%*	*N*	*%*	*N*	*%*	*N*	*%*
**Educational attainment**																
GCSEs and lower	3639	81.1	846	18.9	1471	27.3	3920	72.7	11048	79.8	2798	20.2	11482	82.9	2364	17.1
School diploma	4547	83.4	906	16.6	2200	33.4	4383	66.6	8857	71.3	3570	28.7	9910	79.8	2517	20.3
Degree/higher	7519	87.5	1078	12.5	5394	51.6	5063	48.4	9358	60.7	6069	39.3	11914	77.2	3513	22.8
P value				<0.001				<0.001				<0.001				<0.001
**Ethnicity**																
White	13397	84.8	2396	15.2	8001	41.7	11199	58.3	23749	68.7	10822	31.3	26892	77.8	7679	22.2
Black	631	86.2	101	13.8	227	27.7	594	72.4	1047	72.3	402	27.7	1308	90.3	141	9.7
Asian	1223	83.9	234	16.1	598	34.1	1158	66.0	2571	74.9	864	25.2	3030	88.2	405	11.8
Other ethnicity	366	81.7	82	18.3	202	37.4	338	62.6	609	67.0	300	33.0	764	84.1	145	16.0
P value				<0.001				<0.001				<0.001				<0.001
**Gender**																
Male	7076	85.4	1215	14.7	4296	40.3	6376	59.8	13305	69.0	5979	31.0	15741	81.6	3543	18.4
Female	8656	84.2	1621	15.8	4780	40.5	7018	59.5	16084	71.3	6485	28.7	17702	78.4	4867	21.6
P value				<0.001				<0.001				<0.001				<0.001
**Age**																
20-29 years	2703	81.0	633	19.0	1235	34.2	2381	65.9	3848	62.9	2272	37.1	5188	84.8	932	15.2
30-39 years	3650	85.2	636	14.8	2171	43.3	2841	56.7	4220	62.1	2579	37.9	5704	83.9	1095	16.1
40-49 years	4511	86.3	718	13.7	2730	43.0	3622	57.0	5526	66.1	2835	33.9	6679	79.9	1682	20.1
50-59 years	3594	84.9	640	15.1	2108	40.2	3137	59.8	5479	72.8	2043	27.2	5855	77.8	1667	22.2
60+ years	1274	85.9	209	14.1	832	37.1	1413	62.9	10316	79.0	2735	21.0	10017	76.8	3034	23.3

P value	<0.001	<0.001	<0.001	<0.001

Participants from wave 5 (2013-2015) of Understanding Society with data on educational attainment, demographics, and physical activity

P value = chi^{2},

For peer review only

Table 2. Mutually adjusted associations of educational attainment and demographic characteristics with domain-specific physical activity outcomes

	Physical Activity															
	Active travel				Occupational				Leisure time							
									Moderate-to-vigorous				Light			
	N=18,404				N=22,287				N= 40,270				N= 40,270			
	OR	*95% CI*		*p*	*OR*	*95% CI*		*p*	*OR*	*95% CI*		*p*	*OR*	*95% CI*		*p*
**Education**																
GCSEs and lower	**ref*															
School diploma	0.83	0.74	0.93	0.001	0.73	0.67	0.79	<0.001	1.44	1.35	1.53	<0.001	1.33	1.24	1.42	<0.001
Degree/higher	0.59	0.53	0.66	<0.001	0.34	0.31	0.37	<0.001	2.28	2.16	2.42	<0.001	1.58	1.49	1.69	<0.001
**Ethnicity**																
White	**ref*															
Black	0.94	0.74	1.19	0.620	2.19	1.84	2.59	<0.001	0.69	0.61	0.77	<0.001	0.38	0.31	0.46	<0.001
Asian	1.10	0.95	1.29	0.211	1.56	1.38	1.75	<0.001	0.59	0.54	0.64	<0.001	0.51	0.46	0.57	<0.001
Other ethnicity	1.31	1.01	1.69	0.041	1.40	1.16	1.70	0.001	0.82	0.71	0.95	0.008	0.69	0.57	0.83	<0.001
**Sex**																
Male	**ref*															
Female	1.11	1.02	1.21	0.016	1.04	0.99	1.10	0.150	0.84	0.81	0.88	<0.001	1.20	1.14	1.26	<0.001
**Age**																
20-29 years	**ref*															
30-39 years	0.77	0.67	0.88	<0.001	0.71	0.65	0.79	<0.001	0.96	0.88	1.04	0.287	1.02	0.92	1.13	0.676
40-49 years	0.67	0.59	0.76	<0.001	0.68	0.62	0.75	<0.001	0.81	0.75	0.88	<0.001	1.36	1.24	1.50	<0.001
50-59 years	0.74	0.64	0.84	<0.001	0.74	0.68	0.82	<0.001	0.59	0.55	0.64	<0.001	1.52	1.39	1.67	<0.001
60+ years	0.66	0.55	0.79	<0.001	0.83	0.74	0.94	0.003	0.44	0.41	0.48	<0.001	1.64	1.50	1.80	<0.001

Participants from wave 5 (2013-2015) of Understanding Society with valid data on educational attainment, demographics, and physical activity
Samples were restricted to those with valid demographic and physical activity data
Active travel to work: non-active/active; Occupational: non-physical/physical; Leisure-time: <weekly/ ≥ 1x weekly
Analyses are mutually adjusted for education, age, sex, and ethnicity
OR= Odds Ratio, CI= 95% Confidence intervals

**reference group*

References

1. Warburton DE, Nicol CW, Bredin SS. Health benefits of physical activity: the evidence. *CMAJ* 2006;174(6):801-9. doi: 10.1503/cmaj.051351
2. Kohl HW, 3rd, Craig CL, Lambert EV, et al. The pandemic of physical inactivity: global action for public health. *Lancet* 2012;380(9838):294-305. doi: 10.1016/S0140-6736(12)60898-8
3. Ding D, Lawson KD, Kolbe-Alexander TL, et al. The economic burden of physical inactivity: a global analysis of major non-communicable diseases. *Lancet* 2016;388(10051):1311-24. doi: 10.1016/S0140-6736(16)30383-X
4. Scholes S, Bann D. Education-related disparities in reported physical activity during leisure-time, active transportation, and work among US adults: repeated cross-sectional analysis from the National Health and Nutrition Examination Surveys, 2007 to 2016. *Bmc Public Health* 2018;18(1):926. doi: 10.1186/s12889-018-5857-z
5. Holtermann A, Hansen JV, Burr H, et al. The health paradox of occupational and leisure-time physical activity. *Br J Sports Med* 2012;46(4):291-5. doi: 10.1136/bjsm.2010.079582 [published Online First: 2011/04/05]
6. Holtermann A, Krause N, van der Beek AJ, et al. The physical activity paradox: six reasons why occupational physical activity (OPA) does not confer the cardiovascular health benefits that leisure time physical activity does. *Br J Sports Med* 2018;52(3):149-50. doi: 10.1136/bjsports-2017-097965 [published Online First: 2017/08/12]
7. Elhakeem A, Cooper R, Bann D, et al. Childhood socioeconomic position and adult leisure-time physical activity: a systematic review. *Int J Behav Nutr Phys Act* 2015;12:92. doi: 10.1186/s12966-015-0250-0
8. Bleich SN, Jarlenski MP, Bell CN, et al. Health inequalities: trends, progress, and policy. *Annu Rev Public Health* 2012;33:7-40. doi: 10.1146/annurev-publhealth-031811-124658
9. Droomers M, Schrijvers CT, Mackenbach JP. Educational level and decreases in leisure time physical activity: predictors from the longitudinal GLOBE study. *J Epidemiol Community Health* 2001;55(8):562-8. [published Online First: 2001/07/13]
10. Harper S, Lynch J. Trends in socioeconomic inequalities in adult health behaviors among US states, 1990-2004. *Public Health Rep* 2007;122(2):177-89. doi: Doi 10.1177/003335490712200207
11. Bann D, Johnson W, Li L, et al. Socioeconomic inequalities in childhood and adolescent body-mass index, weight, and height from 1953 to 2015: an analysis of four longitudinal, observational, British birth cohort studies. *Lancet Public Health* 2018;3(4):E194-E203. doi: 10.1016/S2468-2667(18)30045-8
12. Hayes L, White M, Unwin N, et al. Patterns of physical activity and relationship with risk markers for cardiovascular disease and diabetes in Indian, Pakistani, Bangladeshi and European adults in a UK population. *J Public Health Med* 2002;24(3):170-8.
13. Obesity and ethnicity. In: Health SfP, ed.: National Obesity Observatory, 2011.
14. Fischbacher CM, Hunt S, Alexander L. How physically active are South Asians in the United Kingdom? A literature review. *J Public Health (Oxf)* 2004;26(3):250-8. doi: 10.1093/pubmed/fdh158
15. Guthold R, Stevens GA, Riley LM, et al. Worldwide trends in insufficient physical activity from 2001 to 2016: a pooled analysis of 358 population-based surveys with 1.9 million participants. *Lancet Glob Health* 2018;6(10):e1077-e86. doi: 10.1016/S2214-109X(18)30357-7 [published Online First: 2018/09/09]
16. Bécares L. The Ethnic Density effect on the Health of Ethnic Minority People in the United Kingdom: a Study of Hypothesised Pathways. London: University College London, 2009.

For peer review only - http://bmjopen.bmj.com/site/about/guidelines.xhtml

17. Pickett KE, Shaw RJ, Atkin K, et al. Ethnic density effects on maternal and infant health in the Millennium Cohort Study. *Soc Sci Med* 2009;69(10):1476-83. doi: 10.1016/j.socscimed.2009.08.031
18. Saffer H, Dave D, Grossman M, et al. Racial, Ethnic, and Gender Differences in Physical Activity. *J Hum Cap* 2013;7(4):378-410. doi: 10.1086/671200
19. He XZ, Baker DW. Differences in leisure-time, household, and work-related physical activity by race, ethnicity, and education. *J Gen Intern Med* 2005;20(3):259-66. doi: 10.1111/j.1525-1497.2005.40198.x
20. Evandrou M, Falkingham J, Feng ZX, et al. Ethnic inequalities in limiting health and self-reported health in later life revisited. *J Epidemiol Commun H* 2016;70(7):653-62. doi: 10.1136/jech-2015-206074
21. Farrell L, Hollingsworth B, Propper C, et al. The socioeconomic gradient in physical inactivity: Evidence from one million adults in England. *Soc Sci Med* 2014;123:55-63. doi: 10.1016/j.socscimed.2014.10.039
22. Stalsberg R, Pedersen AV. Effects of socioeconomic status on the physical activity in adolescents: a systematic review of the evidence. *Scand J Med Sci Sports* 2010;20(3):368-83. doi: 10.1111/j.1600-0838.2009.01047.x [published Online First: 2010/02/09]
23. Berger U, Der G, Mutrie N, et al. The impact of retirement on physical activity. *Ageing Soc* 2005;25:181-95. doi: 10.1017/S0144686x04002739
24. Mutrie N, Hannah MK. Some work hard while others play hard. *International Journal of Health Promotion and Education* 2013;42(2):109-17. doi: 10.1080/14635240.2004.10708024
25. Stringhini S, Sabia S, Shipley M, et al. Association of socioeconomic position with health behaviors and mortality. *JAMA* 2010;303(12):1159-66. doi: 10.1001/jama.2010.297 [published Online First: 2010/03/25]
26. Knies G. Unverstanding Society: The UK Household Longitundal Study: Waves 1-8 User Guide Essex: University of Essex; 2018 [
27. Peterson J, Rabe B. Understanding Society – a geographical profile of respondents University of Essex Available from: https://www.understandingsociety.ac.uk/sites/default/files/downloads/working-papers/2013-01.pdf]
28. Jessop C. UK Household Longitudinal Study Wave 5 Technical Report. London: NatCen Social Research, 2015. [Available from:http://doc.ukdataservice.ac.uk/doc/6676/mrdoc/pdf/6676_wave5_technical_report.pdf]
29. Department for Digital C, Media & Sport. Taking Part Survey 2016 [Available from: https://www.gov.uk/guidance/taking-part-survey#the-taking-part-survey.
30. Ainsworth BE, Haskell WL, Herrmann SD, et al. 2011 Compendium of Physical Activities: a second update of codes and MET values. *Med Sci Sports Exerc* 2011;43(8):1575-81. doi: 10.1249/MSS.0b013e31821ece12 [published Online First: 2011/06/18]
31. Lynn P, Kaminska O. Weighting strategy for Understanding Society. Understanding Society Working Paper Series: Institute for Social and Economic Research, University of Essex.
32. Bauman AE, Reis RS, Sallis JF, et al. Correlates of physical activity: why are some people physically active and others not? *Lancet* 2012;380(9838):258-71. doi: 10.1016/S0140-6736(12)60735-1 [published Online First: 2012/07/24]
33. In: CABE, ed. Community green: using local spaces to tackle inequality and improve health. London.
34. Bird S, Kurowski W, Feldman S, et al. The influence of the built environment and other factors on the physical activity of older women from different ethnic communities. *J Women Aging* 2009;21(1):33-47. doi: 10.1080/08952840802633669
35. Rojas A, Storch EA. Psychological complications of obesity. *Pediatr Ann* 2010;39(3):174-80. doi: 10.3928/00904481-20100223-07

For peer review only - http://bmjopen.bmj.com/site/about/guidelines.xhtml

36. Long J, Hylton K, Spracklen K, et al. Long J, Hylton K, Spracklen K, Ratna A, Bailey S. Sporting Equals. A Systematic Review of the Literature on Black and Minority Ethnic Communities on Sport and Physical Recreation. Leeds: Carnegie Institute, 2009.
37. Finger JD, Tylleskar T, Lampert T, et al. Physical activity patterns and socioeconomic position: the German National Health Interview and Examination Survey 1998 (GNHIES98). *Bmc Public Health* 2012;12:1079. doi: 10.1186/1471-2458-12-1079 [published Online First: 2012/12/18]
38. Popham F, Mitchell R. Relation of employment status to socioeconomic position and physical activity types. *Prev Med* 2007;45(2-3):182-8. doi: 10.1016/j.ypmed.2007.06.012 [published Online First: 2007/07/31]
39. Panter JR, Jones AP, van Sluijs EM. Environmental determinants of active travel in youth: a review and framework for future research. *Int J Behav Nutr Phys Act* 2008;5:34. doi: 10.1186/1479-5868-5-34 [published Online First: 2008/06/25]
40. Hooker SP, Wilson DK, Griffin SF, et al. Perceptions of environmental supports for physical activity in African American and white adults in a rural county in South Carolina. *Prev Chronic Dis* 2005;2(4):A11. [published Online First: 2005/09/17]
41. Telama R, Yang X, Viikari J, et al. Physical activity from childhood to adulthood: a 21-year tracking study. *Am J Prev Med* 2005;28(3):267-73. doi: 10.1016/j.amepre.2004.12.003
42. Telama R. Tracking of physical activity from childhood to adulthood: a review. *Obes Facts* 2009;2(3):187-95. doi: 10.1159/000222244
43. Hispanics in the U.S. Labor Market: Selected Research. *Hispanics in the US Labor Market: Selected Research* 2014:1-146.
44. Employment by occupation. In: Department for Business EIS, Gov.uk, ed. England, 2018.
45. Caspersen CJ, Pereira MA, Curran KM. Changes in physical activity patterns in the United States, by sex and cross-sectional age. *Med Sci Sports Exerc* 2000;32(9):1601-9. [published Online First: 2000/09/20]
46. Mann CJ. Observational research methods. Research design II: cohort, cross sectional, and case-control studies. *Emerg Med J* 2003;20(1):54-60. [published Online First: 2003/01/21]
47. Watkinson C, van Sluijs EM, Sutton S, et al. Overestimation of physical activity level is associated with lower BMI: a cross-sectional analysis. *Int J Behav Nutr Phys Act* 2010;7:68. doi: 10.1186/1479-5868-7-68 [published Online First: 2010/09/22]
48. Kapteyn A, Banks J, Hamer M, et al. What they say and what they do: comparing physical activity across the USA, England and the Netherlands. *J Epidemiol Community Health* 2018;72(6):471-76. doi: 10.1136/jech-2017-209703 [published Online First: 2018/04/13]
49. . In: Kohl HW, III, Cook HD, eds. Educating the Student Body: Taking Physical Activity and Physical Education to School. Washington (DC)2013.
50. Heesch KC, Sahlqvist S, Garrard J. Gender differences in recreational and transport cycling: a cross-sectional mixed-methods comparison of cycling patterns, motivators, and constraints. *Int J Behav Nutr Phys Act* 2012;9:106. doi: 10.1186/1479-5868-9-106 [published Online First: 2012/09/11]

For peer review only - http://bmjopen.bmj.com/site/about/guidelines.xhtml

Figures 1-4 Legend

Data: Understanding Society, Wave 5. Dotted line represents reference group (GCSE & lower). P values indicate demographic x education.

Funding

This project is part of a collaborative research programme entitled 'Cohorts and Longitudinal Studies Enhancement Resources' (CLOSER) funded by the ESRC (http://www.esrc.ac.uk) (ES/K000357/1). DB and MF are also supported by the Academy of Medical Sciences/the Wellcome Trust "Springboard Health of the public in 2040" Award (HOP001\1025). SPP is funded by a UK Medical Research Council Career Development Award (MR/P020372/1). MB is funded by ESRC ES/N00812X/1. Understanding Society is funded by the Economic and Social Research Council (ES/N00812X/1).

Competing interests

None to declare.

Author contributions

MF, DB, SPP and RC were involved in the conception and design of the study, MF conducted the analyses and drafted the manuscript, and MF, SPP, MB, MH, BJ, LG, RC and DB have revised the manuscript and approved for publication.

Data availability statement

Data for this study, Understanding Society, was accessed through the UK Data Service (https://www.ukdataservice.ac.uk).

Licence statement

I, the Submitting Author has the right to grant and does grant on behalf of all authors of the Work (as defined in the below author licence), an exclusive licence and/or a non-exclusive licence for contributions from authors who are: i) UK Crown employees; ii) where BMJ has agreed a CC-BY licence shall apply, and/or iii) in accordance with the terms applicable for US Federal Government officers or employees acting as part of their official duties; on a worldwide, perpetual, irrevocable, royalty-free basis to BMJ Publishing Group Ltd ("BMJ") its licensees and where the relevant Journal is co-owned by BMJ to the co-owners of the Journal, to publish the Work in BMJ Open and any other BMJ products and to exploit all rights, as set out in our licence.

For peer review only

The Submitting Author accepts and understands that any supply made under these terms is made by BMJ to the Submitting Author unless you are acting as an employee on behalf of your employer or a postgraduate student of an affiliated institution which is paying any applicable article publishing charge ("APC") for Open Access articles. Where the Submitting Author wishes to make the Work available on an Open Access basis (and intends to pay the relevant APC), the terms of reuse of such Open Access shall be governed by a Creative Commons licence – details of these licences and which Creative Commons licence will apply to this Work are set out in our licence referred to above.

For peer review only

For peer review only

Figure 1. Educational differences in active travel by sex, ethnicity, and age

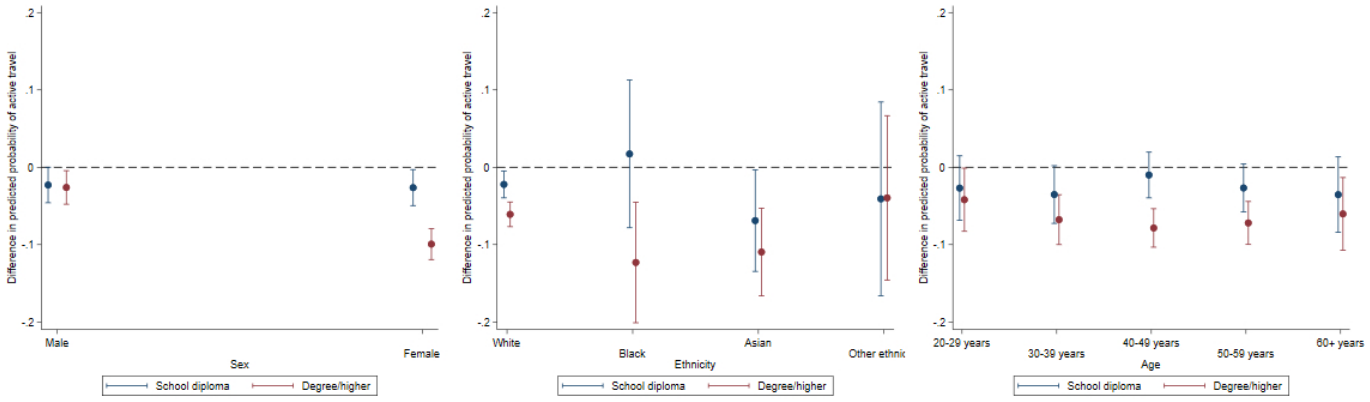

Figure 2. Educational differences in occupational physical activity by sex, ethnicity, and age

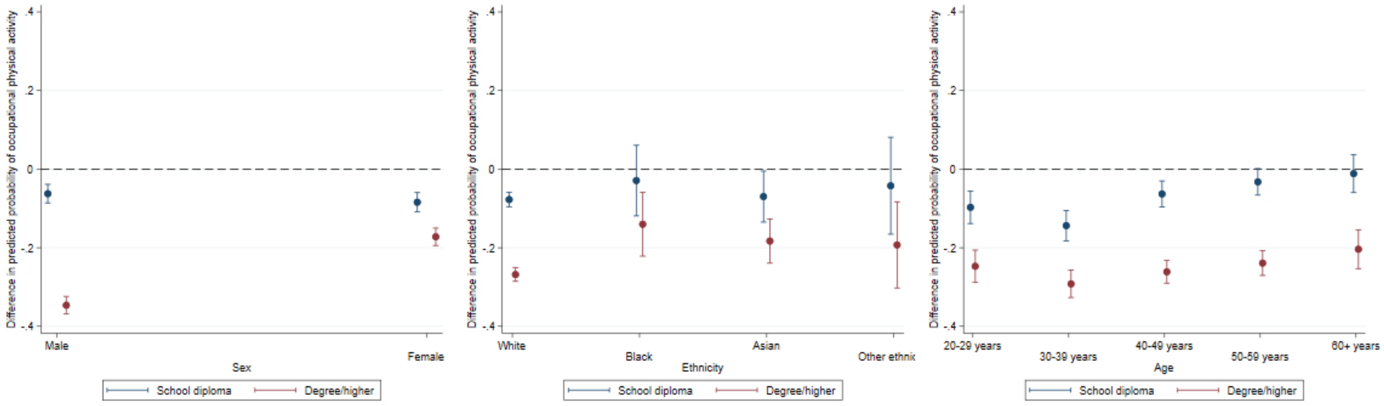

For peer review only

Figure 3. Educational differences in moderate to vigorous intesity activities by sex, ethnicity, and age

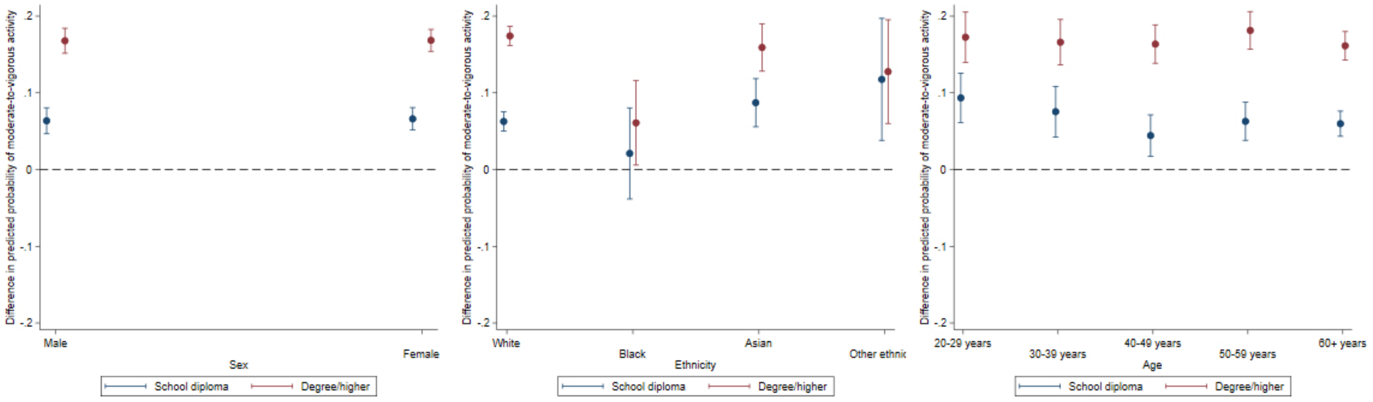

Figure 4. Educational differences in light intesity activities by sex, ethnicity, and age

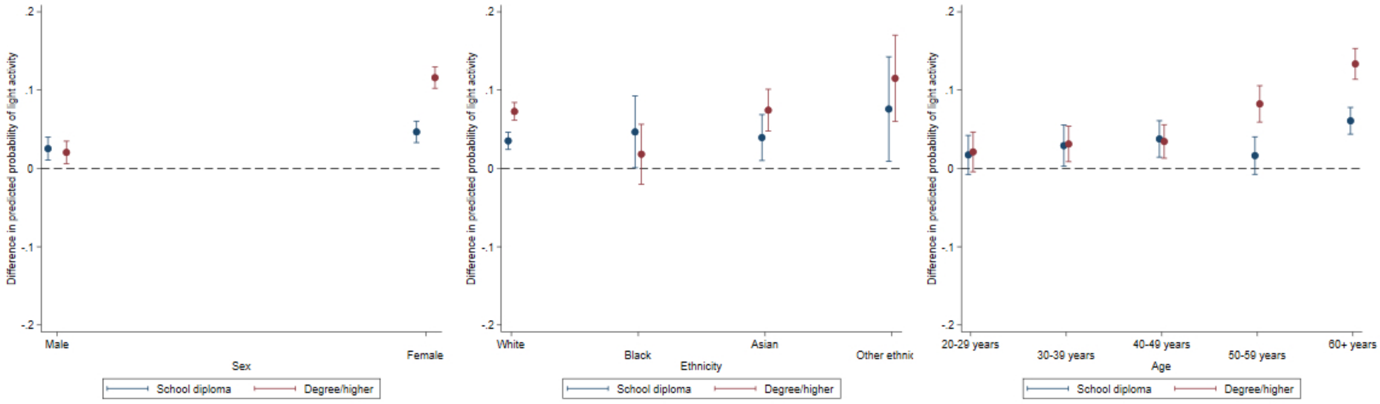

For peer review only

Table S1. Ethnicity, sex and age by highest educational attainment in Understanding Society (2013-2015)

Demographics	Highest educational attainment					
	GCSEs and lower		School diploma & other qualifications		Degree/higher	
	N	*(%)*	N	*(%)*	N	*(%)*
**Ethnicity**						
White	11638	33.7	10424	30.2	12424	35.9
Black	363	25.1	400	27.6	685	47.3
Asian	1106	32.2	877	25.5	1445	42.1
Other ethnicity	201	22.1	235	25.9	472	51.9
Missing	538	36.1	491	33	401	26.9
P value	<0.001					
**Sex**						
Male	5888	30.5	6388	33.1	6927	35.9
Female	7958	35.3	6039	26.8	8500	37.7
P value	<0.001					
**Age**						
20-29 years	1462	23.9	2363	38.6	2271	37.1
30-39 years	1653	24.3	1776	26.1	3345	49.2
40-49 years	2433	29.1	2339	28	3570	42.7
50-59 years	2455	32.6	2221	29.5	2822	37.5
60+	5843	44.8	3728	28.6	3419	26.2
P value	<0.001					

Participants from wave 5 (2013-2015) of Understanding Society with data on educational attainment, demographics, and physical activity
Missing = missing data; not shown where data was complete.
P value derived from chi-squared tests[2,]

For peer review only

Table S2. Education differences in physical activity by age group

Physical activity	20-29				30-39				40-49				50-59				60+				education X age
	Coef.	*95% CI*		*p*	*Coef.*	*95% CI*		*p*	*Coef.*	*95% CI*		*p*	*Coef.*	*95% CI*		*p*	*Coef.*	*95% CI*		*p*	
**Active Travel**																					
GCSEs and lower	**ref*																				
School diploma	-0.03	-0.07	0.01	0.206	-0.04	-0.07	0.00	0.064	-0.01	-0.04	0.02	0.506	-0.03	-0.06	0.00	0.091	-0.04	-0.08	0.01	0.155	0.081
Degree/higher	-0.04	-0.08	0.00	0.044	-0.07	-0.10	-0.04	<0.001	-0.08	-0.10	-0.05	<0.001	-0.07	-0.10	-0.04	<0.001	-0.06	-0.11	-0.01	0.012	
**Occupational**																					
GCSEs and lower	**ref*																				
School diploma	-0.10	-0.14	-0.06	<0.001	-0.14	-0.18	-0.10	<0.001	-0.06	-0.10	-0.03	<0.001	-0.03	-0.07	0.00	0.060	-0.01	-0.06	0.04	0.648	0.001
Degree/higher	-0.25	-0.29	-0.21	<0.001	-0.29	-0.33	-0.26	<0.001	-0.26	-0.29	-0.23	<0.001	-0.24	-0.27	-0.21	<0.001	-0.20	-0.25	-0.15	<0.001	
**Moderate-to-vigorous leisure time**																					
GCSEs and lower	**ref*																				
School diploma	0.09	0.06	0.13	<0.001	0.08	0.04	0.11	<0.001	0.04	0.02	0.07	0.001	0.06	0.04	0.09	<0.001	0.06	0.04	0.08	<0.001	0.008
Degree/higher	0.17	0.14	0.21	<0.001	0.17	0.14	0.20	<0.001	0.16	0.14	0.19	<0.001	0.18	0.16	0.21	<0.001	0.16	0.14	0.18	<0.001	
**Light leisure time**																					
GCSEs and lower	**ref*																				
School diploma	0.02	-0.01	0.04	0.173	0.03	0.00	0.06	0.030	0.04	0.01	0.06	0.002	0.02	-0.01	0.04	0.180	0.06	0.04	0.08	<0.001	<0.001
Degree/higher	0.02	0.00	0.05	0.102	0.03	0.01	0.05	0.007	0.03	0.01	0.06	0.002	0.08	0.06	0.11	<0.001	0.13	0.11	0.15	<0.001	

Estimates are derived from separate logistic regression models of each binary physical activity outcome, and show the education-outcome associations on the absolute scale (predicted probability of activity) in each sociodemographic sub group
Participants from wave 5 (2013-2015) of Understanding Society with data on educational attainment, demographics, and physical activity
**reference groups*

Table S3. Education differences in physical activity by sex

Physical activity	Male			Female				education X sex
	Coef.	*95% CI*		*p*	*Coef.*	*95% CI*		*p*	
**Active Travel**									
GCSEs and lower	**ref*								
School diploma	-0.02	-0.05	0.00	0.050	-0.03	-0.05	0.00	0.027	<0.001
Degree/higher	-0.03	-0.05	0.00	0.018	-0.10	-0.12	-0.08	0.000	
**Occupational**									
GCSEs and lower	**ref*								
School diploma	-0.06	-0.09	-0.04	<0.001	-0.08	-0.11	-0.06	<0.001	<0.001
Degree/higher	-0.35	-0.37	-0.32	<0.001	-0.17	-0.19	-0.15	<0.001	
**Moderate-to-vigorous leisure time**									
GCSEs and lower	**ref*								
School diploma	0.06	0.05	0.08	<0.001	0.07	0.05	0.08	<0.001	0.550
Degree/higher	0.17	0.15	0.18	<0.001	0.17	0.15	0.18	<0.001	
**Light leisure time**									
GCSEs and lower	**ref*								
School diploma	0.03	0.01	0.04	0.001	0.05	0.03	0.06	<0.001	<0.001
Degree/higher	0.02	0.01	0.03	0.005	0.12	0.10	0.13	<0.001	

Estimates are derived from separate logistic regression models of each binary physical activity outcome, and show the education-outcome associations on the absolute scale (predicted probability of activity) in each sociodemographic sub group
Participants from wave 5 (2013-2015) of Understanding Society with data on educational attainment, demographics, and physical activity
**reference group*

Table S4. Education differences in physical activity by ethnicity

Physical activity	White				Black				Asian				Other ethnicity				education X ethnicity
	Coef.	*95% CI*		*p*	*Coef.*	*95% CI*		*p*	*Coef.*	*95% CI*		*p*	*Coef.*	*95% CI*		*p*	P
**Active Travel**																	
GCSEs and lower	**ref*																
School diploma	-0.02	-0.04	0.00	0.013	0.02	-0.08	0.11	0.723	-0.07	-0.13	0.00	0.040	-0.04	-0.17	0.08	0.523	0.038
Degree/higher	-0.06	-0.08	-0.04	<0.001	-0.12	-0.20	-0.05	0.002	-0.11	-0.17	-0.05	<0.001	-0.04	-0.15	0.07	0.465	
**Occupational**																	
GCSEs and lower	**ref*																
School diploma	-0.08	-0.10	-0.06	<0.001	-0.03	-0.12	0.06	0.526	-0.07	-0.13	0.00	0.035	-0.04	-0.17	0.08	0.502	0.155
Degree/higher	-0.27	-0.29	-0.25	<0.001	-0.14	-0.22	-0.06	0.001	-0.18	-0.24	-0.13	<0.001	-0.19	-0.30	-0.08	0.001	
**Moderate-to-vigorous leisure time**																	
GCSEs and lower	**ref*																
School diploma	0.06	0.05	0.08	<0.001	0.02	-0.04	0.08	0.488	0.09	0.06	0.12	<0.001	0.12	0.04	0.20	0.004	0.001
Degree/higher	0.17	0.16	0.19	<0.001	0.06	0.01	0.12	0.030	0.16	0.13	0.19	<0.001	0.13	0.06	0.20	<0.001	
**Light leisure time**																	
GCSEs and lower	**ref*																
School diploma	0.04	0.02	0.05	<0.001	0.05	0.00	0.09	0.046	0.04	0.01	0.07	0.008	0.08	0.01	0.14	0.026	0.057
Degree/higher	0.07	0.06	0.08	<0.001	0.02	-0.02	0.06	0.351	0.07	0.05	0.10	<0.001	0.12	0.06	0.17	<0.001	

Estimates are derived from separate logistic regression models of each binary physical activity outcome, and show the education-outcome associations on the absolute scale (predicted probability of activity) in each sociodemographic sub group
Participants from wave 5 (2013-2015) of Understanding Society with data on educational attainment, demographics, and physical activity
**reference group*

Table S5. Unadjusted associations (OR (95% CI)) of education and demographics with domain specific PA (complete case analysis)

	Physical Activity															
	**Active travel**				**Occupational**				**Leisure**							
									**Moderate-to-vigorous**				**Light**			
	N=18,404				*N=22,287*				*N= 40,270*				*N= 40,270*			
	OR	*95% CI*		*p*	*OR*	*95% CI*		*p*	*OR*	*95% CI*		*p*	*OR*	*95% CI*		*p*
**Education**																
GCSEs and lower	**ref*															
School diploma	0.86	0.77	0.96	0.006	0.75	0.69	0.81	<0.001	1.59	1.50	1.69	<0.001	1.23	1.16	1.31	<0.001
Degree/higher	0.62	0.55	0.68	<0.001	0.35	0.33	0.38	<0.001	2.55	2.41	2.69	<0.001	1.41	1.33	1.50	<0.001
**Ethnicity**																
White	**ref*															
Black	0.89	0.71	1.13	0.358	1.87	1.59	2.20	<0.001	0.84	0.75	0.95	0.005	0.38	0.31	0.45	<0.001
Asian	1.07	0.92	1.25	0.388	1.38	1.23	1.55	<0.001	0.74	0.68	0.81	<0.001	0.47	0.42	0.52	<0.001
Other ethnicity	1.25	0.97	1.61	0.080	1.20	0.99	1.44	0.061	1.08	0.94	1.25	0.277	0.66	0.55	0.80	<0.001
**Sex**																
Male	**ref*															
Female	1.09	1.01	1.19	0.033	0.99	0.94	1.05	0.822	0.85	0.82	0.89	<0.001	1.17	1.12	1.23	<0.001
**Age**																
20-29 years	**ref*															
30-39 years	0.75	0.65	0.85	<0.001	0.68	0.62	0.75	<0.001	1.01	0.93	1.09	0.875	1.05	0.95	1.16	0.353
40-49 years	0.68	0.60	0.77	<0.001	0.69	0.63	0.76	<0.001	0.84	0.78	0.90	<0.001	1.38	1.25	1.51	<0.001
50-59 years	0.76	0.67	0.87	<0.001	0.78	0.71	0.85	<0.001	0.60	0.56	0.65	<0.001	1.56	1.42	1.71	<0.001
60+ years	0.70	0.58	0.83	<0.001	0.88	0.79	0.99	0.034	0.43	0.39	0.46	<0.001	1.64	1.50	1.80	<0.001

Participants from wave 5 (2013-2015) of Understanding Society with data on educational attainment, demographics, and physical activity
Samples were restricted to those with valid demographic and physical activity data
*Active travel to work: non-active/active; Occupational: non-physical/physical; Leisure-time: <weekly/ ≥ 1x weekly*reference groups*
OR= Odds Ratio, CI= 95% Confidence intervals

Table S6. Unadjusted associations (OR (95% CI)) of education and demographics with domain specific PA (maximum available samples)

	Physical Activity																			
	Active travel					Occupational					Leisure									
											Moderate-to-vigorous					Light				
	N	*OR*	*95% CI*		*p*	N	*OR*	*95% CI*		*p*	N	*OR*	*95% CI*		*p*	N	*OR*	*95% CI*		*p*
**Education**																				
GCSEs and lower	18535	**ref*				22431					41700					41700				
School diploma		0.86	0.77	0.96	0.006		0.75	0.69	0.81	<0.001		1.59	1.50	1.69	<0.001		1.23	1.16	1.31	<0.001
Degree/higher		0.62	0.55	0.69	<0.001		0.35	0.33	0.38	<0.001		2.56	2.43	2.70	<0.001		1.43	1.35	1.52	<0.001
**Ethnicity**																				
White	18430	**ref*				22317					40364					40364				
Black		0.89	0.71	1.13	0.360		1.87	1.59	2.20	<0.001		0.84	0.75	0.95	0.005		0.38	0.31	0.45	<0.001
Asian		1.07	0.92	1.25	0.385		1.38	1.23	1.55	<0.001		0.74	0.68	0.81	<0.001		0.47	0.42	0.52	<0.001
Other ethnicity		1.25	0.97	1.61	0.080		1.20	0.99	1.44	0.062		1.08	0.94	1.25	0.282		0.66	0.55	0.80	<0.001
**Sex**																				
Male	18568	**ref*				22470					41853					41853				
Female		1.09	1.00	1.18	0.039		0.99	0.94	1.04	0.688		0.90	0.86	0.93	<0.001		1.22	1.17	1.28	<0.001
**Age**																				
20-29 years	18568	**ref*				22470					41853					41853				
30-39 years		0.74	0.65	0.85	<0.001		0.68	0.62	0.75	<0.001		1.04	0.96	1.12	0.380		1.07	0.97	1.18	0.192
40-49 years		0.68	0.60	0.77	<0.001		0.69	0.63	0.75	<0.001		0.87	0.81	0.94	<0.001		1.40	1.28	1.54	<0.001
50-59 years		0.76	0.67	0.87	<0.001		0.77	0.71	0.84	<0.001		0.63	0.59	0.68	<0.001		1.58	1.45	1.74	<0.001
60+ years		0.70	0.59	0.84	<0.001		0.88	0.79	0.99	0.031		0.45	0.42	0.48	<0.001		1.69	1.54	1.84	<0.001

Participants from wave 5 (2013-2015) of Understanding Society with data on educational attainment, demographics, and physical activity
Sample size was not restricted and represents total available sample
*Active travel to work: non-active/active; Occupational: non-physical/physical; Leisure-time: <weekly/ ≥ 1x weekly*reference groups*
OR= Odds Ratio, CI= 95% Confidence intervals

Figure S1. Flow diagram of analyses in Understanding Society

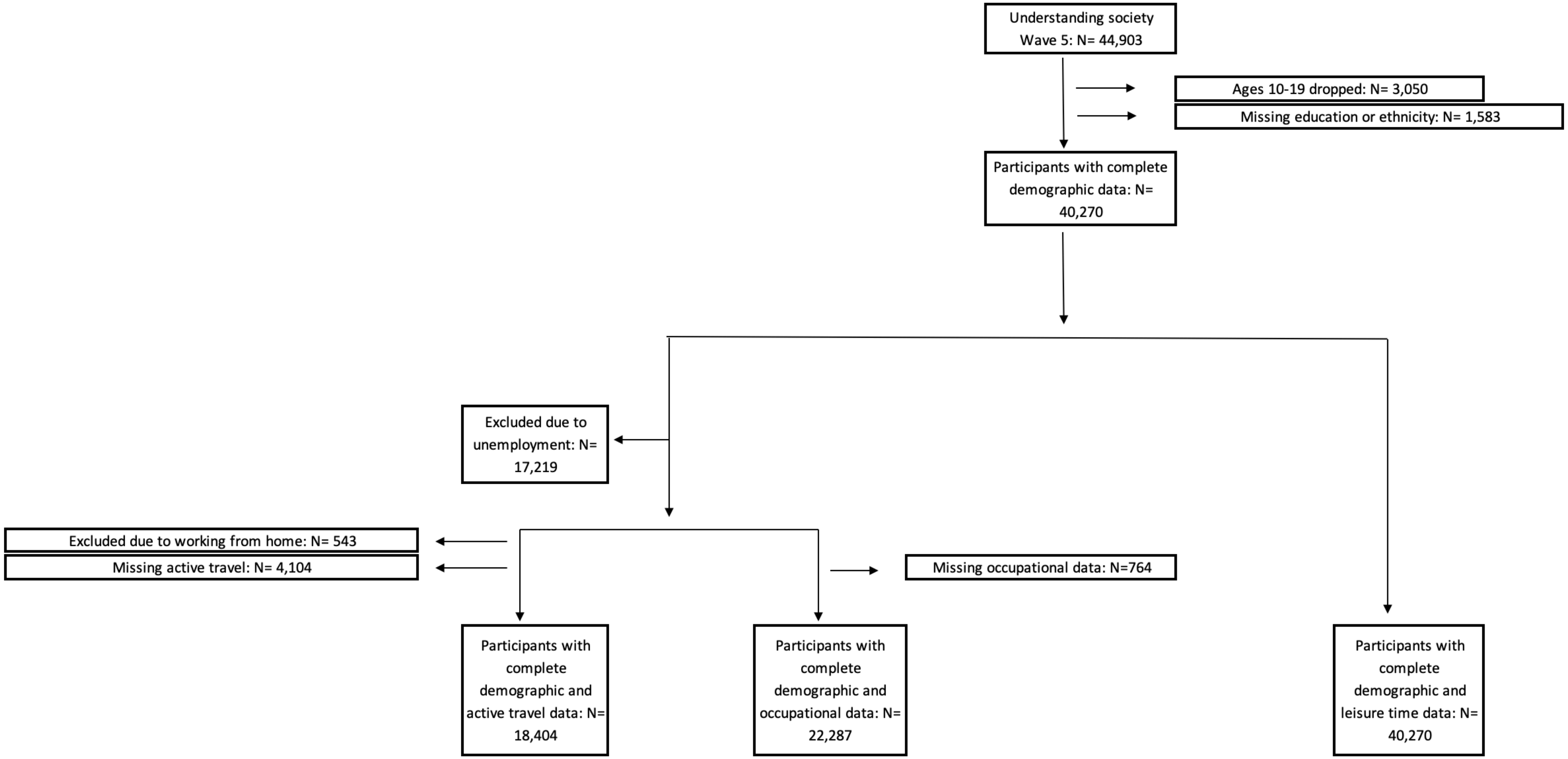

There was no missing data for sex and age demographics.
Active travel and occupational physical activity only include individuals who are currently employed, due to questionnaire routing.

Reporting checklist for cross sectional study.

Based on the STROBE cross sectional guidelines.

Instructions to authors

Complete this checklist by entering the page numbers from your manuscript where readers will find each of the items listed below.

Your article may not currently address all the items on the checklist. Please modify your text to include the missing information. If you are certain that an item does not apply, please write "n/a" and provide a short explanation.

Upload your completed checklist as an extra file when you submit to a journal.

In your methods section, say that you used the STROBE cross sectionalreporting guidelines, and cite them as:

von Elm E, Altman DG, Egger M, Pocock SJ, Gotzsche PC, Vandenbroucke JP. The Strengthening the Reporting of Observational Studies in Epidemiology (STROBE) Statement: guidelines for reporting observational studies.

		Reporting Item	Page Number
**Title and abstract**			
Title	#1a	Indicate the study's design with a commonly used term in the title or the abstract	1
Abstract	#1b	Provide in the abstract an informative and balanced summary of what was done and what was found	2
**Introduction**			
Background / rationale	#2	Explain the scientific background and rationale for the investigation being reported	4
Objectives	#3	State specific objectives, including any prespecified hypotheses	5
**Methods**			

For peer review only

Study design	#4	Present key elements of study design early in the paper	6/7
Setting	#5	Describe the setting, locations, and relevant dates, including periods of recruitment, exposure, follow-up, and data collection	6
Eligibility criteria	#6a	Give the eligibility criteria, and the sources and methods of selection of participants.	6/7
	#7	Clearly define all outcomes, exposures, predictors, potential confounders, and effect modifiers. Give diagnostic criteria, if applicable	6/7
Data sources / measurement	#8	For each variable of interest give sources of data and details of methods of assessment (measurement). Describe comparability of assessment methods if there is more than one group. Give information separately for for exposed and unexposed groups if applicable.	6/7
Bias	#9	Describe any efforts to address potential sources of bias	7
Study size	#10	Explain how the study size was arrived at	7
Quantitative variables	#11	Explain how quantitative variables were handled in the analyses. If applicable, describe which groupings were chosen, and why	6/7
Statistical methods	#12a	Describe all statistical methods, including those used to control for confounding	7
Statistical methods	#12b	Describe any methods used to examine subgroups and interactions	7
Statistical methods	#12c	Explain how missing data were addressed	7
Statistical methods	#12d	If applicable, describe analytical methods taking account of sampling strategy	6/7
Statistical methods	#12e	Describe any sensitivity analyses	n/a

Results

For peer review only

Participants	#13a	Report numbers of individuals at each stage of study—eg numbers potentially eligible, examined for eligibility, confirmed eligible, included in the study, completing follow-up, and analysed. Give information separately for for exposed and unexposed groups if applicable.	8, 13, 21, 27
Participants	#13b	Give reasons for non-participation at each stage	27
Participants	#13c	Consider use of a flow diagram	27
Descriptive data	#14a	Give characteristics of study participants (eg demographic, clinical, social) and information on exposures and potential confounders. Give information separately for exposed and unexposed groups if applicable.	8, 21
Descriptive data	#14b	Indicate number of participants with missing data for each variable of interest	8, 13, 21
Outcome data	#15	Report numbers of outcome events or summary measures. Give information separately for exposed and unexposed groups if applicable.	8, 13, 21
Main results	#16a	Give unadjusted estimates and, if applicable, confounder-adjusted estimates and their precision (eg, 95% confidence interval). Make clear which confounders were adjusted for and why they were included	14, 22, 23
Main results	#16b	Report category boundaries when continuous variables were categorized	n/a
Main results	#16c	If relevant, consider translating estimates of relative risk into absolute risk for a meaningful time period	n/a
Other analyses	#17	Report other analyses done—e.g., analyses of subgroups and interactions, and sensitivity analyses	n/a
**Discussion**			
Key results	#18	Summarise key results with reference to study objectives	10
Limitations	#19	Discuss limitations of the study, taking into account sources of potential bias or imprecision. Discuss both direction and magnitude of any potential bias.	10/11

For peer review only

Interpretation	#20	Give a cautious overall interpretation considering objectives, limitations, multiplicity of analyses, results from similar studies, and other relevant evidence.	11/12
Generalisability	#21	Discuss the generalisability (external validity) of the study results	10
**Other Information**			
Funding	#22	Give the source of funding and the role of the funders for the present study and, if applicable, for the original study on which the present article is based	20

None The STROBE checklist is distributed under the terms of the Creative Commons Attribution License CC-BY. This checklist can be completed online using https://www.goodreports.org/, a tool made by the EQUATOR Network in collaboration with Penelope.ai

For peer review only

BMJ Open

Educational differentials in key domains of physical activity by ethnicity, age, and sex: a cross-sectional study of over 40,000 participants in The UK Household Longitudinal Study (2013-2015)

Journal:	*BMJ Open*
Manuscript ID	bmjopen-2019-033318.R2
Article Type:	Original research
Date Submitted by the Author:	06-Dec-2019
Complete List of Authors:	Fluharty, Meg; University College London, Centre for Longitudinal Studies<br>Pinto Pereira, Snehal; University College London Institute of Child Health, UCL Research Department of Epidemiology & Public Health<br>Benzeval, Michaela; University of Essex, Institute for Social and Economic Research<br>Hamer, Mark; University College London, Division of Surgery & Interventional Science<br>Jefferis, Barbara ; University College London, Department of Primary Care and Population Health<br>Griffiths, Lucy; Swansea University Medical School, Health Data Research UK, Wales and Northern Ireland<br>Cooper, Rachel; Manchester Metropolitan University, Sport and Exercise Sciences<br>Bann, David; University College London, Centre for Longitudinal Studies
**Primary Subject Heading**:	Epidemiology
Secondary Subject Heading:	Public health
Keywords:	health disparities, EPIDEMIOLOGY, physical activity

SCHOLARONE™
Manuscripts

I, the Submitting Author has the right to grant and does grant on behalf of all authors of the Work (as defined in the below author licence), an exclusive licence and/or a non-exclusive licence for contributions from authors who are: i) UK Crown employees; ii) where BMJ has agreed a CC-BY licence shall apply, and/or iii) in accordance with the terms applicable for US Federal Government officers or employees acting as part of their official duties; on a worldwide, perpetual, irrevocable, royalty-free basis to BMJ Publishing Group Ltd ("BMJ") its licensees and where the relevant Journal is co-owned by BMJ to the co-owners of the Journal, to publish the Work in this journal and any other BMJ products and to exploit all rights, as set out in our licence.

The Submitting Author accepts and understands that any supply made under these terms is made by BMJ to the Submitting Author unless you are acting as an employee on behalf of your employer or a postgraduate student of an affiliated institution which is paying any applicable article publishing charge ("APC") for Open Access articles. Where the Submitting Author wishes to make the Work available on an Open Access basis (and intends to pay the relevant APC), the terms of reuse of such Open Access shall be governed by a Creative Commons licence – details of these licences and which Creative Commons licence will apply to this Work are set out in our licence referred to above.

Other than as permitted in any relevant BMJ Author's Self Archiving Policies, I confirm this Work has not been accepted for publication elsewhere, is not being considered for publication elsewhere and does not duplicate material already published. I confirm all authors consent to publication of this Work and authorise the granting of this licence.

For peer review only

Running Head: Educational differentials in physical activity

Educational differentials in key domains of physical activity by ethnicity, age, and sex: a cross-sectional study of over 40,000 participants in The UK Household Longitudinal Study (2013-2015)

Meg E Fluharty[1], Snehal M Pinto Pereira[2], Michaela Benzeval[3], Mark Hamer[4], Barbara Jefferis[5], Lucy Griffiths[6], Rachel Cooper[7], David Bann[1]

1. Centre for Longitudinal Studies, Institute of Education, University College London
2. UCL Research Department of Epidemiology & Public Health, University College London
3. Institute for Social and Economic Research, University of Essex
4. Division of Surgery and Interventional Science, University College London
5. Department of Primary Care and Population Health, University College London
6. Health Data Research UK, Wales and Northern Ireland, Swansea University Medical School
7. Musculoskeletal Science and Sports Medicine Research Centre, Department of Sport and Exercise Sciences, Faculty of Science and Engineering, Manchester Metropolitan University

Corresponding author Meg Fluharty, Centre for Longitudinal Studies, University College London, 55-59 Gordon Square, London WC1H 0NU, United Kingdom. Email: m.fluharty@ucl.ac.uk

For peer review only - http://bmjopen.bmj.com/site/about/guidelines.xhtml

Abstract

Objectives: To assess whether educational differentials in three key physical activity (PA) domains vary by age, sex and ethnicity

Design: National cross-sectional survey

Setting: UK

Participants: Altogether 40,270 participants, aged 20 years and over, from the UK Household Longitudinal Study with information on education, PA and demographics collected in 2013-2015.

Outcome measures: Participation in active travel (AT), occupational activity (OA) and leisure time physical activity (LTPA) at the time of assessment

Results: Lower educational attainment was associated with higher AT and OA, but lower weekly LTPA activity; these associations were modified by sex, ethnicity, and age. Education-related differences in AT were larger for females—the difference in predicted probability of activity between highest and lowest education groups was -10% in females (95% CI: -11.9, 7.9) and -3% in males (-4.8, -0.4). Education-related differences in OA were larger among males -35% (-36.9, -32.4) than females -17% (-19.4, -15.0). Finally, education-related differences in moderate-to-vigorous LTPA varied by ethnicity; for example, differences were 17% (16.2, 18.7) for White individuals compared with 6% (0.6, 11.6) for Black individuals.

Conclusions: Educational differences in PA vary by domain, and are modified by age, sex, and ethnicity. A better understanding of physically inactive sub-groups may aid development of interventions to both increase activity levels and reduce health inequalities.

For peer review only

Strengths and limitations of this study:

1. This study includes a large nationally representative sample, enabling us to examine the role of ethnicity, sex and age as modifiers of the relationship between educational attainment and physical activity.

2. This study examined activity outcomes across three domains; previous studies investigating associations of physical activity typically use a single physical activity outcome measure, capturing either 'leisure' or 'unspecified' activity

3. All physical activity measures were captured via self-report, which may be subject to recall bias with individuals either over or under reporting their levels of physical activity

4. Missing data may introduce bias, although missing data due to item missingness (as opposed to specific question gating) was low

5 This study identified cross-sectional associations of education with physical activity across key domains; however, further longitudinal evidence is required to provide stronger evidence of causality and investigate the mediators of the observed associations.

For peer review only

Introduction

Physical activity is an important modifiable determinant of health. [1] Leisure time physical activity's (LTPA) benefits are particularly well-documented and include improvements in the musculoskeletal system, maintenance of healthy weight, protection against cardiovascular disease, and reduction in symptoms of depression and anxiety.[1] However, there is a global trend towards high levels of leisure time physical inactivity which is estimated to contribute to ~6-10% of major non-communicable diseases, ~5.3 million deaths annually, [2] and ~$67.5 billion per year in health care expenditure. [3]

Physical activity can be accrued through multiple domains (e.g. active travel, leisure time, occupation, and domestic/housework), which may have differing impacts on health outcomes. [4] For example, LTPA is thought to be beneficial to physical health and wellbeing, while labour-intense occupations may increase risk of musculoskeletal strain. [5 6] Therefore, examining these different domains may provide evidence to help inform where possible interventions could be targeted. Understanding what is driving differences in activity participation overall, as well as in different domains of physical activity, may also help to identify which forms of activity could be intervened on to reduce socioeconomic disparities in health.

Recent reviews find evidence of socioeconomic disparities in LTPA in high-income countries [7] that have persisted across recent decades. [8] Additionally, lower education has been shown to be associated with higher risk of future declines in LTPA. [9 10] Alongside indicators of socioeconomic position, a number of other sociodemographic factors, including ethnicity, sex, and age have been shown to be associated with physical activity. [11] For example, differences in the levels of PA participation have been reported across ethnic groups in the UK, with those of 'mixed' ethnicity having the highest prevalence of LTPA, [12 13] and South Asians the lowest. [13 14] Numerous factors may explain these differences including personal, socioeconomic, cultural, and environmental factors. [12 13] Alongside ethnic differences, physical activity levels have been found to be lower for women than men and for older than younger adults. [15]

Educational disparities in physical activity may arise though a number of routes including due to differences in knowledge of the health impacts of LTPA, material pathways (such as low income affecting the affordability of activity participation), and potentially due to selection into neighbourhoods which differ in their suitability for outdoor physical activity. [16 17] For example, lower education may lead to lower income and wealth, and thus a greater likelihood of residing in more disadvantaged areas. Educational differences in physical activity participation may also be modified

For peer review only - http://bmjopen.bmj.com/site/about/guidelines.xhtml

by ethnicity, age, and sex. [4 18] For example, manual occupations that men are more likely employed in are usually more physically demanding than equivalent roles undertaken by women; [4 19] this contrasts with the lower participation in leisure-time activities often observed among men with lower levels of education. [20] Moreover, evidence from the US has indicated education-related disparities across multiple activity domains. [4] However, these associations have not yet been investigated within the UK. Previous studies that have investigated associations of different indicators of socioeconomic position, including education, with physical activity outcomes are limited by only investigating one specific domain, [21 22] or use population samples from specific regions within the UK. [23 24] Thus, important gaps remain in our understanding of the nature of socioeconomic inequalities in physical activity outcomes. These are important to fill given their purported mediating role in socioeconomic inequalities in many important health outcomes including premature mortality. [25]

We sought to address the above-mentioned gaps in the literature by investigating educational disparities in physical activity across active travel, leisure, and occupational domains. Additionally, we aimed to examine if associations between education and domain specific physical activity were modified by ethnicity, age, and sex. We hypothesised that lower education status would be associated with lower levels of participation in physical activity during leisure time, but higher participation in active travel and occupational activity and that these associations would be modified by ethnicity, age and sex. A large household panel study was used (Understanding Society), which benefits from national representation, oversampling of ethnic minority groups, and detailed measures of domain-specific physical activity.

For peer review only

Methods

Participants

Understanding Society: the UK Household Longitudinal Study (UKHLS) is a nationally and regionally representative study which started in 2009 aiming to recruit individuals in 40,000 households.[26] Initial selection of addresses for inclusion of the general population (GP) of the study was via a stratified, clustered, equal probability design.[27] UKHLS ensures proper representation from a range of geographical areas, taking into account socioeconomic and ethnic compositions of neighbourhoods [28], including an ethnic minority boost sample (EMB) to achieve target samples in each minority group.[29] Additionally UKHLS incorporated samples from existing British and Northern Ireland research panels (BHPS/NIHPS) at wave 2; detailed information is included in the sampling design report.[27] The study annually samples all individuals in the household over the age of 10. Additionally, sample members are followed when they leave the household, and new individuals join the study as they become part of an existing study member's household. Information is collected from participants on a range of information including wellbeing, health, home, family and employment. Detailed study information and sampling methodology can be found elsewhere. [26] Ethical approval was approved for all waves by the University of Essex Ethnics Committee, and all participants gave written consent for use of their anonymised survey information.

The sample for our analysis includes adult (20 years or over) responders who took part in Wave 5 (2013-2015) and responded to demographic and physical activity questions via face-to-face computer-assisted personal interview. A total sample of 28,571 households were issued to field for wave 5, and of eligible adults: 85% GP, 75% EMB, and 88% BHPS/NIHPS samples were fully productive. Response rates were lower for men and those of younger ages; detailed information on wave 5 is included in a technical report. [30] Wave 5 was chosen as this was the most recent wave of data collection including physical activity questions, more recent sweeps have not included physical activity assessments.

Those with missing demographic and education data yet valid outcome data were excluded from analysis (N= 1,583); analytical samples for active travel, occupational, and leisure were N= 18,404, N= 22,287 and N= 40,270 respectively. The differences in sample sizes by outcome was largely due to routing - only employed individuals were asked about occupational activity or active travel. A flow diagram (Supplementary Figure S1) displays the final sample size for each outcome.

Patient and public involvement

For peer review only

This study used publicly available secondary data from the UK Data Service (https://www.ukdataservice.ac.uk/). Patients and the public were not involved.

Measures

Domain specific physical activity

Active travel was measured in currently employed individuals and those not working from home via the question '*how do you usually get to your place of work?'* Responses were collapsed into a binary variable of 'non-active' (car, bus, or train/metro) or 'active' (walking or cycling). Occupational physical activity was measured by asking participants whether their job was mainly physical or not (categorised as 'not physical' and 'physical'). Finally, LTPA variables were created from participant responses to the 'Taking part Survey' (source: Department for Culture, Media and Sport), a survey on engagement with a range of different leisure time activities including sport".[31] This includes an assessment of how often they participated in a series of prelisted sports and activities. Sports were then grouped into two categories based on their average metabolic equivalent of task (MET), those with METs of ≥3 were categorised as moderate-to-vigorous and METs 1.5-2.9 as light, using cut offs widely used in previous physical activity studies. [32] Frequency of participation in each MET-group was categorised as weekly or non-weekly.

Socio demographics

Highest educational attainment was self-reported and categorised into three groups: 'degree or higher (university level education typically undertaken after age 18), 'school diploma/ other qualification' (e.g. A levels and vocational diplomas, education to age 18) and 'GCSEs and below' (education to age 16—compulsory schooling age).

Ethnicity was self-reported and responses were collapsed into 'White', 'Black', 'Asian' and 'other' . These broad ethnic groupings include minority groups—'White' includes all white minorities such as Irish and Polish, Black includes Black-African and Black-Caribbean, while those of smaller sample sizes such as Arab and mixed-ethnicity were included in 'other'. Age at the time of interview was categorised into ten year age groups (from ages 20-60). Older adults were grouped from >60 years, and those below 20 (N= 3,050) were excluded from the analysis to ensure comparable sample sizes in the higher education groups—alternative groupings did not substantially affect the results (data available upon request).

Statistical analysis

For peer review only - http://bmjopen.bmj.com/site/about/guidelines.xhtml

We first cross-tabulated educational attainment by age, sex, and ethnicity. Next, logistic regression analyses were conducted to examine associations of education, sex, age, and ethnicity with physical activity in each domain. Analyses were assessed before and after mutual adjustment for each demographic variable. Finally, to examine possible effect modification of the associations between education and physical activity in each domain, we included two-way interaction terms (education x ethnicity; education x sex; education x age) in addition to the relevant first order terms in the same model. Analyses were weighted according to sample design and attrition to reduce bias by under-coverage, sampling, or non-response. [26 33] Associations were presented as odds ratios (OR) with 95% confidence intervals (CI), while tests of moderation were presented as absolute differences in the predicted probability of each physical activity outcome comparing the highest and lowest education groups. All analyses were conducted using Stata, version 15.0 (StataCorp LP, College Station, Texas).

Results

Ethnicity, sex, and age were each independently associated with educational attainment (see Supplementary Table S1) and with physical activity in each domain (p< 0.001; see Table 1). Lower educational attainment was associated with higher active travel and occupational physical activity, but lower weekly light and moderate LTPA. White ethnicity was associated with higher LTPA and active travel, but less occupational physical activity. Additionally, males participated in more moderate-to-vigorous LTPA and occupational activity, but lower active travel than females. Finally, younger age was associated with higher active travel, occupational physical activity, and moderate-to-vigorous LTPA, but less light LTPA (see Table 2).

Active travel to work

Active travel was lowest amongst individuals who were highly-educated, older, and male; there was little evidence for a strong association with ethnicity (See Table 2). The magnitude of education-related disparities were largest among females (education x sex P<0.001) and Black individuals (education x ethnicity P= 0.038) (See Figure 1 & Supplementary Tables S2-4). For example, the estimated difference in the probability of using active travel in the highest versus the lowest educational group was -10% (95% CI: -11.9, 7.9) amongst females and -3% (-4.8, -0.4) among males (see Supplementary tables S2-S4). Results for this domain, and all others, were similar when restricting to those with valid demographic and physical activity data, or when not making this restriction (Supplementary tables S5-6).

Occupational activity

For peer review only

For peer review only - http://bmjopen.bmj.com/site/about/guidelines.xhtml

Physically active occupations were less commonly reported amongst individuals who were highly-educated, white, and aged over 20-29; there was no evidence for association with sex (see Table 2). The magnitude of education-related disparities were largest among males (education x sex P <0.001), and those aged 30-39 (education x age P= 0.001) (See Figure 2 and Supplementary Tables S2-4). For example, the estimated difference in the probability of a physical occupation in the highest versus lowest educational group was -35% (-36.9, -32.4) for males and -17% (-19.4, -15.0) for females (see Supplementary tables S2-S4).

Moderate-to-vigorous and light LTPA

Greater levels of participation in both weekly light and moderate-to-vigorous LTPA were reported amongst individuals who were highly educated and White. Males and younger adults were also more likely to report participation in moderate-to-vigorous LTPA, whereas females and older adult were more likely to report participation in light LTPA. (See Table 2).

The magnitude of education-related disparities in weekly moderate-to-vigorous LTPA was largest among White and Asian individuals (education x ethnicity P= 0.001), and those aged 40-49 and 50-59 (education x age P= 0.008) (See Figure 3 and Supplementary Tables S2-4). For example, the estimated probability of weekly moderate-to-vigorous LTPA in the highest versus lowest educational group was 17% (16.2, 18.7) for white individuals, compared with 6% (0.6, 11.6) for Black individuals, 16% (12.8, 19.1) for Asian individuals, and 13% (6.0, 19.5) for those of other ethnicity (see Supplementary tables S2-S4).

The magnitude of education-related disparities in weekly light LTPA was largest among females (education x sex P< 0.001) and individuals aged 60+ (education x age P< 0.001); there was little evidence for associations with ethnicity (See Figure 4 and Supplementary Tables S2-4). For example, the estimated probability of weekly light leisure time activity in the highest versus lowest educational group was 13% (11.4, 15.3) for those aged 60+ compared with 8% (5.9, 10.6) for those ages 50-59, 3% (1.3, 5.6) for those 40-49, 3% (0.9, 5.4) for those 30-39, and 2% (-.04, 4.7) for those aged 20-29 (see Supplementary tables S2-S4).

For peer review only - http://bmjopen.bmj.com/site/about/guidelines.xhtml

Discussion

Main findings and interpretations

In a large nationally representative dataset, educational attainment was associated with physical activity across three key domains; individuals with higher education were less likely to engage in active travel and occupational physical activity, but were more likely to engage in LTPA. These associations were modified by ethnicity, age, and sex. For active travel, educational disparities were largest among females and Black individuals. For occupational physical activity disparities were largest among males and those aged 30-39. For moderate-to-vigorous LTPA, educational disparities were largest among White and Asian individuals and those aged 40-49 and 50-59. Finally, for light LTPA, disparities were largest among females and those aged 60+.

Our findings may be explained by disparities in factors which affect physical activity levels such as health status, [34] environment, [35 36] cultural preferences, [37 38] financial resources, [25 39 40], perceived safety, [41 42] and domestic requirements. [43 44] These factors may differ between sociodemographic groups within education levels, resulting in differing magnitudes of disparities observed. The pathways involved may differ across each activity domain. For example, areas perceived as unsafe may result in reduced use for either travel and/or for leisure-time purposes. [45 46] Similarly, affordability of facilities (e.g. gym memberships) and other assets (e.g. cars) may yield different opportunities for participation in physical activity. English proficiency and work experience may also create unequal occupational opportunities. [45 46] While access times [43 44] and cultural expectations [38] may additionally contribute to differences in leisure-time participation.

Strengths and Limitations

Strengths of this study include a large nationally representative sample, enabling us to identify the previously seldom-examined role of ethnicity as a modifier of the relationship between educational attainment and physical activity across different domains. We also examined activity outcomes across three domains; previous studies investigating associations of physical activity typically use a single physical activity outcome measure, capturing either 'leisure' or 'unspecified' activity. [4 10]

There are also a number of limitations to consider. First, while we obtained information across multiple domains, we lack detailed information on activity duration. However, the LTPA measures used followed expected patterns for these leisure time categories by sex and age. [47] We also did not consider perceptions of the local environment including safety which may affect physical activity. [41 42] Second, we did not capture physical housework as a domain, which includes domestic and

For peer review only

cleaning tasks, gardening, and do-it-yourself (DIY). Third, all physical-activity measures were captured via self-report; while this is needed to investigate domain-specific activity, it may be subject to recall bias with individuals' either over or under reporting their levels of physical activity. [48] For example, previous evidence has shown that males and those of lower education were more likely to overestimate their physical activity levels than females and those with higher education, respectively. [49] Differential reporting bias across population sub groups[50] could therefore bias our finding of effect modification. Insofar as objective measurements of physical activity are able to capture domain-specific activity, they may be useful to include in future studies to help verify our findings. Fourth, only working adults could be included in the analyses of the active travel and occupational domains; investigation of multiple types of physical activity among retirees, those currently seeking work, or those unable to work warrants consideration in the future. Fifth, bias may be introduced through excluding missing data and non-responders, although missing data due to item missingness (as opposed to specific question gating) was low and therefore bias unlikely We also used weights to reduce bias caused by under-coverage, sampling, or non-response. [33] Sixth, due to the cross-sectional design we cannot separate out age from birth cohort effects, and so future cross-cohort studies are required to address this. Finally, this study identified cross-sectional associations of education with physical activity across key domains, as well as differences by ethnicity, sex, and age in these associations. While we hypothesised that the primary direction of causality was from education attainment to physical activity outcomes, physical activity may affect educational attainment at younger ages.[51] Further longitudinal analyses may provide stronger evidence on the causal nature of the observed associations and additionally identify the mediators of the disparities observed.

Implications for practice, and policy

Our findings may have important implications for practice and policy. The inequalities in LTPA observed—across both light and moderate-vigorous activity—suggests that policies are required to reduce these inequalities given the multiple anticipated effects on health. Population-level or targeted interventions may be used to reduce the sizeable modification across demographic sub-groups. For example, there was a 13% difference across education levels in the probability of participating in light LTPA amongst those aged 60+ compared with 2-3% of those aged 20-39, suggesting older adults with lower levels of education would benefit most from interventions regarding this domain of physical activity. Furthermore, in line with previous evidence [19] we also found that those of lower educational attainment were more likely to possess physically demanding occupations. This difference is important, if the health consequences of occupational physical

For peer review only - http://bmjopen.bmj.com/site/about/guidelines.xhtml

activity are less favourable or detrimental compared with LTPA. [5 6] Efforts to increase LTPA and its inequality should consider co-occurring differences in occupational activity. Finally, lower participation in active travel was also found in those with higher levels of educational attainment. For example, there was a 10% difference in active travel among high to low educated women compared with 3% in men. Previous evidence has found similar sex differences in cycling to work; however, similar proportions of men and women report leisure-time cycling. [52] Suggested means of increasing active travel include the provision of safe walking and cycling travel routes, accessible bike locks, and changing facilities.

Our findings may have implications for future studies which investigate inequalities in physical activity outcomes. Existing studies typically adjust for the sociodemographic factors we investigated as potential modifiers. Given the evidence for modification that we found, such analyses may provide biased estimates of the magnitude of inequalities that operate in particular population sub-groups.

Conclusions

In summary, we found sex, age, and ethnicity modified associations between educational attainment and multiple physical activity outcomes. Our findings imply there may be unequal access or additional barriers to physical activity across both education and demographic sub-groups. Better understanding the characteristics of physically inactive sub-groups may aid development of tailored interventions to increase activity levels and reduce health inequalities.

For peer review only

Table 1. Physical activity domains by education and demographic characteristics in Understanding Society (2013-2015)

Demographic	Physical activity domain															
	Active transportation				Occupational				Leisure-time							
									Moderate-to-vigorous				Light			
	Non-active		Active		Not physical		Physical		< Weekly		≥ 1x weekly		< Weekly		≥ 1x weekly	
	N	*%*	*N*	*%*	*N*	*%*	*N*	*%*	*N*	*%*	*N*	*%*	*N*	*%*	*N*	*%*
**Educational attainment**																
GCSEs and lower	3639	81.1	846	18.9	1471	27.3	3920	72.7	11048	79.8	2798	20.2	11482	82.9	2364	17.1
School diploma	4547	83.4	906	16.6	2200	33.4	4383	66.6	8857	71.3	3570	28.7	9910	79.8	2517	20.3
Degree/higher	7519	87.5	1078	12.5	5394	51.6	5063	48.4	9358	60.7	6069	39.3	11914	77.2	3513	22.8
P value				<0.001				<0.001				<0.001				<0.001
**Ethnicity**																
White	13397	84.8	2396	15.2	8001	41.7	11199	58.3	23749	68.7	10822	31.3	26892	77.8	7679	22.2
Black	631	86.2	101	13.8	227	27.7	594	72.4	1047	72.3	402	27.7	1308	90.3	141	9.7
Asian	1223	83.9	234	16.1	598	34.1	1158	66.0	2571	74.9	864	25.2	3030	88.2	405	11.8
Other ethnicity	366	81.7	82	18.3	202	37.4	338	62.6	609	67.0	300	33.0	764	84.1	145	16.0
P value				<0.001				<0.001				<0.001				<0.001
**Gender**																
Male	7076	85.4	1215	14.7	4296	40.3	6376	59.8	13305	69.0	5979	31.0	15741	81.6	3543	18.4
Female	8656	84.2	1621	15.8	4780	40.5	7018	59.5	16084	71.3	6485	28.7	17702	78.4	4867	21.6
P value				<0.001				<0.001				<0.001				<0.001
**Age**																
20-29 years	2703	81.0	633	19.0	1235	34.2	2381	65.9	3848	62.9	2272	37.1	5188	84.8	932	15.2
30-39 years	3650	85.2	636	14.8	2171	43.3	2841	56.7	4220	62.1	2579	37.9	5704	83.9	1095	16.1
40-49 years	4511	86.3	718	13.7	2730	43.0	3622	57.0	5526	66.1	2835	33.9	6679	79.9	1682	20.1
50-59 years	3594	84.9	640	15.1	2108	40.2	3137	59.8	5479	72.8	2043	27.2	5855	77.8	1667	22.2
60+ years	1274	85.9	209	14.1	832	37.1	1413	62.9	10316	79.0	2735	21.0	10017	76.8	3034	23.3

P value	<0.001	<0.001	<0.001	<0.001

Participants from wave 5 (2013-2015) of Understanding Society with data on educational attainment, demographics, and physical activity
P value = chi[2,]

For peer review only

Table 2. Mutually adjusted associations of educational attainment and demographic characteristics with domain-specific physical activity outcomes

	Physical Activity															
	**Active travel**				**Occupational**				**Leisure time**							
									**Moderate-to-vigorous**				**Light**			
	N=18,404				*N=22,287*				*N= 40,270*				*N= 40,270*			
	OR	*95% CI*		*p*	*OR*	*95% CI*		*p*	*OR*	*95% CI*		*p*	*OR*	*95% CI*		*p*
**Education**																
GCSEs and lower	**ref*															
School diploma	0.83	0.74	0.93	0.001	0.73	0.67	0.79	<0.001	1.44	1.35	1.53	<0.001	1.33	1.24	1.42	<0.001
Degree/higher	0.59	0.53	0.66	<0.001	0.34	0.31	0.37	<0.001	2.28	2.16	2.42	<0.001	1.58	1.49	1.69	<0.001
**Ethnicity**																
White	**ref*															
Black	0.94	0.74	1.19	0.620	2.19	1.84	2.59	<0.001	0.69	0.61	0.77	<0.001	0.38	0.31	0.46	<0.001
Asian	1.10	0.95	1.29	0.211	1.56	1.38	1.75	<0.001	0.59	0.54	0.64	<0.001	0.51	0.46	0.57	<0.001
Other ethnicity	1.31	1.01	1.69	0.041	1.40	1.16	1.70	0.001	0.82	0.71	0.95	0.008	0.69	0.57	0.83	<0.001
**Sex**																
Male	**ref*															
Female	1.11	1.02	1.21	0.016	1.04	0.99	1.10	0.150	0.84	0.81	0.88	<0.001	1.20	1.14	1.26	<0.001
**Age**																
20-29 years	**ref*															
30-39 years	0.77	0.67	0.88	<0.001	0.71	0.65	0.79	<0.001	0.96	0.88	1.04	0.287	1.02	0.92	1.13	0.676
40-49 years	0.67	0.59	0.76	<0.001	0.68	0.62	0.75	<0.001	0.81	0.75	0.88	<0.001	1.36	1.24	1.50	<0.001
50-59 years	0.74	0.64	0.84	<0.001	0.74	0.68	0.82	<0.001	0.59	0.55	0.64	<0.001	1.52	1.39	1.67	<0.001
60+ years	0.66	0.55	0.79	<0.001	0.83	0.74	0.94	0.003	0.44	0.41	0.48	<0.001	1.64	1.50	1.80	<0.001

Participants from wave 5 (2013-2015) of Understanding Society with valid data on educational attainment, demographics, and physical activity
Samples were restricted to those with valid demographic and physical activity data
Active travel to work: non-active/active; Occupational: non-physical/physical; Leisure-time: <weekly/ ≥ 1x weekly
Analyses are mutually adjusted for education, age, sex, and ethnicity
OR= Odds Ratio, CI= 95% Confidence intervals
**reference group*

References

1. Warburton DE, Nicol CW, Bredin SS. Health benefits of physical activity: the evidence. *CMAJ* 2006;174(6):801-9. doi: 10.1503/cmaj.051351
2. Kohl HW, 3rd, Craig CL, Lambert EV, et al. The pandemic of physical inactivity: global action for public health. *Lancet* 2012;380(9838):294-305. doi: 10.1016/S0140-6736(12)60898-8
3. Ding D, Lawson KD, Kolbe-Alexander TL, et al. The economic burden of physical inactivity: a global analysis of major non-communicable diseases. *Lancet* 2016;388(10051):1311-24. doi: 10.1016/S0140-6736(16)30383-X
4. Scholes S, Bann D. Education-related disparities in reported physical activity during leisure-time, active transportation, and work among US adults: repeated cross-sectional analysis from the National Health and Nutrition Examination Surveys, 2007 to 2016. *Bmc Public Health* 2018;18(1):926. doi: 10.1186/s12889-018-5857-z
5. Holtermann A, Hansen JV, Burr H, et al. The health paradox of occupational and leisure-time physical activity. *Br J Sports Med* 2012;46(4):291-5. doi: 10.1136/bjsm.2010.079582 [published Online First: 2011/04/05]
6. Holtermann A, Krause N, van der Beek AJ, et al. The physical activity paradox: six reasons why occupational physical activity (OPA) does not confer the cardiovascular health benefits that leisure time physical activity does. *Br J Sports Med* 2018;52(3):149-50. doi: 10.1136/bjsports-2017-097965 [published Online First: 2017/08/12]
7. Elhakeem A, Cooper R, Bann D, et al. Childhood socioeconomic position and adult leisure-time physical activity: a systematic review. *Int J Behav Nutr Phys Act* 2015;12:92. doi: 10.1186/s12966-015-0250-0
8. Bleich SN, Jarlenski MP, Bell CN, et al. Health inequalities: trends, progress, and policy. *Annu Rev Public Health* 2012;33:7-40. doi: 10.1146/annurev-publhealth-031811-124658
9. Droomers M, Schrijvers CT, Mackenbach JP. Educational level and decreases in leisure time physical activity: predictors from the longitudinal GLOBE study. *J Epidemiol Community Health* 2001;55(8):562-8. [published Online First: 2001/07/13]
10. Harper S, Lynch J. Trends in socioeconomic inequalities in adult health behaviors among US states, 1990-2004. *Public Health Rep* 2007;122(2):177-89. doi: Doi 10.1177/003335490712200207
11. Bann D, Johnson W, Li L, et al. Socioeconomic inequalities in childhood and adolescent body-mass index, weight, and height from 1953 to 2015: an analysis of four longitudinal, observational, British birth cohort studies. *Lancet Public Health* 2018;3(4):E194-E203. doi: 10.1016/S2468-2667(18)30045-8
12. Hayes L, White M, Unwin N, et al. Patterns of physical activity and relationship with risk markers for cardiovascular disease and diabetes in Indian, Pakistani, Bangladeshi and European adults in a UK population. *J Public Health Med* 2002;24(3):170-8.
13. Obesity and ethnicity. In: Health SfP, ed.: National Obesity Observatory, 2011.
14. Fischbacher CM, Hunt S, Alexander L. How physically active are South Asians in the United Kingdom? A literature review. *J Public Health (Oxf)* 2004;26(3):250-8. doi: 10.1093/pubmed/fdh158
15. Guthold R, Stevens GA, Riley LM, et al. Worldwide trends in insufficient physical activity from 2001 to 2016: a pooled analysis of 358 population-based surveys with 1.9 million participants. *Lancet Glob Health* 2018;6(10):e1077-e86. doi: 10.1016/S2214-109X(18)30357-7 [published Online First: 2018/09/09]
16. Bécares L. The Ethnic Density effect on the Health of Ethnic Minority People in the United Kingdom: a Study of Hypothesised Pathways. London: University College London, 2009.

For peer review only

17. Pickett KE, Shaw RJ, Atkin K, et al. Ethnic density effects on maternal and infant health in the Millennium Cohort Study. *Soc Sci Med* 2009;69(10):1476-83. doi: 10.1016/j.socscimed.2009.08.031
18. Saffer H, Dave D, Grossman M, et al. Racial, Ethnic, and Gender Differences in Physical Activity. *J Hum Cap* 2013;7(4):378-410. doi: 10.1086/671200
19. He XZ, Baker DW. Differences in leisure-time, household, and work-related physical activity by race, ethnicity, and education. *J Gen Intern Med* 2005;20(3):259-66. doi: 10.1111/j.1525-1497.2005.40198.x
20. Evandrou M, Falkingham J, Feng ZX, et al. Ethnic inequalities in limiting health and self-reported health in later life revisited. *J Epidemiol Commun H* 2016;70(7):653-62. doi: 10.1136/jech-2015-206074
21. Farrell L, Hollingsworth B, Propper C, et al. The socioeconomic gradient in physical inactivity: Evidence from one million adults in England. *Soc Sci Med* 2014;123:55-63. doi: 10.1016/j.socscimed.2014.10.039
22. Stalsberg R, Pedersen AV. Effects of socioeconomic status on the physical activity in adolescents: a systematic review of the evidence. *Scand J Med Sci Sports* 2010;20(3):368-83. doi: 10.1111/j.1600-0838.2009.01047.x [published Online First: 2010/02/09]
23. Berger U, Der G, Mutrie N, et al. The impact of retirement on physical activity. *Ageing Soc* 2005;25:181-95. doi: 10.1017/S0144686x04002739
24. Mutrie N, Hannah MK. Some work hard while others play hard. *International Journal of Health Promotion and Education* 2013;42(2):109-17. doi: 10.1080/14635240.2004.10708024
25. Stringhini S, Sabia S, Shipley M, et al. Association of socioeconomic position with health behaviors and mortality. *JAMA* 2010;303(12):1159-66. doi: 10.1001/jama.2010.297 [published Online First: 2010/03/25]
26. Knies G. Unverstanding Society: The UK Household Longitundal Study: Waves 1-8 User Guide Essex: University of Essex; 2018 [Available from: https://www.understandingsociety.ac.uk/sites/default/files/downloads/documentation/mainstage/user-guides/mainstage-user-guide.pdf]
27. Lynn P. Sample Design for Understanding Society. Understanding Society Working Paper Series Institute for Social and Economic Research, University of Essex 2009. [Available from: https://www.understandingsociety.ac.uk/sites/default/files/downloads/working-papers/2009-01.pdf]
28. Peterson J, Rabe B. Understanding Society – a geographical profile of respondents University of Essex [Available from: https://www.understandingsociety.ac.uk/sites/default/files/downloads/working-papers/2013-01.pdf]
29. Berthoud R, Fumagalli, L., Lynn, P., Platt, L. . Design of the Understanding Society Ethnic Minority Boost Sample Understanding Society Working Paper Series Institute for Social and Economic Research, University of Essex 2009. [Available from: https://www.understandingsociety.ac.uk/sites/default/files/downloads/working-papers/2009-02.pdf]
30. Jessop C. UK Household Longitudinal Study Wave 5 Technical Report. London: NatCen Social Research, 2015. [Available from: http://doc.ukdataservice.ac.uk/doc/6676/mrdoc/pdf/6676_wave5_technical_report.pdf]
31. Department for Digital C, Media & Sport. Taking Part Survey 2016 [Available from: https://www.gov.uk/guidance/taking-part-survey#the-taking-part-survey.
32. Ainsworth BE, Haskell WL, Herrmann SD, et al. 2011 Compendium of Physical Activities: a second update of codes and MET values. *Med Sci Sports Exerc* 2011;43(8):1575-81. doi: 10.1249/MSS.0b013e31821ece12 [published Online First: 2011/06/18]
33. Lynn P, Kaminska O. Weighting strategy for Understanding Society. Understanding Society Working Paper Series: Institute for Social and Economic Research, University of Essex. [Available from:

For peer review only - http://bmjopen.bmj.com/site/about/guidelines.xhtml

https://www.understandingsociety.ac.uk/sites/default/files/downloads/working-papers/2010-05.pdf]
34. Bauman AE, Reis RS, Sallis JF, et al. Correlates of physical activity: why are some people physically active and others not? *Lancet* 2012;380(9838):258-71. doi: 10.1016/S0140-6736(12)60735-1 [published Online First: 2012/07/24]
35. . In: CABE, ed. Community green: using local spaces to tackle inequality and improve health. London.
36. Bird S, Kurowski W, Feldman S, et al. The influence of the built environment and other factors on the physical activity of older women from different ethnic communities. *J Women Aging* 2009;21(1):33-47. doi: 10.1080/08952840802633669
37. Rojas A, Storch EA. Psychological complications of obesity. *Pediatr Ann* 2010;39(3):174-80. doi: 10.3928/00904481-20100223-07
38. Long J, Hylton K, Spracklen K, et al. Long J, Hylton K, Spracklen K, Ratna A, Bailey S. Sporting Equals. A Systematic Review of the Literature on Black and Minority Ethnic Communities on Sport and Physical Recreation. Leeds: Carnegie Institute, 2009.
39. Finger JD, Tylleskar T, Lampert T, et al. Physical activity patterns and socioeconomic position: the German National Health Interview and Examination Survey 1998 (GNHIES98). *Bmc Public Health* 2012;12:1079. doi: 10.1186/1471-2458-12-1079 [published Online First: 2012/12/18]
40. Popham F, Mitchell R. Relation of employment status to socioeconomic position and physical activity types. *Prev Med* 2007;45(2-3):182-8. doi: 10.1016/j.ypmed.2007.06.012 [published Online First: 2007/07/31]
41. Panter JR, Jones AP, van Sluijs EM. Environmental determinants of active travel in youth: a review and framework for future research. *Int J Behav Nutr Phys Act* 2008;5:34. doi: 10.1186/1479-5868-5-34 [published Online First: 2008/06/25]
42. Hooker SP, Wilson DK, Griffin SF, et al. Perceptions of environmental supports for physical activity in African American and white adults in a rural county in South Carolina. *Prev Chronic Dis* 2005;2(4):A11. [published Online First: 2005/09/17]
43. Telama R, Yang X, Viikari J, et al. Physical activity from childhood to adulthood: a 21-year tracking study. *Am J Prev Med* 2005;28(3):267-73. doi: 10.1016/j.amepre.2004.12.003
44. Telama R. Tracking of physical activity from childhood to adulthood: a review. *Obes Facts* 2009;2(3):187-95. doi: 10.1159/000222244
45. Hispanics in the U.S. Labor Market: Selected Research. *Hispanics in the US Labor Market: Selected Research* 2014:1-146.
46. Employment by occupation. In: Department for Business EIS, Gov.uk, ed. England, 2018.
47. Caspersen CJ, Pereira MA, Curran KM. Changes in physical activity patterns in the United States, by sex and cross-sectional age. *Med Sci Sports Exerc* 2000;32(9):1601-9. [published Online First: 2000/09/20]
48. Mann CJ. Observational research methods. Research design II: cohort, cross sectional, and case-control studies. *Emerg Med J* 2003;20(1):54-60. [published Online First: 2003/01/21]
49. Watkinson C, van Sluijs EM, Sutton S, et al. Overestimation of physical activity level is associated with lower BMI: a cross-sectional analysis. *Int J Behav Nutr Phys Act* 2010;7:68. doi: 10.1186/1479-5868-7-68 [published Online First: 2010/09/22]
50. Kapteyn A, Banks J, Hamer M, et al. What they say and what they do: comparing physical activity across the USA, England and the Netherlands. *J Epidemiol Community Health* 2018;72(6):471-76. doi: 10.1136/jech-2017-209703 [published Online First: 2018/04/13]
51. . In: Kohl HW, III, Cook HD, eds. Educating the Student Body: Taking Physical Activity and Physical Education to School. Washington (DC)2013.
52. Heesch KC, Sahlqvist S, Garrard J. Gender differences in recreational and transport cycling: a cross-sectional mixed-methods comparison of cycling patterns, motivators, and constraints. *Int J Behav Nutr Phys Act* 2012;9:106. doi: 10.1186/1479-5868-9-106 [published Online First: 2012/09/11]

For peer review only - http://bmjopen.bmj.com/site/about/guidelines.xhtml

Figures 1-4 Legend

Data: Understanding Society, Wave 5. Dotted line represents reference group (GCSE & lower). Estimates are derived from separate logistic regression models of each binary physical activity outcome including a two-way interaction term (demographic x ethnicity); P values indicate demographic x education.

Funding

This project is part of a collaborative research programme entitled 'Cohorts and Longitudinal Studies Enhancement Resources' (CLOSER) funded by the ESRC (http://www.esrc.ac.uk) (ES/K000357/1). DB and MF are also supported by the Academy of Medical Sciences/the Wellcome Trust "Springboard Health of the public in 2040" Award (HOP001\1025). SPP is funded by a UK Medical Research Council Career Development Award (MR/P020372/1). MB is funded by ESRC ES/N00812X/1. Understanding Society is funded by the Economic and Social Research Council (ES/N00812X/1).

Competing interests

None to declare.

Author contributions

MF, DB, SPP and RC were involved in the conception and design of the study, MF conducted the analyses and drafted the manuscript, and MF, SPP, MB, MH, BJ, LG, RC and DB have revised the manuscript and approved for publication.

Data availability statement

Data for this study, Understanding Society, was accessed through the UK Data Service (https://www.ukdataservice.ac.uk).

Licence statement

I, the Submitting Author has the right to grant and does grant on behalf of all authors of the Work (as defined in the below author licence), an exclusive licence and/or a non-exclusive licence for contributions from authors who are: i) UK Crown employees; ii) where BMJ has agreed a CC-BY licence shall apply, and/or iii) in accordance with the terms applicable for US Federal

For peer review only

Government officers or employees acting as part of their official duties; on a worldwide, perpetual, irrevocable, royalty-free basis to BMJ Publishing Group Ltd ("BMJ") its licensees and where the relevant Journal is co-owned by BMJ to the co-owners of the Journal, to publish the Work in BMJ Open and any other BMJ products and to exploit all rights, as set out in our licence.

The Submitting Author accepts and understands that any supply made under these terms is made by BMJ to the Submitting Author unless you are acting as an employee on behalf of your employer or a postgraduate student of an affiliated institution which is paying any applicable article publishing charge ("APC") for Open Access articles. Where the Submitting Author wishes to make the Work available on an Open Access basis (and intends to pay the relevant APC), the terms of reuse of such Open Access shall be governed by a Creative Commons licence – details of these licences and which Creative Commons licence will apply to this Work are set out in our licence referred to above.

For peer review only

Figure 1. Educational differences in active travel by sex, ethnicity, and age

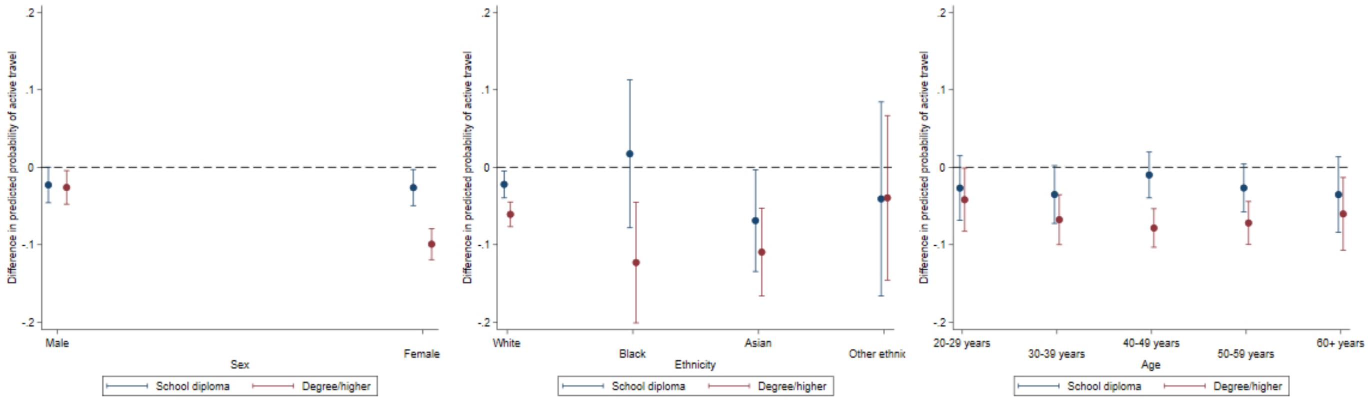

Figure 2. Educational differences in occupational physical activity by sex, ethnicity, and age

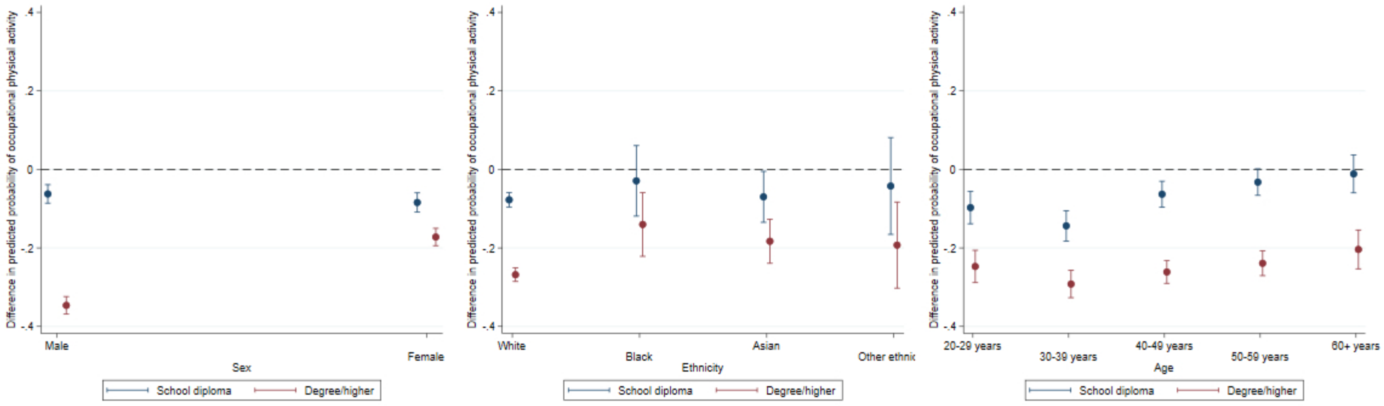

For peer review only

Figure 3. Educational differences in moderate to vigorous intesity activities by sex, ethnicity, and age

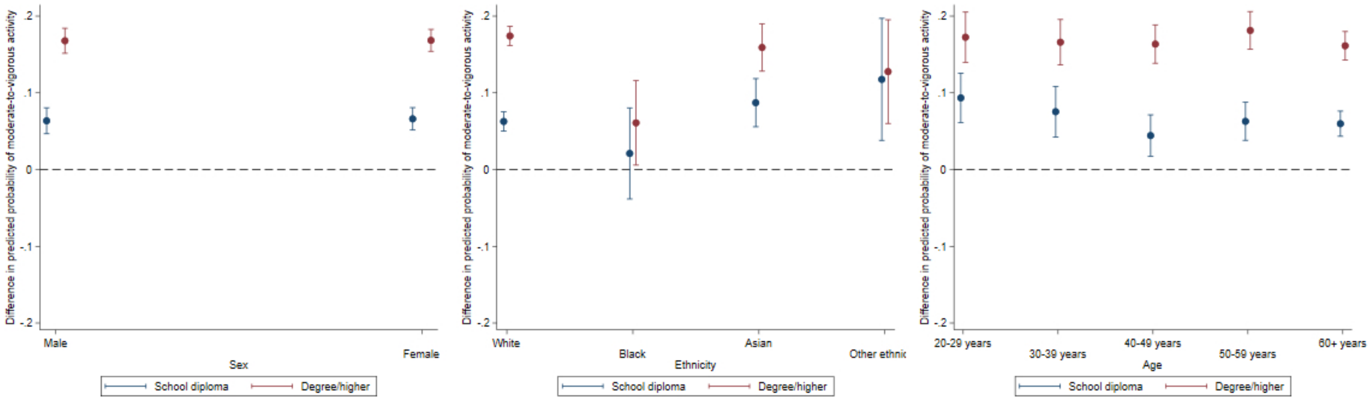

For peer review only

Figure 4. Educational differences in light intesity activities by sex, ethnicity, and age

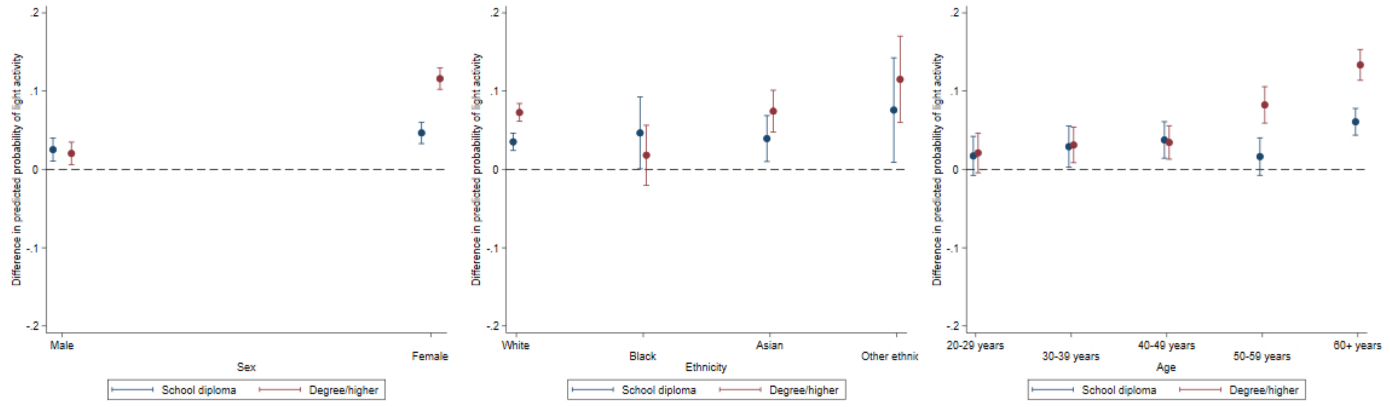

For peer review only

Table S1. Ethnicity, sex and age by highest educational attainment in Understanding Society (2013-2015)

Demographics	Highest educational attainment					
	GCSEs and lower		School diploma & other qualifications		Degree/higher	
	N	*(%)*	N	*(%)*	N	*(%)*
**Ethnicity**						
White	11638	33.7	10424	30.2	12424	35.9
Black	363	25.1	400	27.6	685	47.3
Asian	1106	32.2	877	25.5	1445	42.1
Other ethnicity	201	22.1	235	25.9	472	51.9
Missing	538	36.1	491	33	401	26.9
P value	<0.001					
**Sex**						
Male	5888	30.5	6388	33.1	6927	35.9
Female	7958	35.3	6039	26.8	8500	37.7
P value	<0.001					
**Age**						
20-29 years	1462	23.9	2363	38.6	2271	37.1
30-39 years	1653	24.3	1776	26.1	3345	49.2
40-49 years	2433	29.1	2339	28	3570	42.7
50-59 years	2455	32.6	2221	29.5	2822	37.5
60+	5843	44.8	3728	28.6	3419	26.2
P value	<0.001					

Participants from wave 5 (2013-2015) of Understanding Society with data on educational attainment, demographics, and physical activity
Missing = missing data; not displayed where data was available in complete.
P value = chi^{2},

For peer review only

Table S2. Estimated difference in predicted probability in educational differences in physical activity by age

**Physical activity**	**20-29**				**30-39**				**40-49**				**50-59**				**60+**				education X age
	Coef.	*95% CI*		*p*	*Coef.*	*95% CI*		*p*	*Coef.*	*95% CI*		*p*	*Coef.*	*95% CI*		*p*	*Coef.*	*95% CI*		*p*	
**Active Travel**																					
GCSEs and lower	**ref*																				
School diploma	-0.03	-0.07	0.01	0.206	-0.04	-0.07	0.00	0.064	-0.01	-0.04	0.02	0.506	-0.03	-0.06	0.00	0.091	-0.04	-0.08	0.01	0.155	0.081
Degree/higher	-0.04	-0.08	0.00	0.044	-0.07	-0.10	-0.04	<0.001	-0.08	-0.10	-0.05	<0.001	-0.07	-0.10	-0.04	<0.001	-0.06	-0.11	-0.01	0.012	
**Occupational**																					
GCSEs and lower	**ref*																				
School diploma	-0.10	-0.14	-0.06	<0.001	-0.14	-0.18	-0.10	<0.001	-0.06	-0.10	-0.03	<0.001	-0.03	-0.07	0.00	0.060	-0.01	-0.06	0.04	0.648	0.001
Degree/higher	-0.25	-0.29	-0.21	<0.001	-0.29	-0.33	-0.26	<0.001	-0.26	-0.29	-0.23	<0.001	-0.24	-0.27	-0.21	<0.001	-0.20	-0.25	-0.15	<0.001	
**Moderate-to-vigorous leisure time**																					
GCSEs and lower	**ref*																				
School diploma	0.09	0.06	0.13	<0.001	0.08	0.04	0.11	<0.001	0.04	0.02	0.07	0.001	0.06	0.04	0.09	<0.001	0.06	0.04	0.08	<0.001	0.008
Degree/higher	0.17	0.14	0.21	<0.001	0.17	0.14	0.20	<0.001	0.16	0.14	0.19	<0.001	0.18	0.16	0.21	<0.001	0.16	0.14	0.18	<0.001	
**Light leisure time**																					
GCSEs and lower	**ref*																				
School diploma	0.02	-0.01	0.04	0.173	0.03	0.00	0.06	0.030	0.04	0.01	0.06	0.002	0.02	-0.01	0.04	0.180	0.06	0.04	0.08	<0.001	<0.001
Degree/higher	0.02	0.00	0.05	0.102	0.03	0.01	0.05	0.007	0.03	0.01	0.06	0.002	0.08	0.06	0.11	<0.001	0.13	0.11	0.15	<0.001	

Estimates are derived from separate logistic regression models of each binary physical activity outcome including a two-way interaction terms (education x age), and show the education-outcome associations on the absolute scale in each sociodemographic sub group
Participants from wave 5 (2013-2015) of Understanding Society with data on educational attainment, demographics, and physical activity
**reference groups*

For peer review only - http://bmjopen.bmj.com/site/about/guidelines.xhtml

Table S3. Estimated difference in predicted probability in educational differences in physical activity by sex

Physical activity	Male				Female				education X sex
	Coef.	*95% CI*		*p*	*Coef.*	*95% CI*		*p*	
**Active Travel**									
GCSEs and lower	**ref*								
School diploma	-0.02	-0.05	0.00	0.050	-0.03	-0.05	0.00	0.027	<0.001
Degree/higher	-0.03	-0.05	0.00	0.018	-0.10	-0.12	-0.08	0.000	
**Occupational**									
GCSEs and lower	**ref*								
School diploma	-0.06	-0.09	-0.04	<0.001	-0.08	-0.11	-0.06	<0.001	<0.001
Degree/higher	-0.35	-0.37	-0.32	<0.001	-0.17	-0.19	-0.15	<0.001	
**Moderate-to-vigorous leisure time**									
GCSEs and lower	**ref*								
School diploma	0.06	0.05	0.08	<0.001	0.07	0.05	0.08	<0.001	0.550
Degree/higher	0.17	0.15	0.18	<0.001	0.17	0.15	0.18	<0.001	
**Light leisure time**									
GCSEs and lower	**ref*								
School diploma	0.03	0.01	0.04	0.001	0.05	0.03	0.06	<0.001	<0.001
Degree/higher	0.02	0.01	0.03	0.005	0.12	0.10	0.13	<0.001	

Estimates are derived from separate logistic regression models of each binary physical activity outcome including a two-way interaction terms (education x sex), and show the education-outcome associations on the absolute scale in each sociodemographic sub group
Participants from wave 5 (2013-2015) of Understanding Society with data on educational attainment, demographics, and physical activity
**reference group*

For peer review only

Table S4. Estimated difference in predicted probability in educational differences in physical activity by ethnicity

Physical activity	White				Black				Asian				Other ethnicity				education X ethnicity
	Coef.	*95% CI*		*p*	*Coef.*	*95% CI*		*p*	*Coef.*	*95% CI*		*p*	*Coef.*	*95% CI*		*p*	P
**Active Travel**																	
GCSEs and lower	**ref*																
School diploma	-0.02	-0.04	0.00	0.013	0.02	-0.08	0.11	0.723	-0.07	-0.13	0.00	0.040	-0.04	-0.17	0.08	0.523	0.038
Degree/higher	-0.06	-0.08	-0.04	<0.001	-0.12	-0.20	-0.05	0.002	-0.11	-0.17	-0.05	<0.001	-0.04	-0.15	0.07	0.465	
**Occupational**																	
GCSEs and lower	**ref*																
School diploma	-0.08	-0.10	-0.06	<0.001	-0.03	-0.12	0.06	0.526	-0.07	-0.13	0.00	0.035	-0.04	-0.17	0.08	0.502	0.155
Degree/higher	-0.27	-0.29	-0.25	<0.001	-0.14	-0.22	-0.06	0.001	-0.18	-0.24	-0.13	<0.001	-0.19	-0.30	-0.08	0.001	
**Moderate-to-vigorous leisure time**																	
GCSEs and lower	**ref*																
School diploma	0.06	0.05	0.08	<0.001	0.02	-0.04	0.08	0.488	0.09	0.06	0.12	<0.001	0.12	0.04	0.20	0.004	0.001
Degree/higher	0.17	0.16	0.19	<0.001	0.06	0.01	0.12	0.030	0.16	0.13	0.19	<0.001	0.13	0.06	0.20	<0.001	
**Light leisure time**																	
GCSEs and lower	**ref*																
School diploma	0.04	0.02	0.05	<0.001	0.05	0.00	0.09	0.046	0.04	0.01	0.07	0.008	0.08	0.01	0.14	0.026	0.057
Degree/higher	0.07	0.06	0.08	<0.001	0.02	-0.02	0.06	0.351	0.07	0.05	0.10	<0.001	0.12	0.06	0.17	<0.001	

Estimates are derived from separate logistic regression models of each binary physical activity outcome including a two-way interaction terms (education x ethnicity), and show the education-outcome associations on the absolute scale in each sociodemographic sub group
Participants from wave 5 (2013-2015) of Understanding Society with data on educational attainment, demographics, and physical activity
**reference group*

Table S5. Unadjusted associations (OR (95% CI)) of education and demographics with domain specific PA (complete case analysis)

	Physical Activity															
	Active travel				Occupational				Leisure							
									Moderate-to-vigorous				Light			
	N=18,404				*N=22,287*				*N= 40,270*				*N= 40,270*			
	OR	*95% CI*		*p*	*OR*	*95% CI*		*p*	*OR*	*95% CI*		*p*	*OR*	*95% CI*		*p*
**Education**																
GCSEs and lower	**ref*															
School diploma	0.86	0.77	0.96	0.006	0.75	0.69	0.81	<0.001	1.59	1.50	1.69	<0.001	1.23	1.16	1.31	<0.001
Degree/higher	0.62	0.55	0.68	<0.001	0.35	0.33	0.38	<0.001	2.55	2.41	2.69	<0.001	1.41	1.33	1.50	<0.001
**Ethnicity**																
White	**ref*															
Black	0.89	0.71	1.13	0.358	1.87	1.59	2.20	<0.001	0.84	0.75	0.95	0.005	0.38	0.31	0.45	<0.001
Asian	1.07	0.92	1.25	0.388	1.38	1.23	1.55	<0.001	0.74	0.68	0.81	<0.001	0.47	0.42	0.52	<0.001
Other ethnicity	1.25	0.97	1.61	0.080	1.20	0.99	1.44	0.061	1.08	0.94	1.25	0.277	0.66	0.55	0.80	<0.001
**Sex**																
Male	**ref*															
Female	1.09	1.01	1.19	0.033	0.99	0.94	1.05	0.822	0.85	0.82	0.89	<0.001	1.17	1.12	1.23	<0.001
**Age**																
20-29 years	**ref*															
30-39 years	0.75	0.65	0.85	<0.001	0.68	0.62	0.75	<0.001	1.01	0.93	1.09	0.875	1.05	0.95	1.16	0.353
40-49 years	0.68	0.60	0.77	<0.001	0.69	0.63	0.76	<0.001	0.84	0.78	0.90	<0.001	1.38	1.25	1.51	<0.001
50-59 years	0.76	0.67	0.87	<0.001	0.78	0.71	0.85	<0.001	0.60	0.56	0.65	<0.001	1.56	1.42	1.71	<0.001
60+ years	0.70	0.58	0.83	<0.001	0.88	0.79	0.99	0.034	0.43	0.39	0.46	<0.001	1.64	1.50	1.80	<0.001

Participants from wave 5 (2013-2015) of Understanding Society with data on educational attainment, demographics, and physical activity
Samples were restricted to those with valid demographic and physical activity data
*Active travel to work: non-active/active; Occupational: non-physical/physical; Leisure-time: <weekly/ ≥ 1x weekly*reference groups*
OR= Odds Ratio, CI= 95% Confidence intervals

Table S6. Unadjusted associations (OR (95% CI)) of education and demographics with domain specific PA (maximum available samples)

	Physical Activity																			
	Active travel					Occupational					Leisure									
											Moderate-to-vigorous					Light				
	N	*OR*	*95% CI*		*p*	N	*OR*	*95% CI*		*p*	N	*OR*	*95% CI*		*p*	N	*OR*	*95% CI*		*p*
**Education**																				
GCSEs and lower	18535	**ref*				22431					41700					41700				
School diploma		0.86	0.77	0.96	0.006		0.75	0.69	0.81	<0.001		1.59	1.50	1.69	<0.001		1.23	1.16	1.31	<0.001
Degree/higher		0.62	0.55	0.69	<0.001		0.35	0.33	0.38	<0.001		2.56	2.43	2.70	<0.001		1.43	1.35	1.52	<0.001
**Ethnicity**																				
White	18430	**ref*				22317					40364					40364				
Black		0.89	0.71	1.13	0.360		1.87	1.59	2.20	<0.001		0.84	0.75	0.95	0.005		0.38	0.31	0.45	<0.001
Asian		1.07	0.92	1.25	0.385		1.38	1.23	1.55	<0.001		0.74	0.68	0.81	<0.001		0.47	0.42	0.52	<0.001
Other ethnicity		1.25	0.97	1.61	0.080		1.20	0.99	1.44	0.062		1.08	0.94	1.25	0.282		0.66	0.55	0.80	<0.001
**Sex**																				
Male	18568	**ref*				22470					41853					41853				
Female		1.09	1.00	1.18	0.039		0.99	0.94	1.04	0.688		0.90	0.86	0.93	<0.001		1.22	1.17	1.28	<0.001
**Age**																				
20-29 years	18568	**ref*				22470					41853					41853				
30-39 years		0.74	0.65	0.85	<0.001		0.68	0.62	0.75	<0.001		1.04	0.96	1.12	0.380		1.07	0.97	1.18	0.192
40-49 years		0.68	0.60	0.77	<0.001		0.69	0.63	0.75	<0.001		0.87	0.81	0.94	<0.001		1.40	1.28	1.54	<0.001
50-59 years		0.76	0.67	0.87	<0.001		0.77	0.71	0.84	<0.001		0.63	0.59	0.68	<0.001		1.58	1.45	1.74	<0.001
60+ years		0.70	0.59	0.84	<0.001		0.88	0.79	0.99	0.031		0.45	0.42	0.48	<0.001		1.69	1.54	1.84	<0.001

Participants from wave 5 (2013-2015) of Understanding Society with data on educational attainment, demographics, and physical activity
Sample size was not restricted and represents total available sample
*Active travel to work: non-active/active; Occupational: non-physical/physical; Leisure-time: <weekly/ ≥ 1x weekly*reference groups*
OR= Odds Ratio, CI= 95% Confidence intervals

For peer review only - http://bmjopen.bmj.com/site/about/guidelines.xhtml

Figure S1. Flow diagram of analyses in Understanding Society

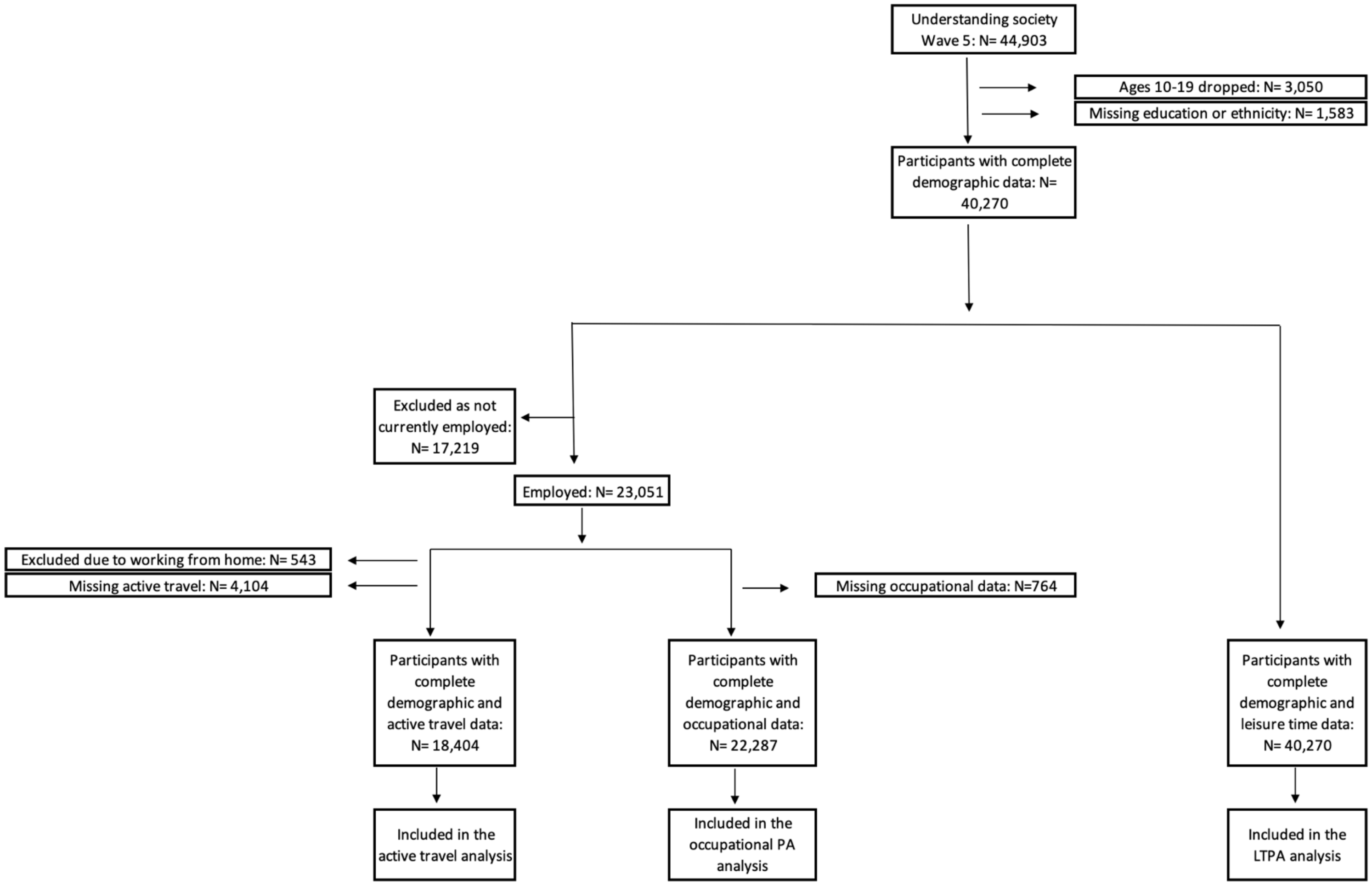

There was no missing data for sex and age demographics.
Active travel and occupational physical activity only include individuals who are currently employed, due to questionnaire routing.

Reporting checklist for cross sectional study.

Based on the STROBE cross sectional guidelines.

Instructions to authors

Complete this checklist by entering the page numbers from your manuscript where readers will find each of the items listed below.

Your article may not currently address all the items on the checklist. Please modify your text to include the missing information. If you are certain that an item does not apply, please write "n/a" and provide a short explanation.

Upload your completed checklist as an extra file when you submit to a journal.

In your methods section, say that you used the STROBE cross sectionalreporting guidelines, and cite them as:

von Elm E, Altman DG, Egger M, Pocock SJ, Gotzsche PC, Vandenbroucke JP. The Strengthening the Reporting of Observational Studies in Epidemiology (STROBE) Statement: guidelines for reporting observational studies.

For peer review only

		Reporting Item	Page Number
**Title and abstract**			
Title	#1a	Indicate the study's design with a commonly used term in the title or the abstract	1
Abstract	#1b	Provide in the abstract an informative and balanced summary of what was done and what was found	2
**Introduction**			
Background / rationale	#2	Explain the scientific background and rationale for the investigation being reported	4
Objectives	#3	State specific objectives, including any prespecified hypotheses	5
**Methods**			

Study design	#4	Present key elements of study design early in the paper	6/7
Setting	#5	Describe the setting, locations, and relevant dates, including periods of recruitment, exposure, follow-up, and data collection	6
Eligibility criteria	#6a	Give the eligibility criteria, and the sources and methods of selection of participants.	6/7
	#7	Clearly define all outcomes, exposures, predictors, potential confounders, and effect modifiers. Give diagnostic criteria, if applicable	6/7
Data sources / measurement	#8	For each variable of interest give sources of data and details of methods of assessment (measurement). Describe comparability of assessment methods if there is more than one group. Give information separately for for exposed and unexposed groups if applicable.	6/7
Bias	#9	Describe any efforts to address potential sources of bias	7
Study size	#10	Explain how the study size was arrived at	7
Quantitative variables	#11	Explain how quantitative variables were handled in the analyses. If applicable, describe which groupings were chosen, and why	6/7
Statistical methods	#12a	Describe all statistical methods, including those used to control for confounding	7
Statistical methods	#12b	Describe any methods used to examine subgroups and interactions	7
Statistical methods	#12c	Explain how missing data were addressed	7
Statistical methods	#12d	If applicable, describe analytical methods taking account of sampling strategy	6/7
Statistical methods	#12e	Describe any sensitivity analyses	n/a

Results

For peer review only

Participants	#13a	Report numbers of individuals at each stage of study—eg numbers potentially eligible, examined for eligibility, confirmed eligible, included in the study, completing follow-up, and analysed. Give information separately for for exposed and unexposed groups if applicable.	8, 13, 21, 27
Participants	#13b	Give reasons for non-participation at each stage	27
Participants	#13c	Consider use of a flow diagram	27
Descriptive data	#14a	Give characteristics of study participants (eg demographic, clinical, social) and information on exposures and potential confounders. Give information separately for exposed and unexposed groups if applicable.	8, 21
Descriptive data	#14b	Indicate number of participants with missing data for each variable of interest	8, 13, 21
Outcome data	#15	Report numbers of outcome events or summary measures. Give information separately for exposed and unexposed groups if applicable.	8, 13, 21
Main results	#16a	Give unadjusted estimates and, if applicable, confounder-adjusted estimates and their precision (eg, 95% confidence interval). Make clear which confounders were adjusted for and why they were included	14, 22, 23
Main results	#16b	Report category boundaries when continuous variables were categorized	n/a
Main results	#16c	If relevant, consider translating estimates of relative risk into absolute risk for a meaningful time period	n/a
Other analyses	#17	Report other analyses done—e.g., analyses of subgroups and interactions, and sensitivity analyses	n/a
**Discussion**			
Key results	#18	Summarise key results with reference to study objectives	10
Limitations	#19	Discuss limitations of the study, taking into account sources of potential bias or imprecision. Discuss both direction and magnitude of any potential bias.	10/11

For peer review only

Interpretation	#20	Give a cautious overall interpretation considering objectives, limitations, multiplicity of analyses, results from similar studies, and other relevant evidence.	11/12
Generalisability	#21	Discuss the generalisability (external validity) of the study results	10
**Other Information**			
Funding	#22	Give the source of funding and the role of the funders for the present study and, if applicable, for the original study on which the present article is based	20

None The STROBE checklist is distributed under the terms of the Creative Commons Attribution License CC-BY. This checklist can be completed online using https://www.goodreports.org/, a tool made by the EQUATOR Network in collaboration with Penelope.ai

For peer review only
